# Supplementary figures and images for: Proteolytic cleavage and inactivation of the TRMT1 tRNA modification enzyme by SARS-CoV-2 main protease (part 1 of 2)
Source: eLife. 2024 May 30;12:RP90316. doi: 10.7554/eLife.90316 (PMC11139479; doi:10.7554/eLife.90316)

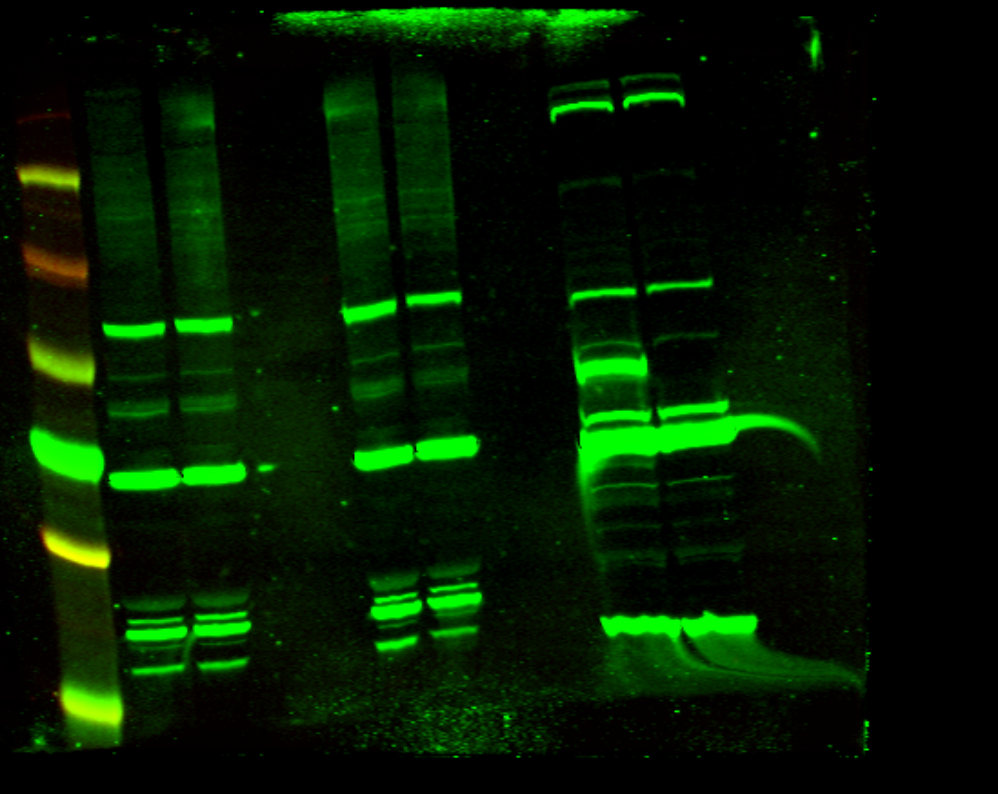

Supplement: Figure 1—source data 1. [file elife-90316-fig1-data1.zip › Figure 1A-20220721-TRMT1G3-MRC5mockSARS24h48h 293WT KONSP5.tif]

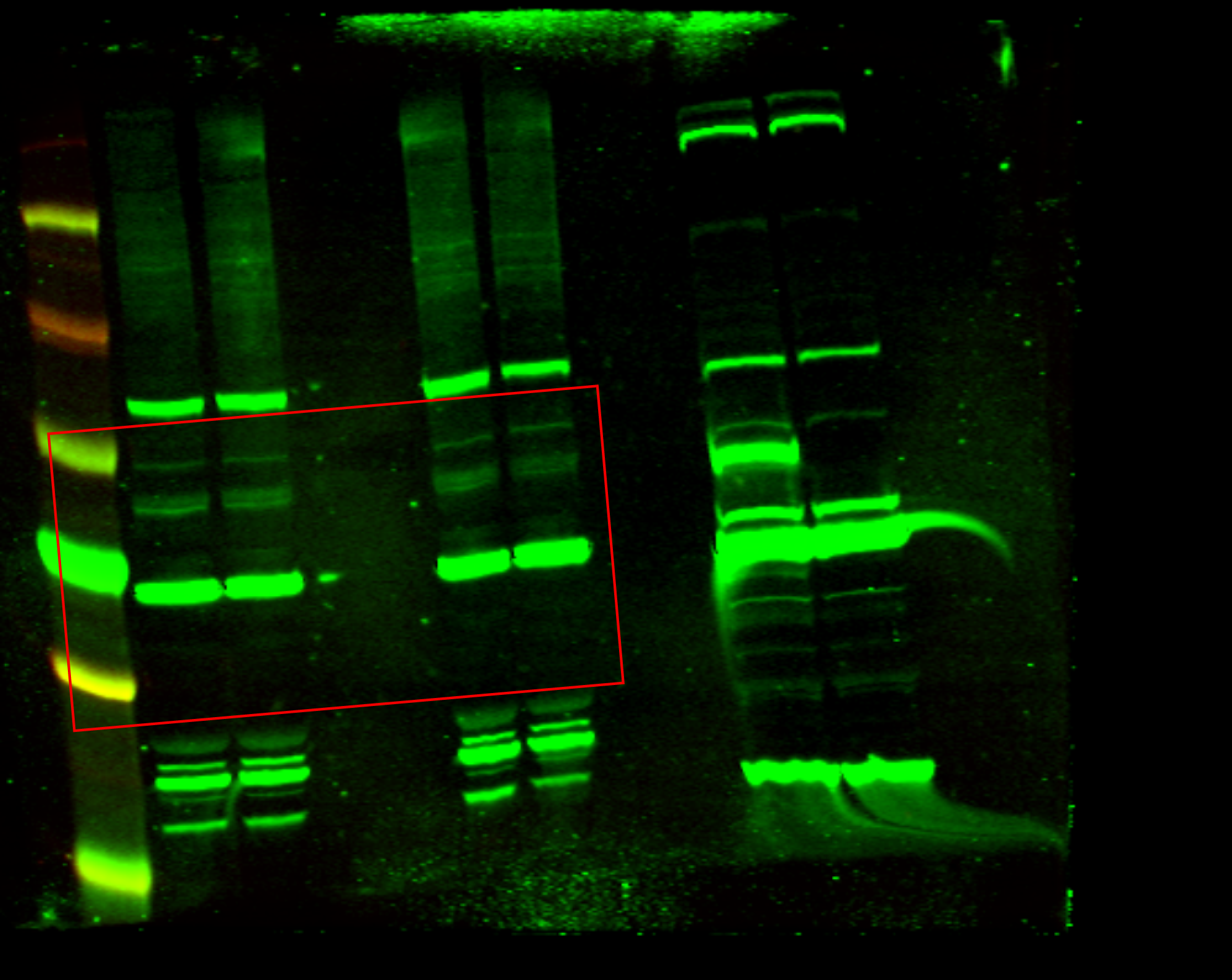

Supplement: Figure 1—source data 1. [file elife-90316-fig1-data1.zip › Figure 1A-20220721-TRMT1G3-MRC5mockSARS24h48h 293WT KONSP5 labeled.tif]

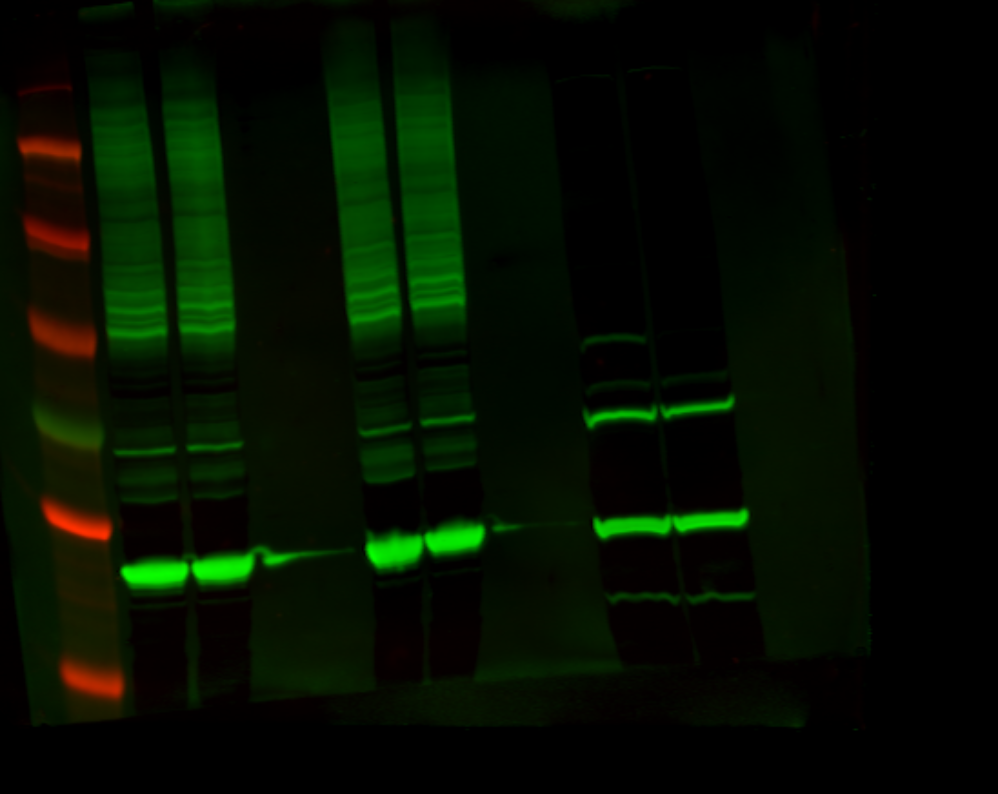

Supplement: Figure 1—source data 1. [file elife-90316-fig1-data1.zip › FIgure 1A-20220722-Actin-MRC5mockSARS6h24h 293WT KONSP5.tif]

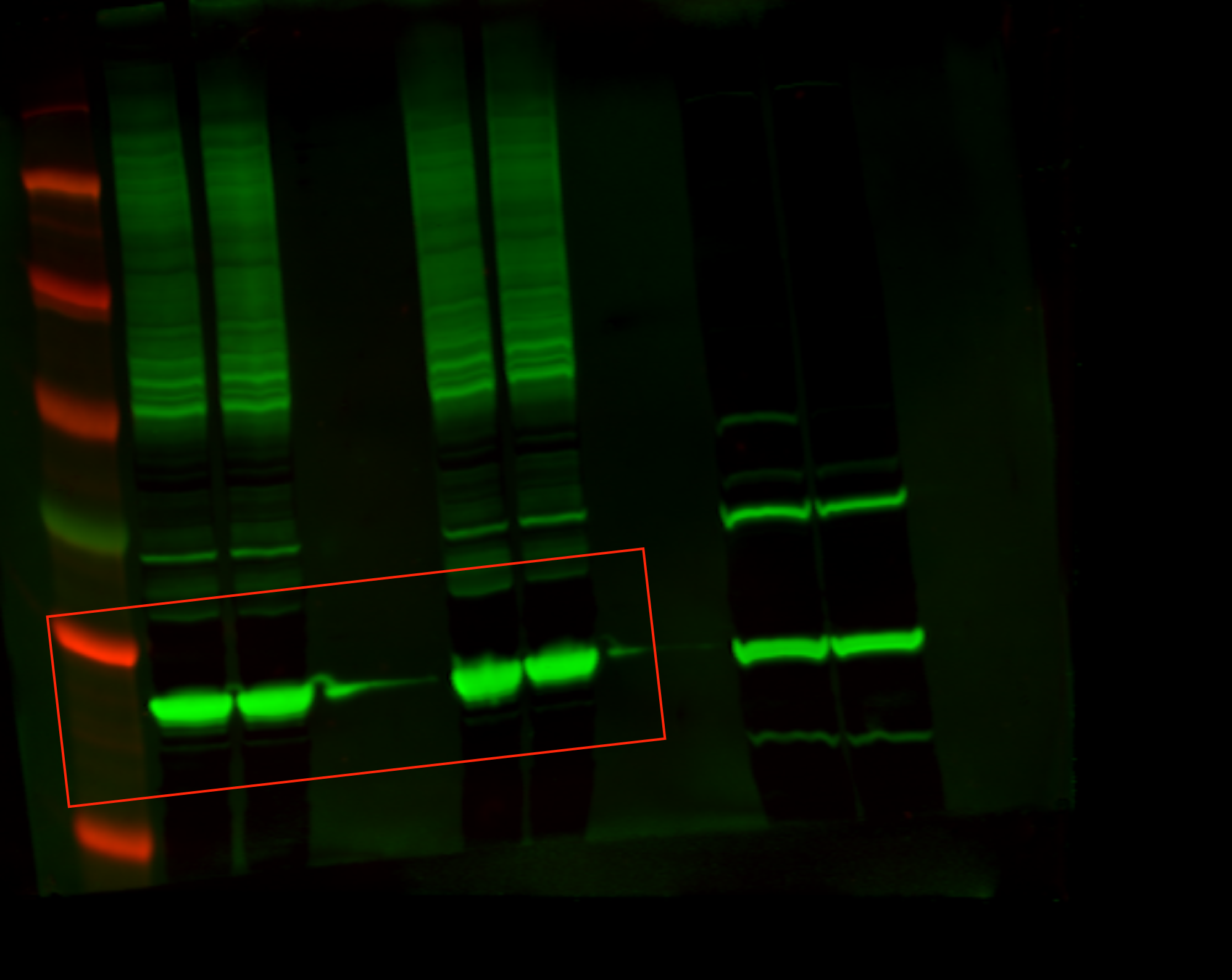

Supplement: Figure 1—source data 1. [file elife-90316-fig1-data1.zip › FIgure 1A-20220722-Actin-MRC5mockSARS6h24h 293WT KONSP5 labeled.tif]

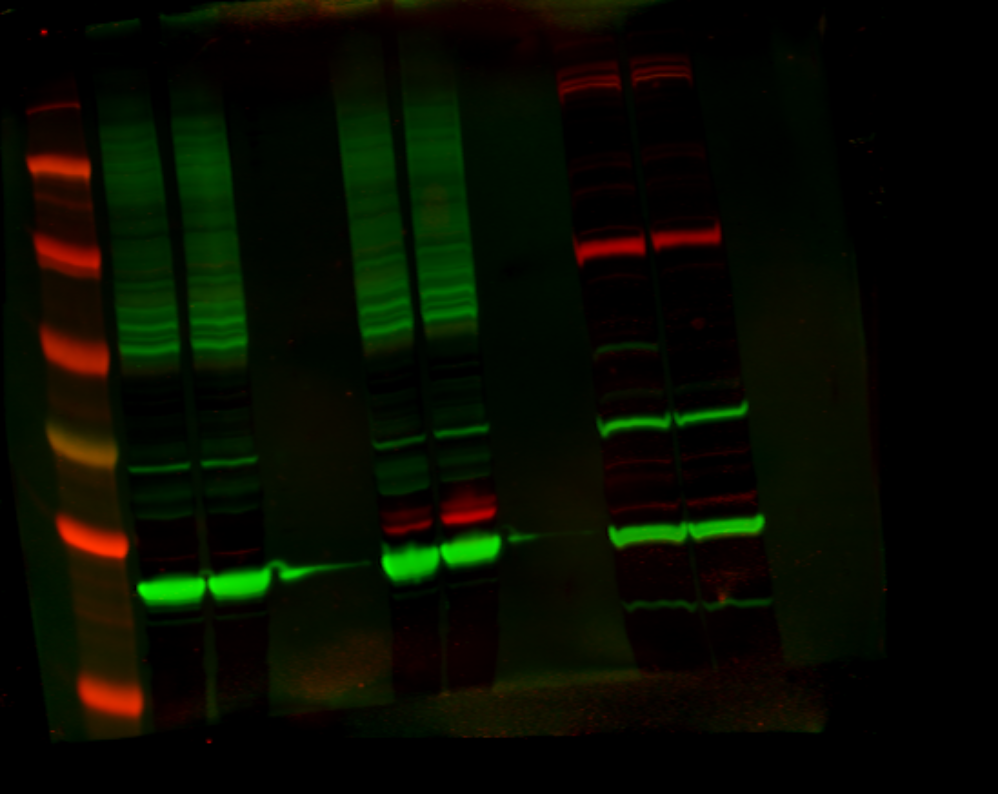

Supplement: Figure 1—source data 1. [file elife-90316-fig1-data1.zip › FIgure 1A-20220723-SARSNprotein-MRC5mockSARS6h24h 293WT KONSP5.tif]

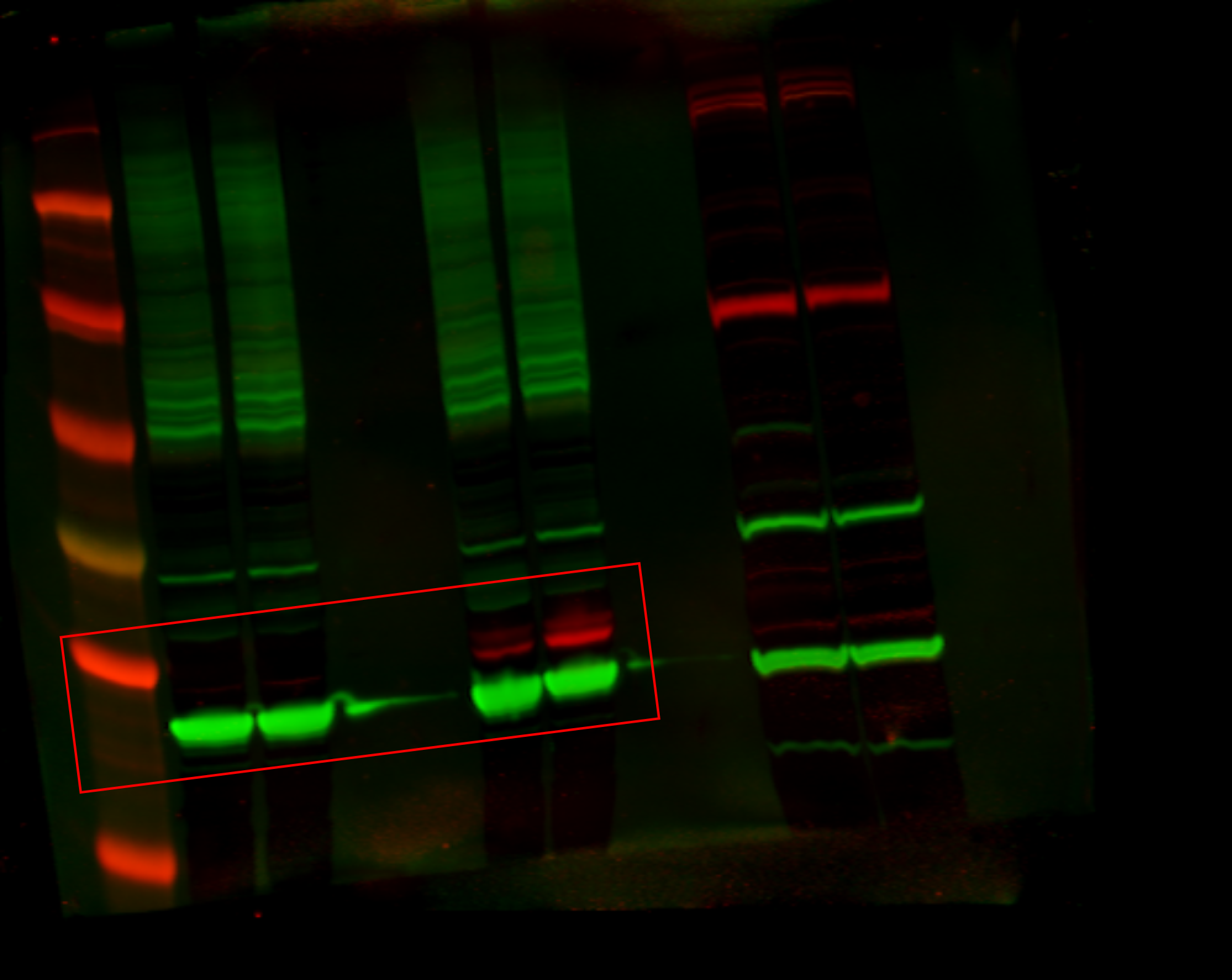

Supplement: Figure 1—source data 1. [file elife-90316-fig1-data1.zip › FIgure 1A-20220723-SARSNprotein-MRC5mockSARS6h24h 293WT KONSP5 labeled.tif]

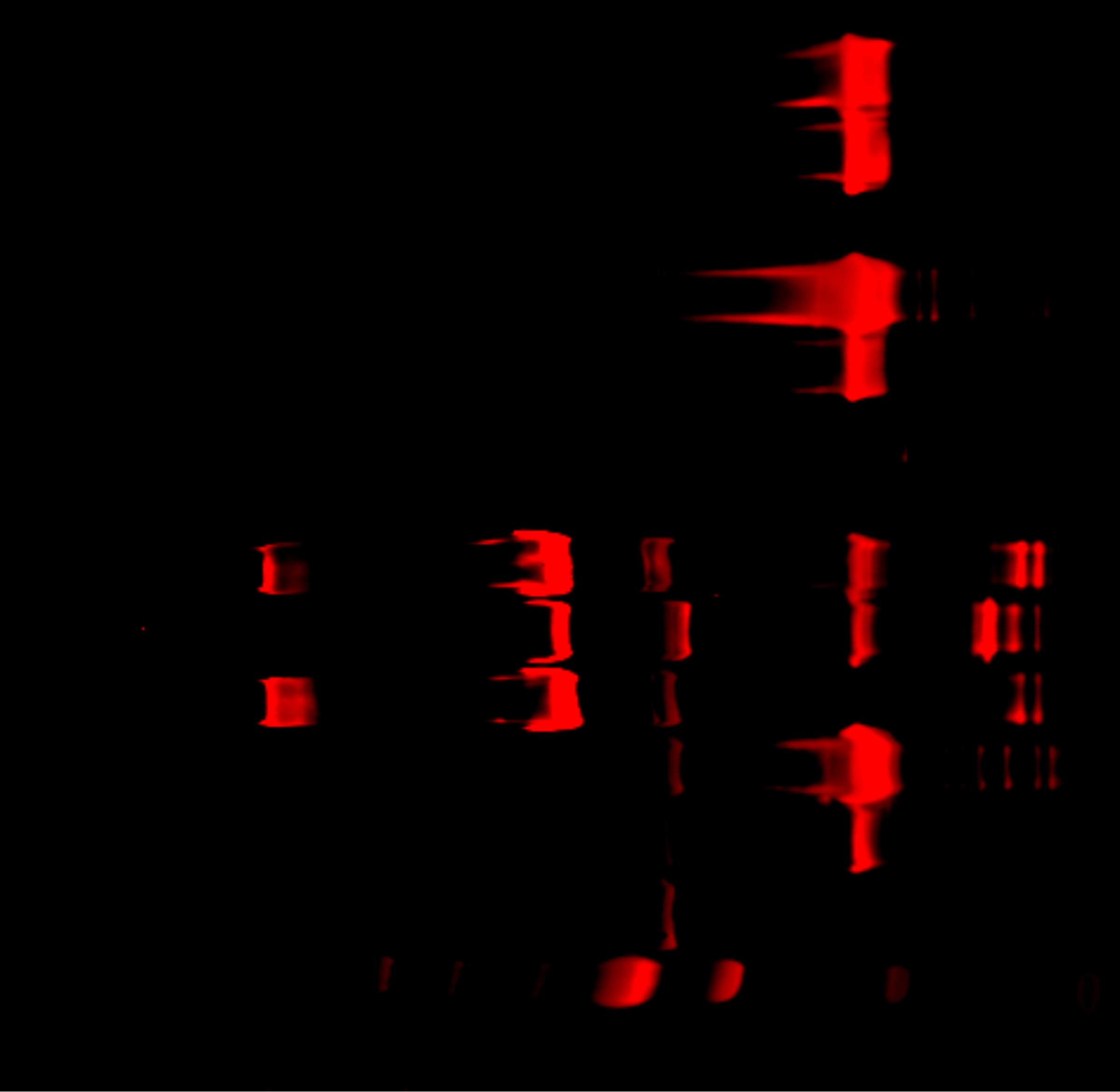

Supplement: Figure 2—source data 1. [file elife-90316-fig2-data1.zip › Figuer 2D antiSTREP 680.tif]

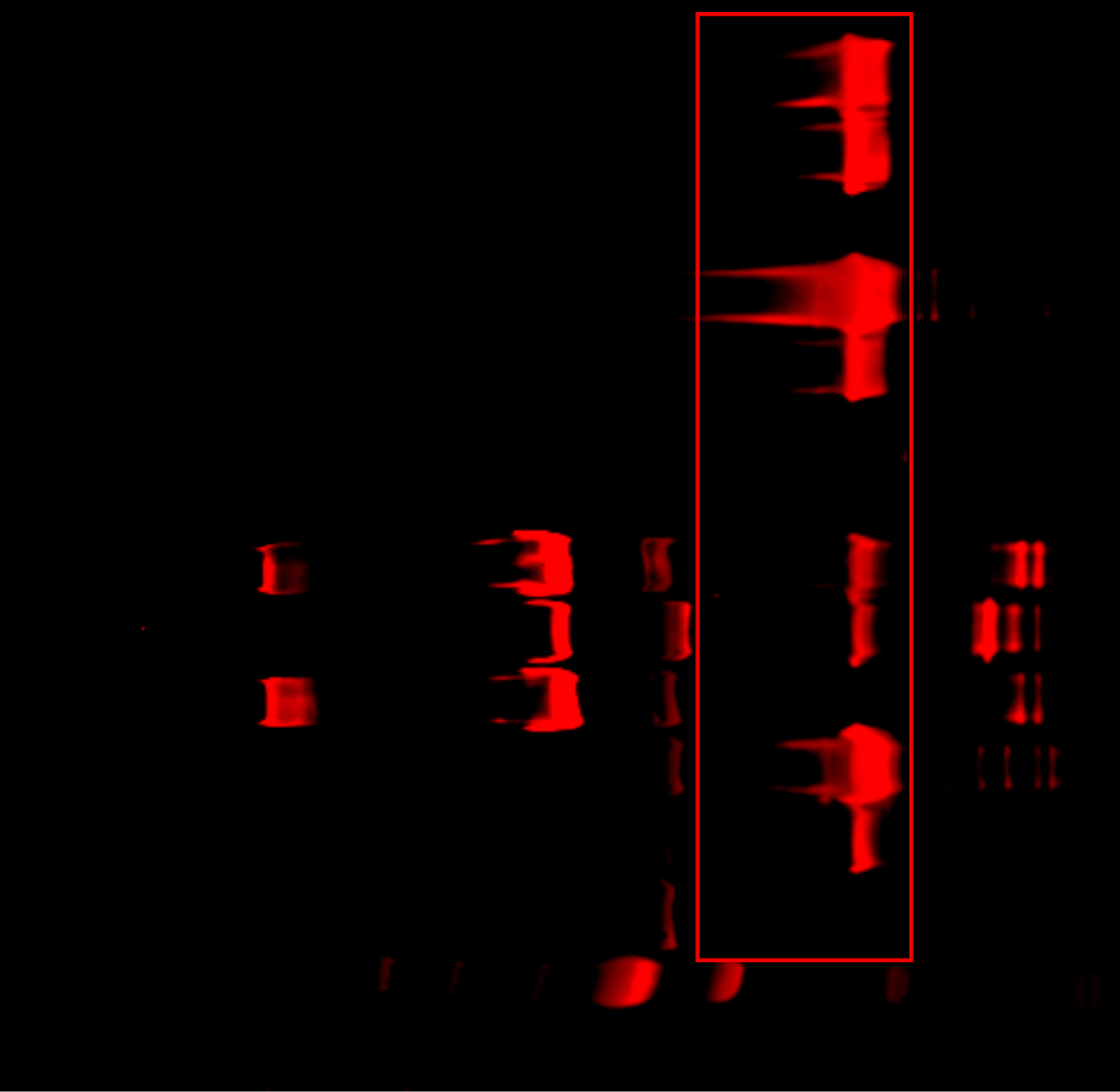

Supplement: Figure 2—source data 1. [file elife-90316-fig2-data1.zip › Figuer 2D antiSTREP 680 labeled.tif]

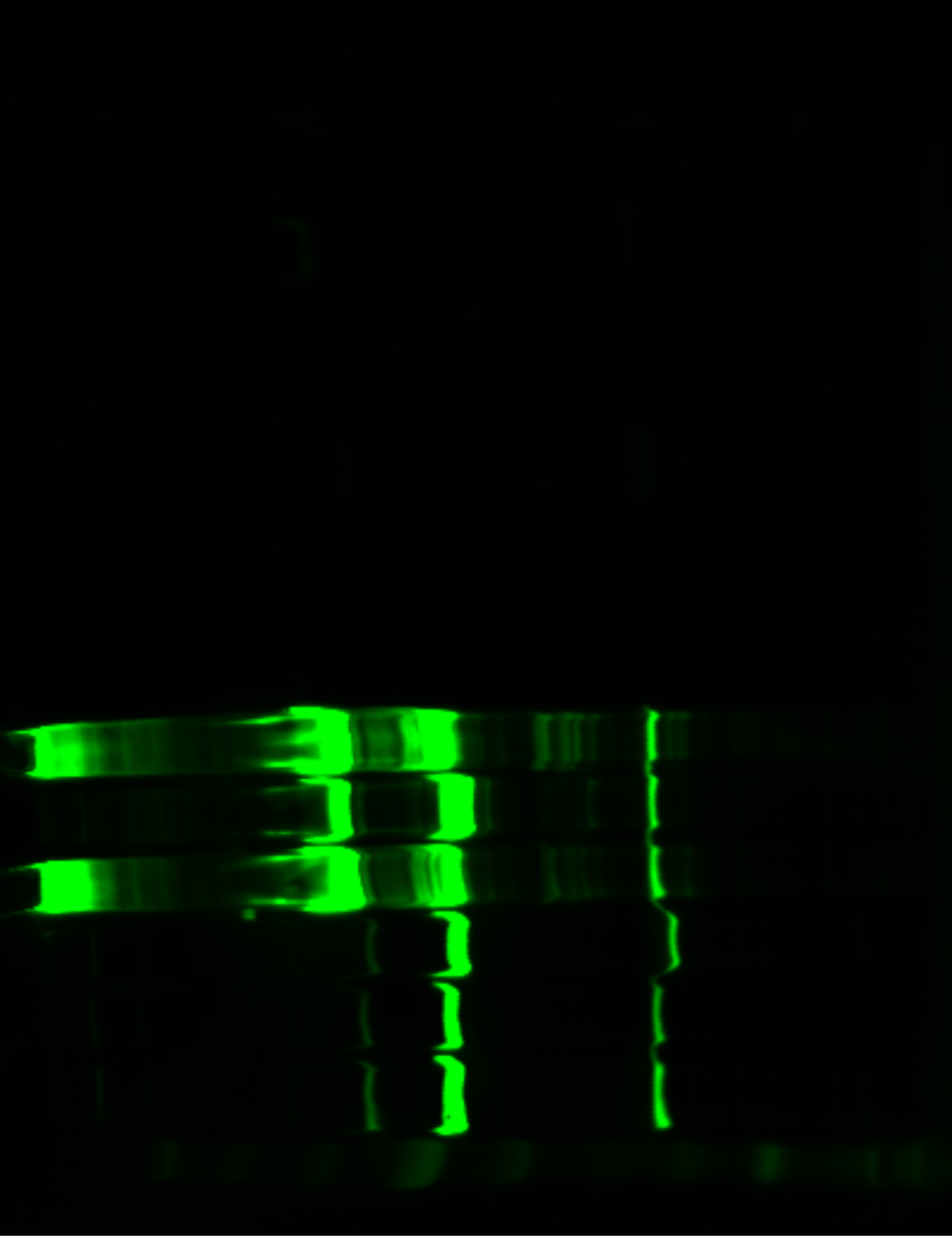

Supplement: Figure 2—source data 1. [file elife-90316-fig2-data1.zip › Figure 2D anti TRMT1 800.tif]

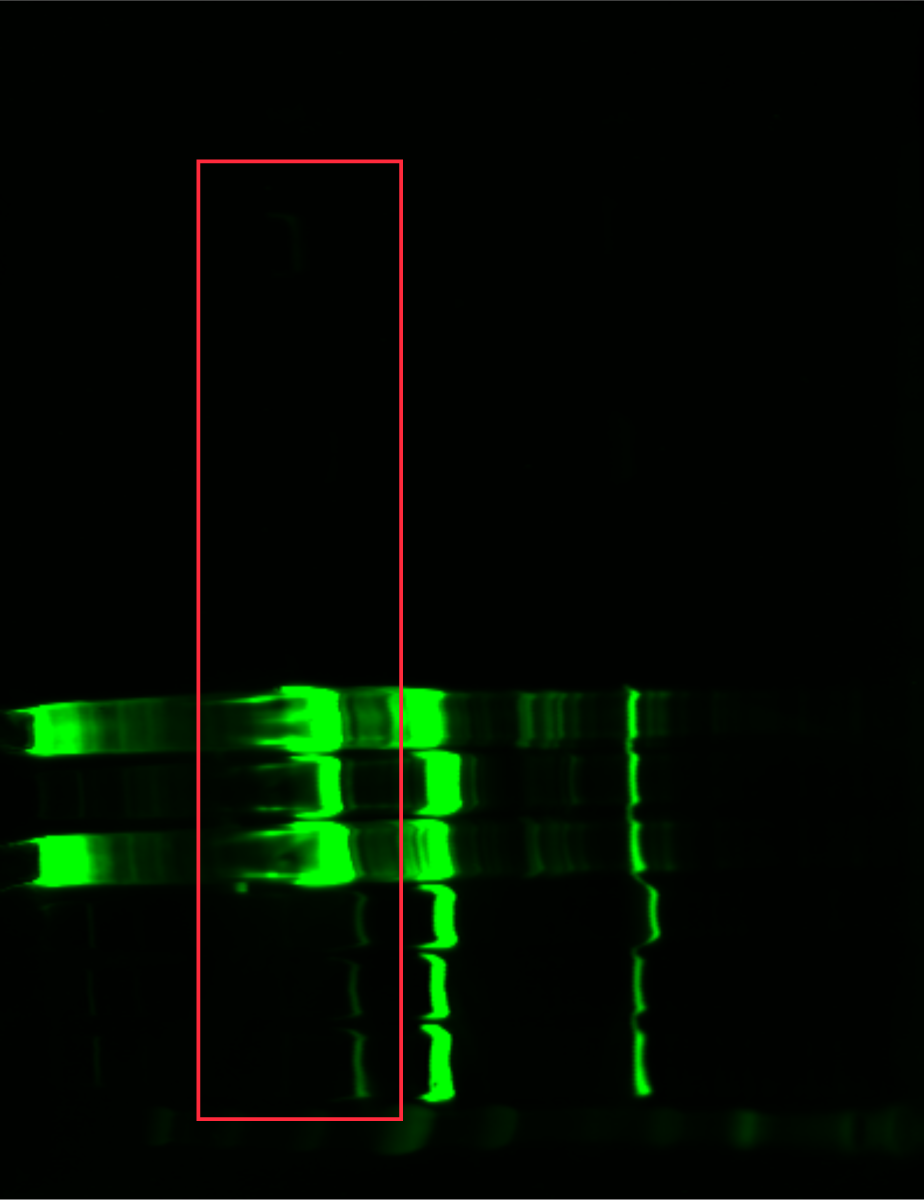

Supplement: Figure 2—source data 1. [file elife-90316-fig2-data1.zip › Figure 2D anti TRMT1 800 labeled.tif]

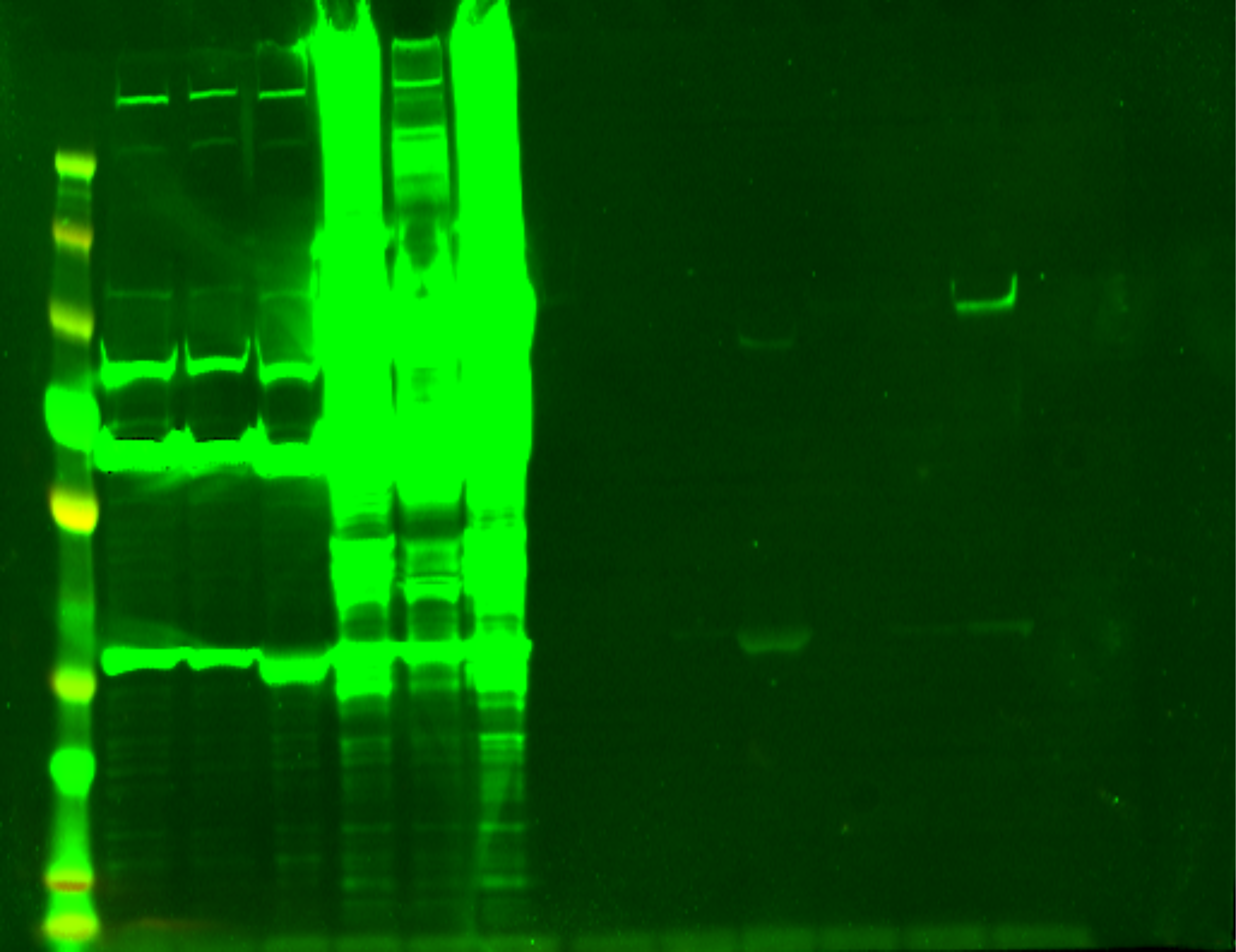

Supplement: Figure 2—source data 1. [file elife-90316-fig2-data1.zip › Figure 2D anti-TRMT1 higher exposure.tif]

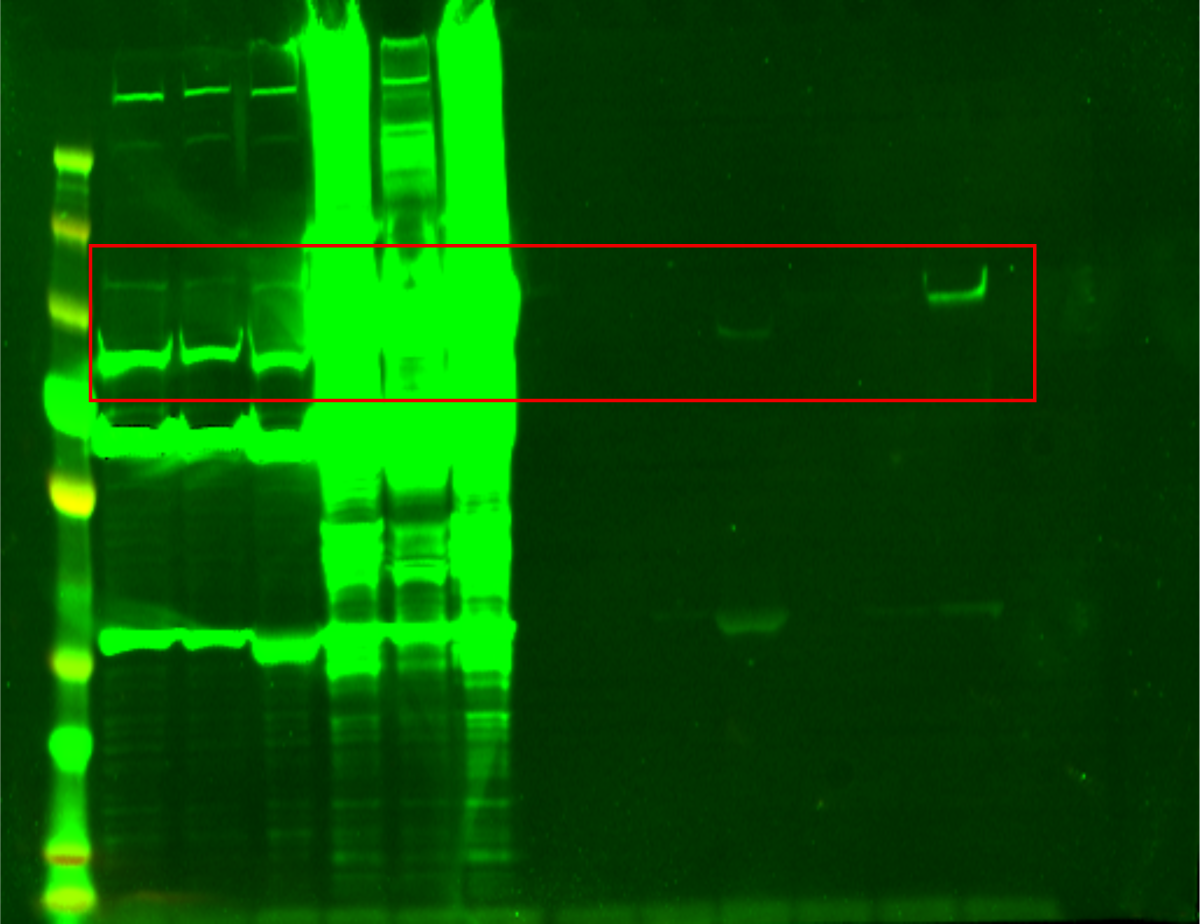

Supplement: Figure 2—source data 1. [file elife-90316-fig2-data1.zip › Figure 2D anti-TRMT1 higher exposure labeled.tif]

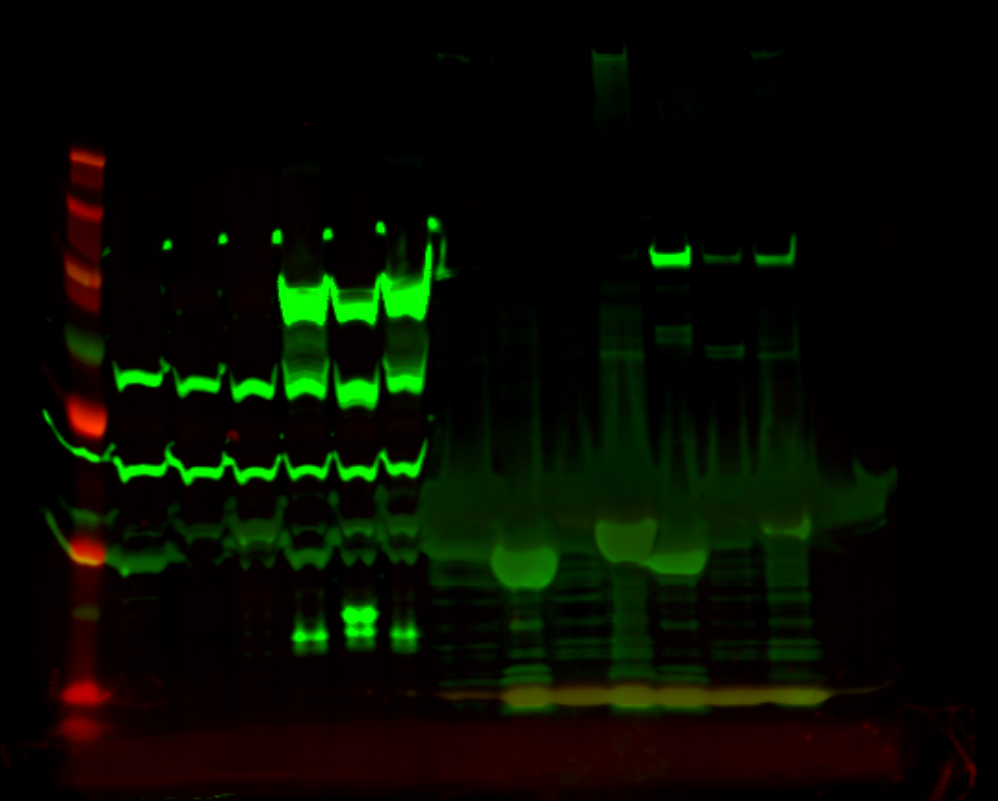

Supplement: Figure 2—figure supplement 1—source data 1. [file elife-90316-fig2-figsupp1-data1.zip › 072921-Actin-purification of Strep Nsp5.tif]

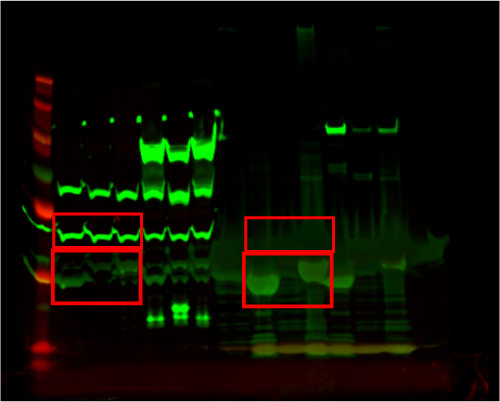

Supplement: Figure 2—figure supplement 1—source data 1. [file elife-90316-fig2-figsupp1-data1.zip › 072921-Actin-purification of Strep Nsp5 labeled.tif]

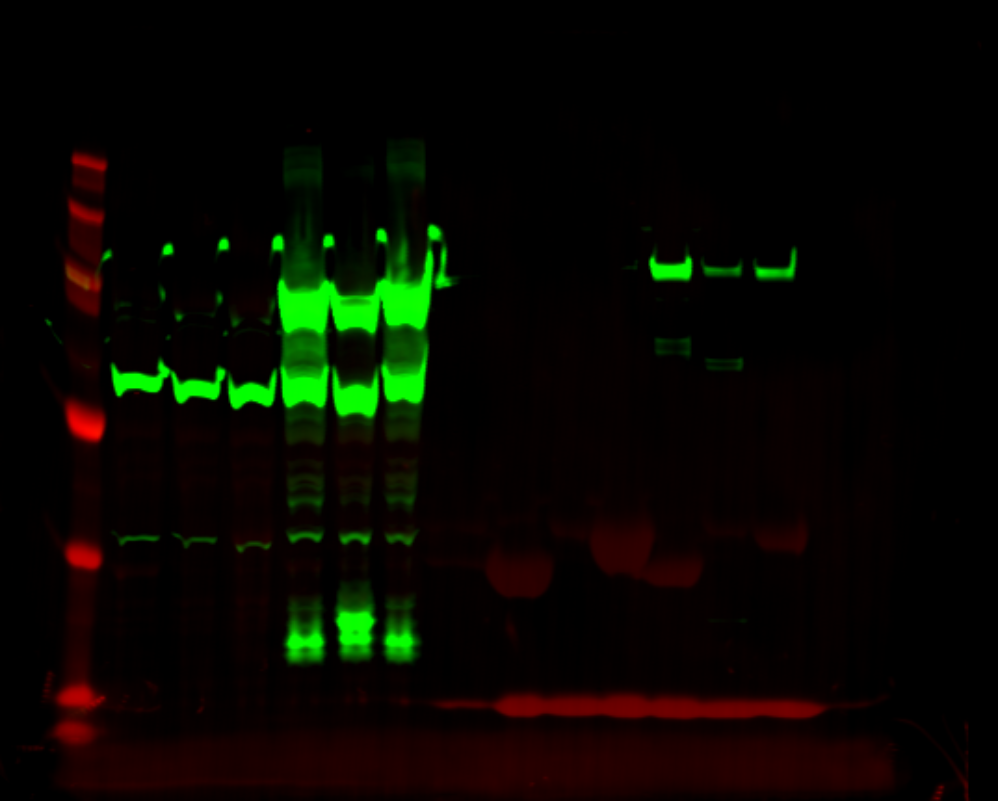

Supplement: Figure 2—figure supplement 1—source data 1. [file elife-90316-fig2-figsupp1-data1.zip › 072921-Flag-purification of Strep Nsp5.tif]

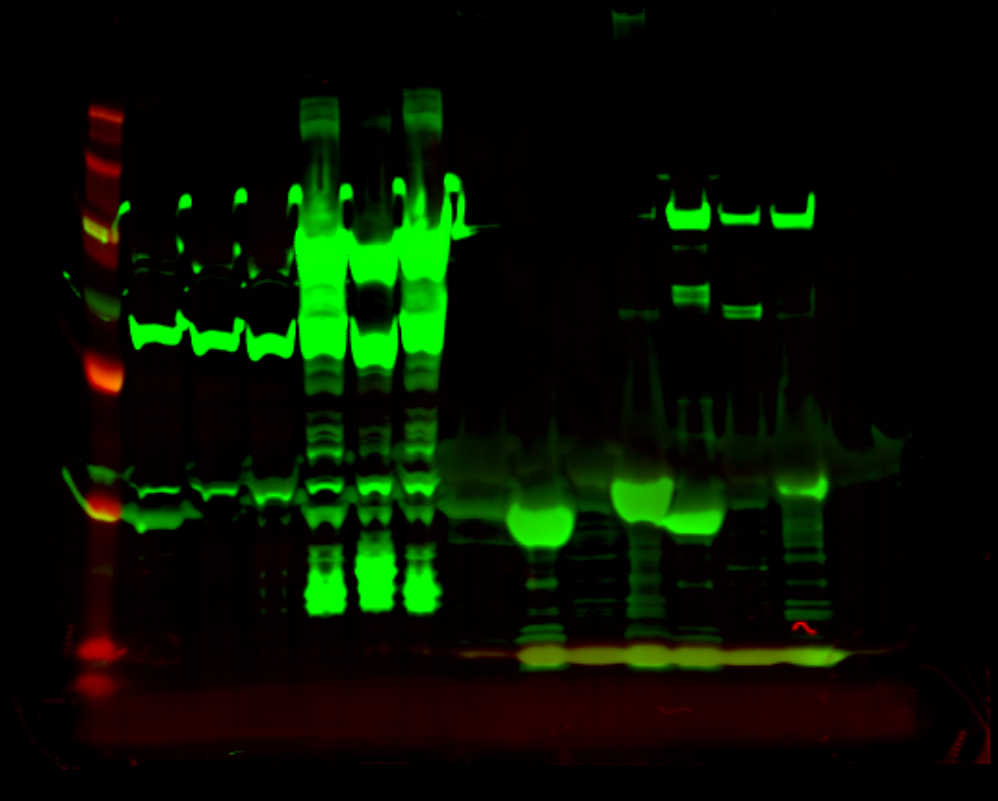

Supplement: Figure 2—figure supplement 1—source data 1. [file elife-90316-fig2-figsupp1-data1.zip › 072921-Strep-purification of Strep Nsp5.tif]

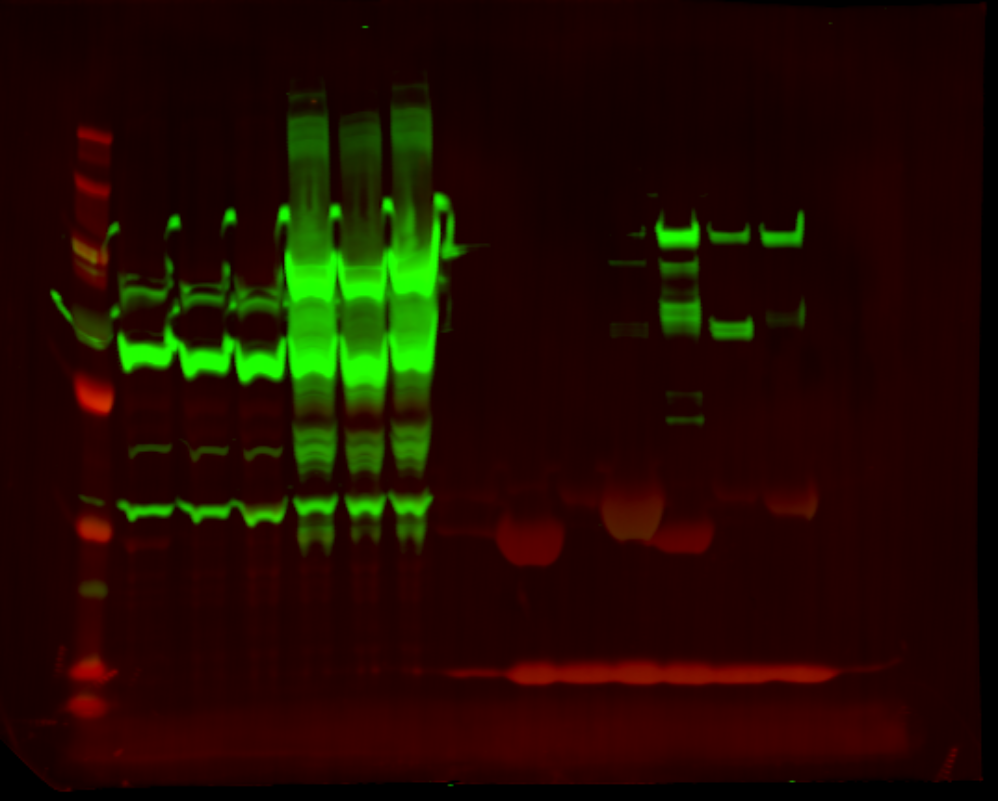

Supplement: Figure 2—figure supplement 1—source data 1. [file elife-90316-fig2-figsupp1-data1.zip › 072921-TRMT1G3-purification of Strep Nsp5.tif]

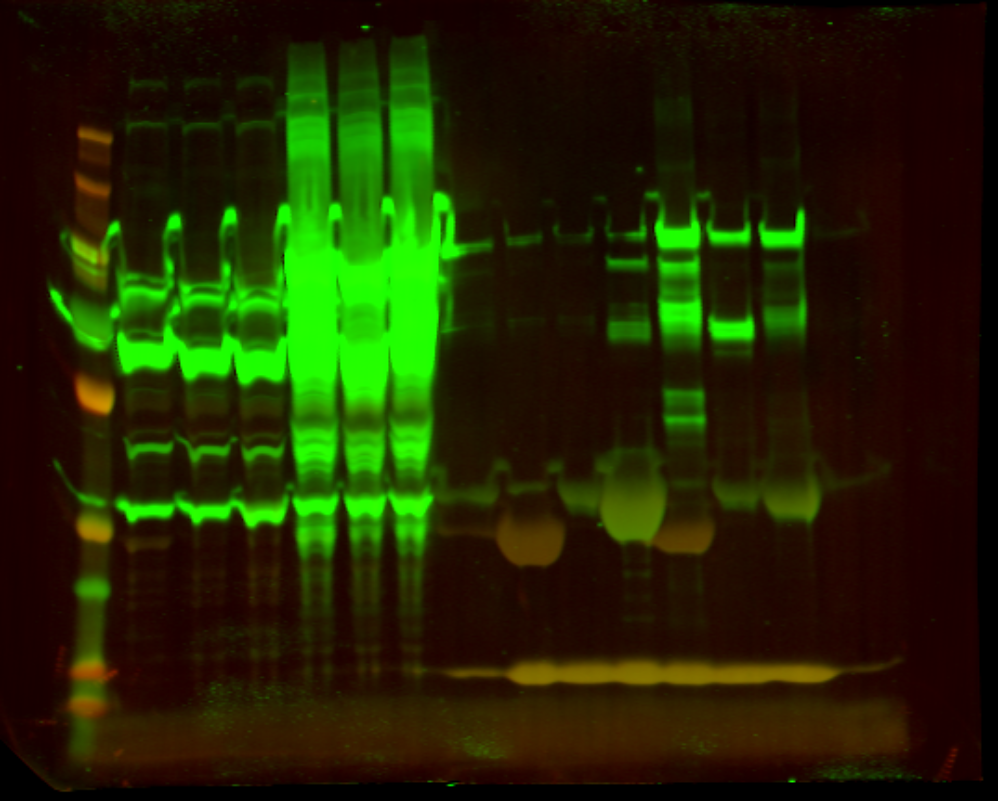

Supplement: Figure 2—figure supplement 1—source data 1. [file elife-90316-fig2-figsupp1-data1.zip › 072921-TRMT1G3-purification of Strep Nsp5- Overexpose.tif]

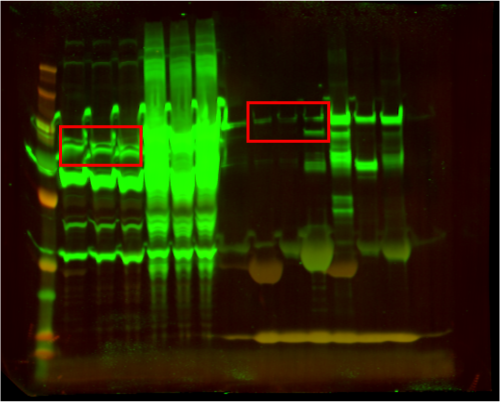

Supplement: Figure 2—figure supplement 1—source data 1. [file elife-90316-fig2-figsupp1-data1.zip › 072921-TRMT1G3-purification of Strep Nsp5- Overexpose labeled.tif]

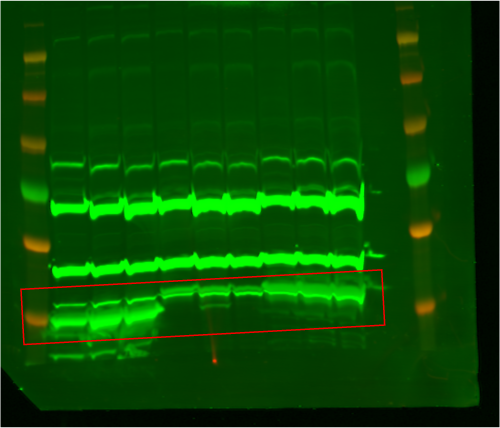

Supplement: Figure 3—source data 1. [file elife-90316-fig3-data1.zip › 3A/Figure 3A 100321-Strep-293T conNSP5C145A transfection 24 48 72h labeled.tif]

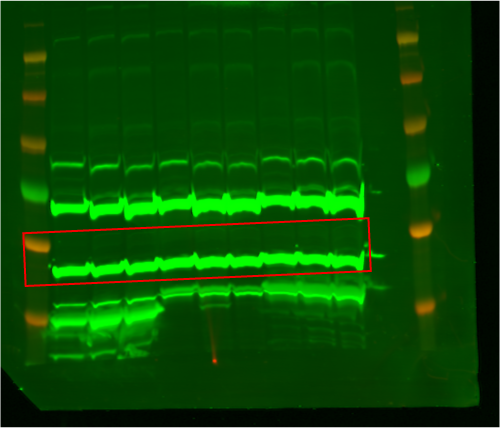

Supplement: Figure 3—source data 1. [file elife-90316-fig3-data1.zip › 3A/Figure 3A 100321-Actin-293T conNSP5C145A transfection 24 48 72h labeled.tif]

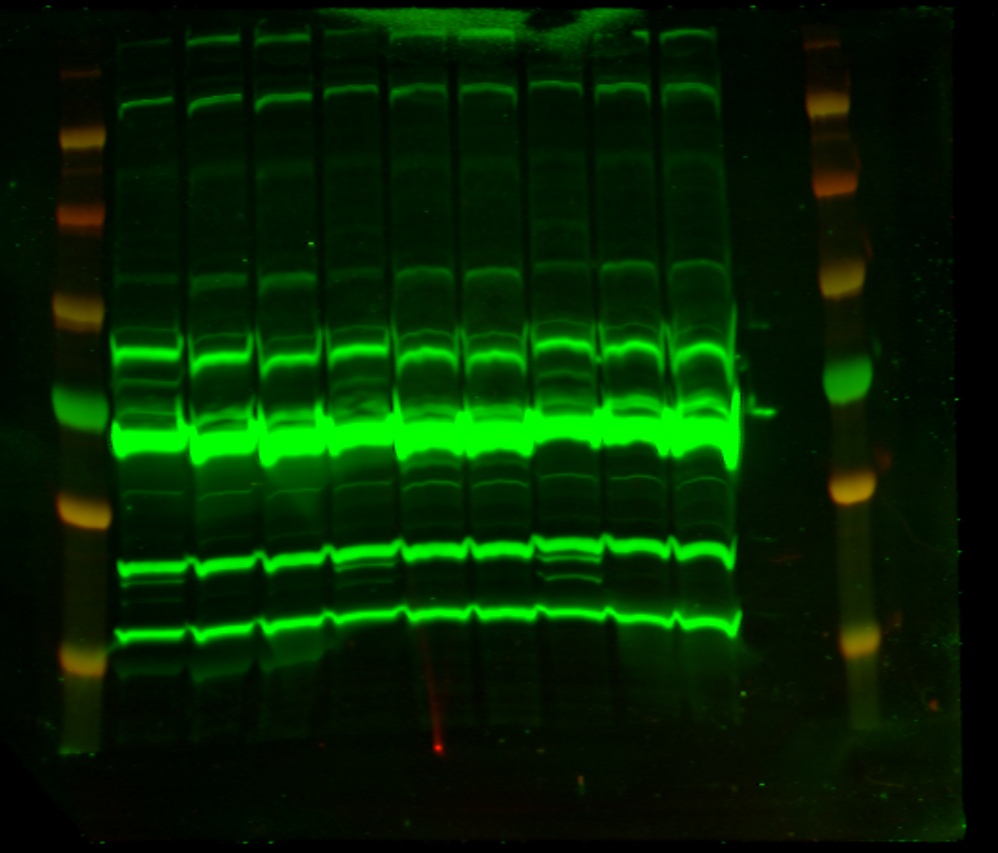

Supplement: Figure 3—source data 1. [file elife-90316-fig3-data1.zip › 3A/Figure 3A 100321-TRMT1G3-293T conNSP5C145A transfection 24 48 72h.tif]

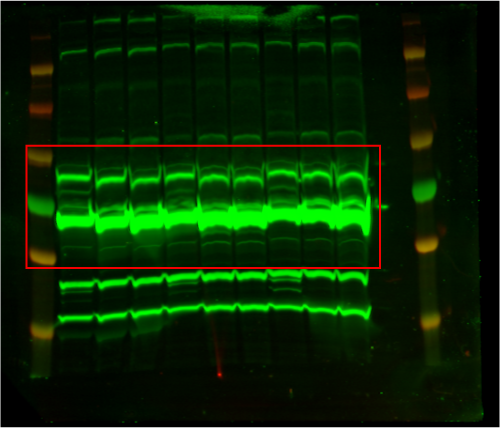

Supplement: Figure 3—source data 1. [file elife-90316-fig3-data1.zip › 3A/Figure 3A 100321-TRMT1G3-293T conNSP5C145A transfection 24 48 72h labeled.tif]

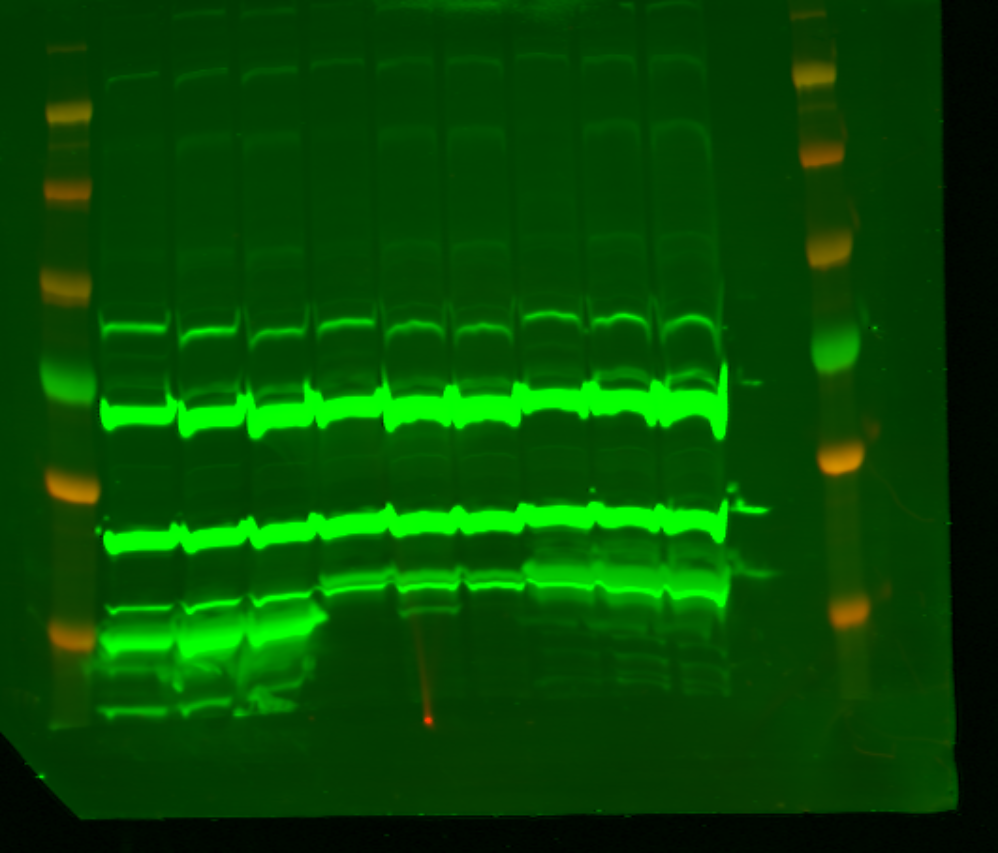

Supplement: Figure 3—source data 1. [file elife-90316-fig3-data1.zip › 3A/Figure 3A 100321-Strep-293T conNSP5C145A transfection 24 48 72h.tif]

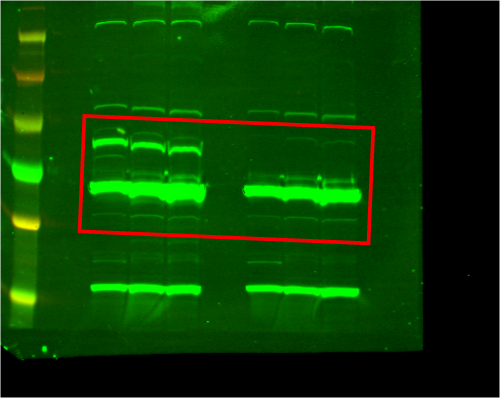

Supplement: Figure 3—source data 1. [file elife-90316-fig3-data1.zip › 3D/Figure 3D 052322-TRMT1G3-Scr TRMT1KO NSP5 24 48 72h labeled.tif]

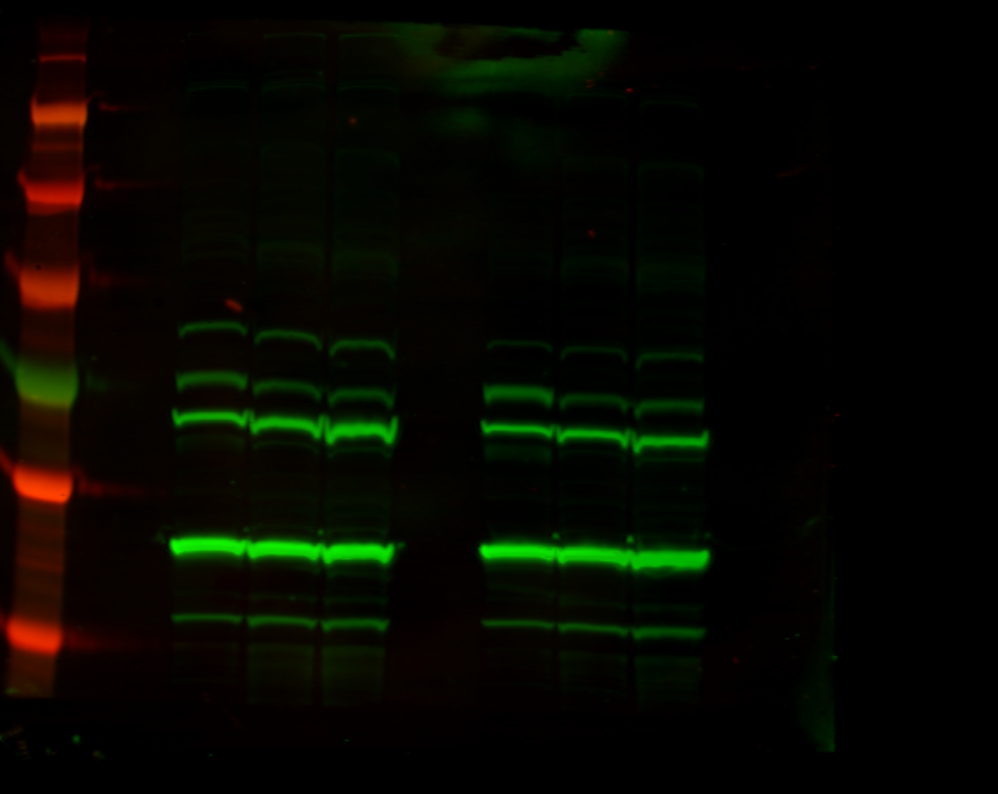

Supplement: Figure 3—source data 1. [file elife-90316-fig3-data1.zip › 3D/Figure 3D 20220526-actin-293T KO NSP5transfection.tif]

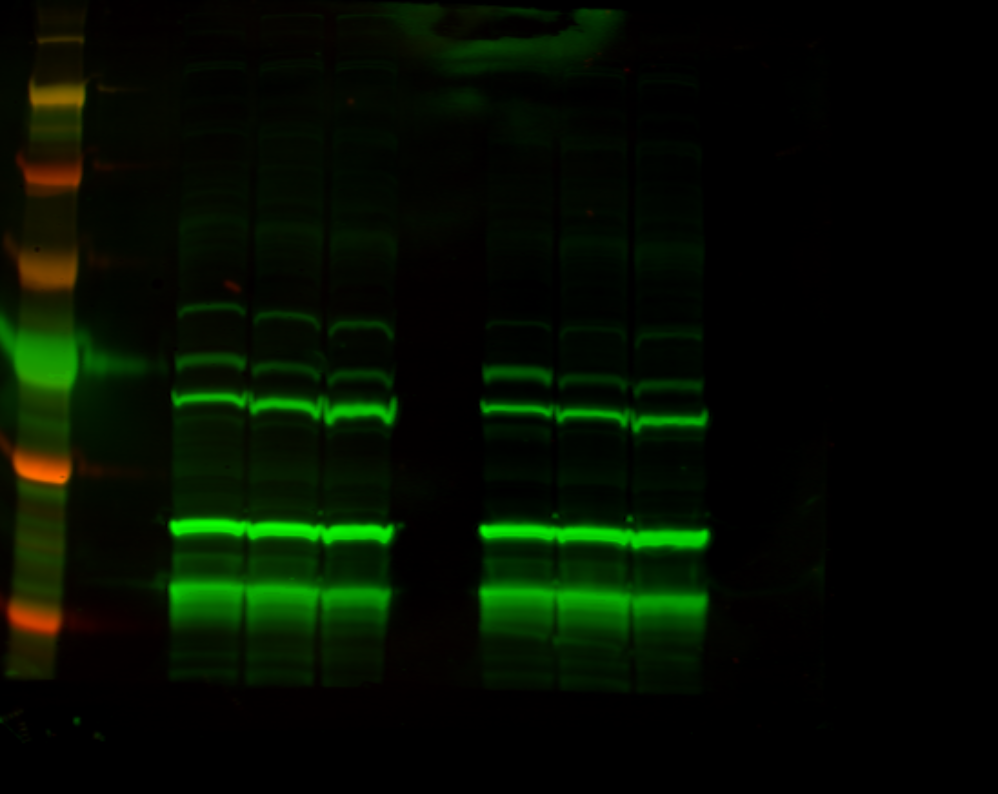

Supplement: Figure 3—source data 1. [file elife-90316-fig3-data1.zip › 3D/Figure 3D 20220527-Strep-293T KO NSP5transfection.tif]

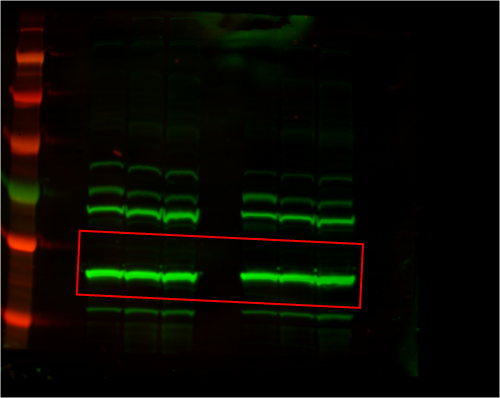

Supplement: Figure 3—source data 1. [file elife-90316-fig3-data1.zip › 3D/Figure 3D 20220526-actin-293T KO NSP5transfection labeled.tif]

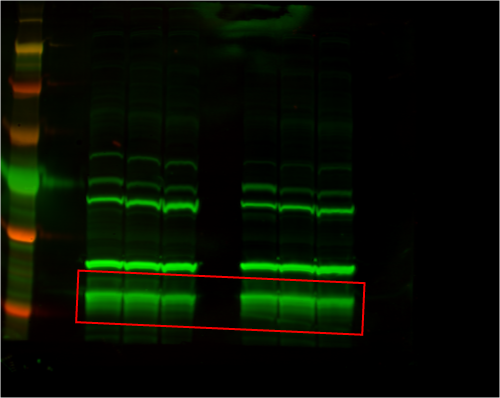

Supplement: Figure 3—source data 1. [file elife-90316-fig3-data1.zip › 3D/Figure 3D 20220527-Strep-293T KO NSP5transfection labeled.tif]

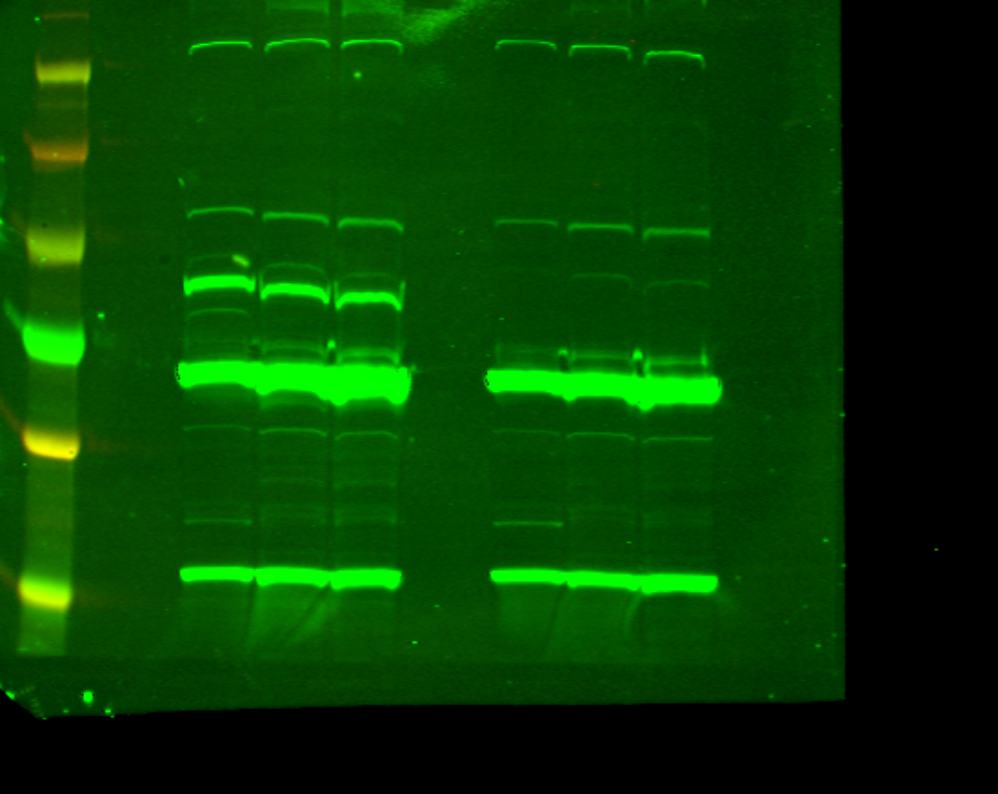

Supplement: Figure 3—source data 1. [file elife-90316-fig3-data1.zip › 3D/Figure 3D 052322-TRMT1G3-Scr TRMT1KO NSP5 24 48 72h.tif]

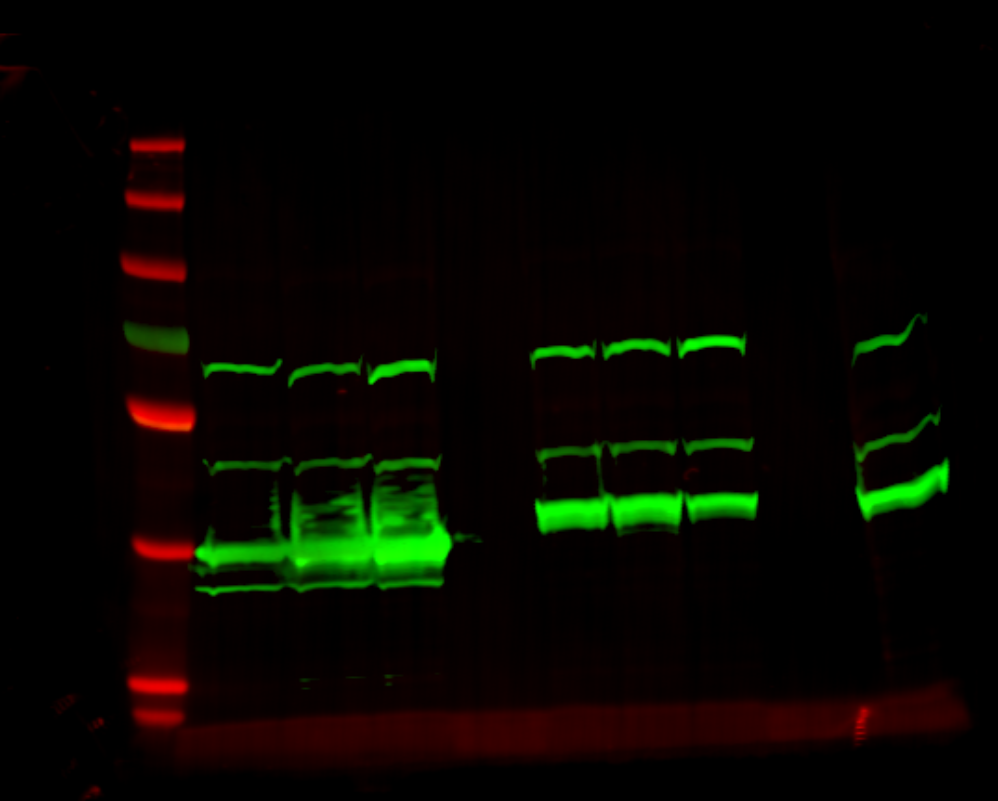

Supplement: Figure 3—source data 1. [file elife-90316-fig3-data1.zip › Rep1/091121-Actin-293T NSP5 transfection 24 48 72h.tif]

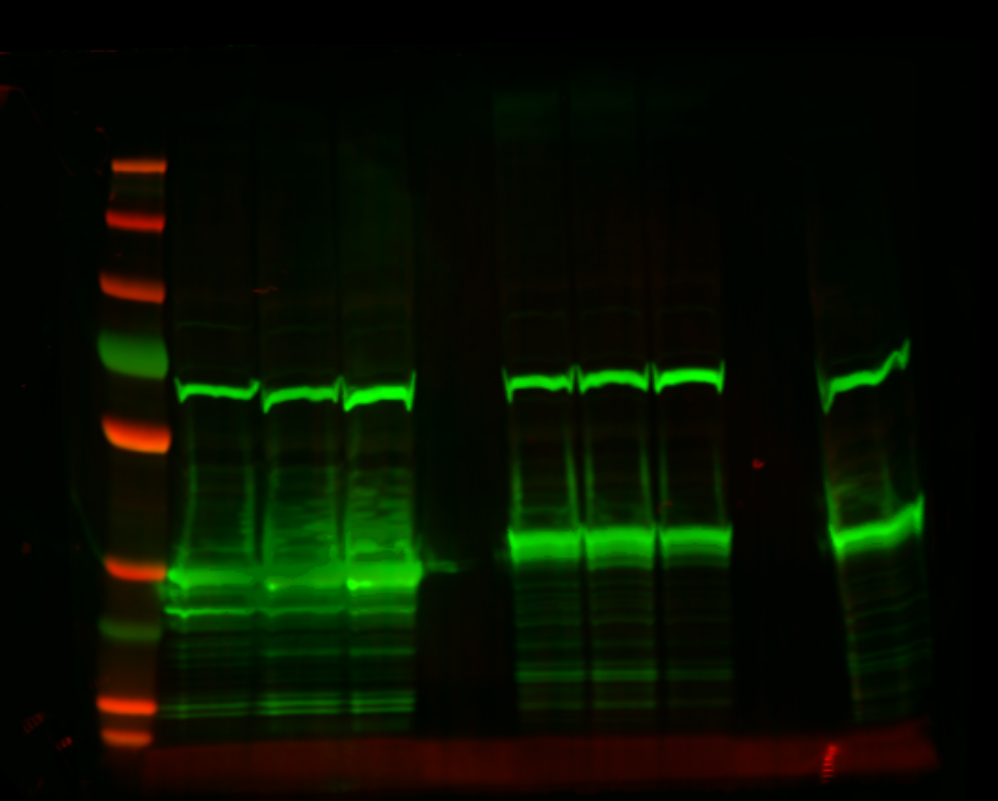

Supplement: Figure 3—source data 1. [file elife-90316-fig3-data1.zip › Rep1/091121-Strep-293T NSP5 transfection 24 48 72h.tif]

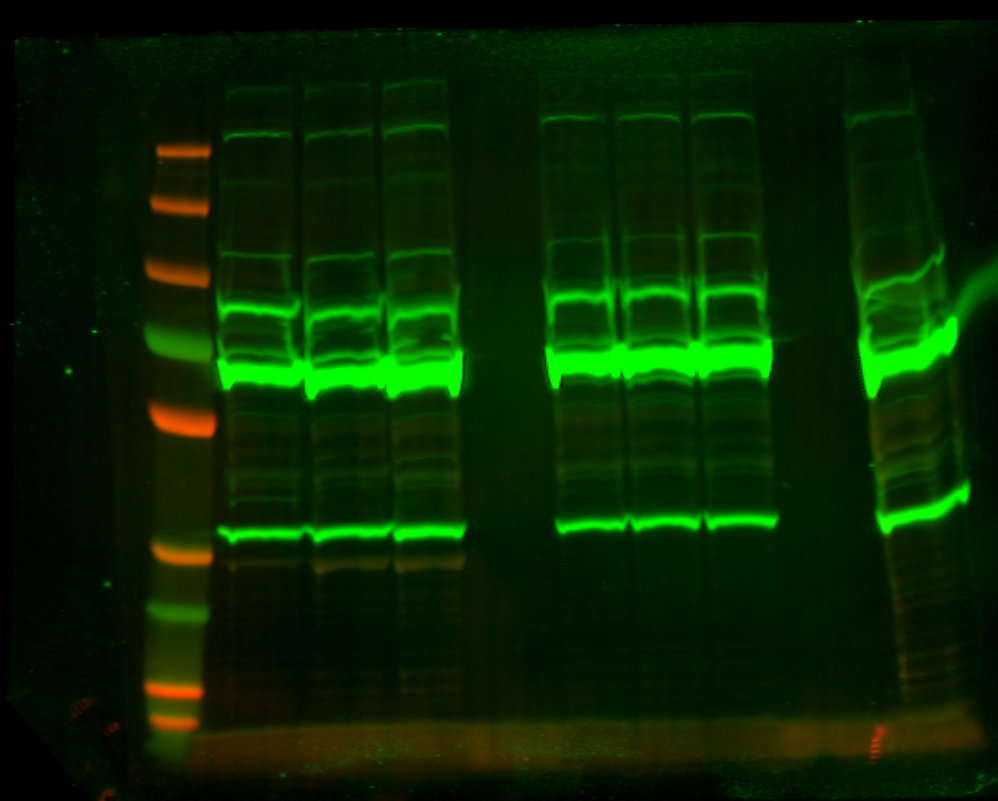

Supplement: Figure 3—source data 1. [file elife-90316-fig3-data1.zip › Rep1/091121-TRMT1G3-293T NSP5 transfection 24 48 72h.tif]

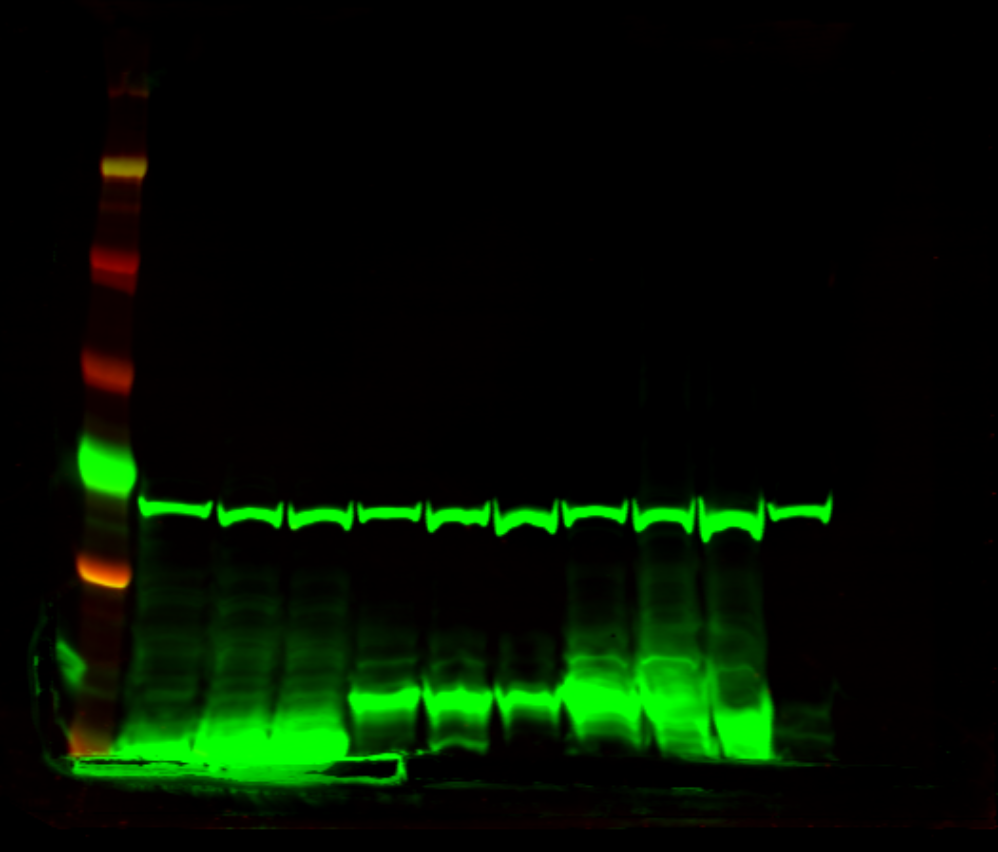

Supplement: Figure 3—source data 1. [file elife-90316-fig3-data1.zip › Rep2/100721-Strep-293T conNSP5C145A transfection 24 48 72h KO.tif]

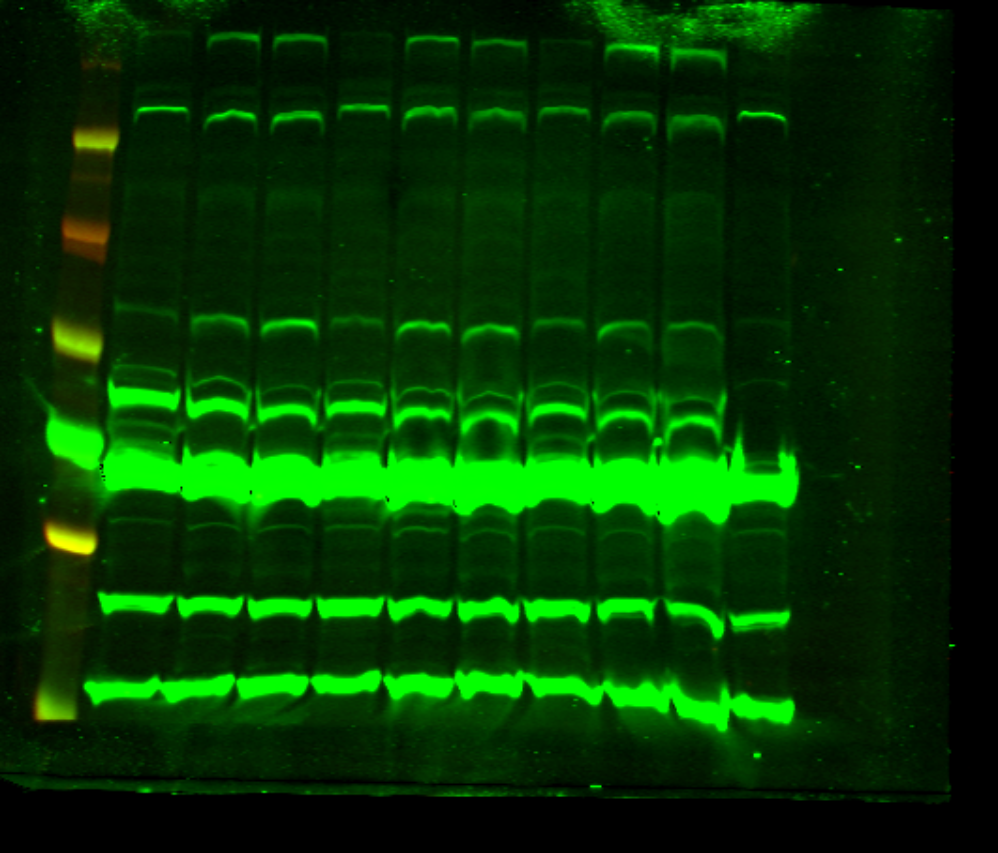

Supplement: Figure 3—source data 1. [file elife-90316-fig3-data1.zip › Rep2/100721-TRMT1G3-293T conNSP5C145A transfection 24 48 72h KO.tif]

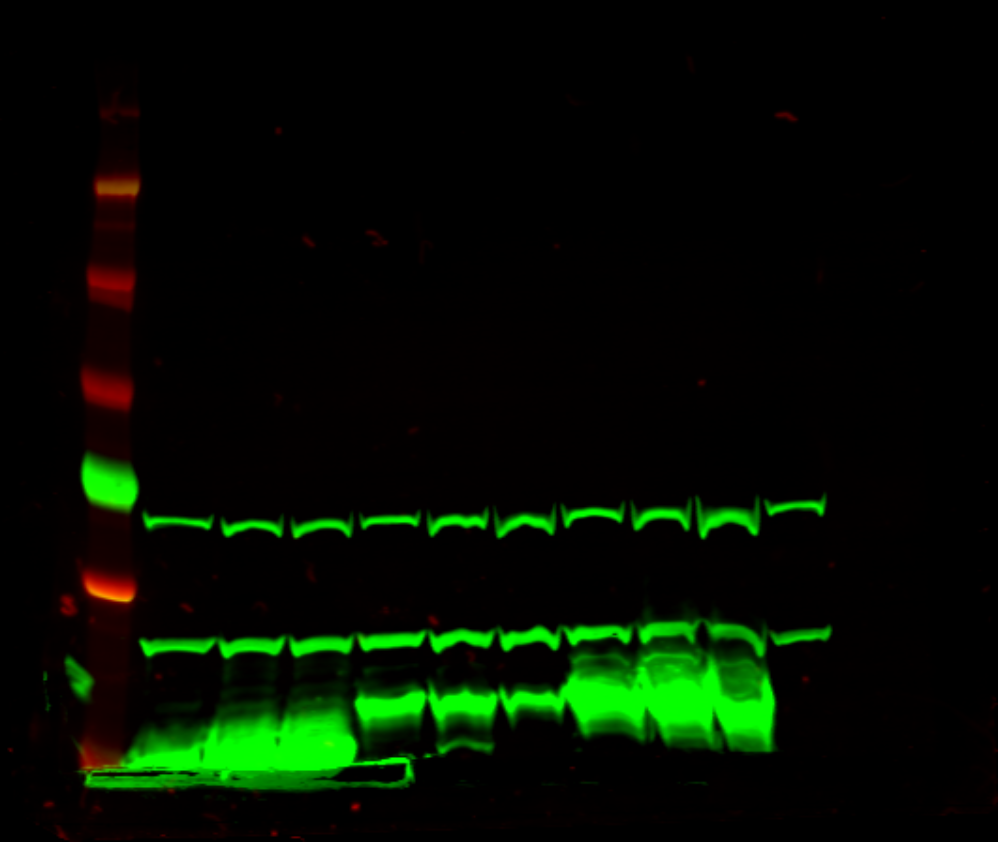

Supplement: Figure 3—source data 1. [file elife-90316-fig3-data1.zip › Rep2/100721-Actin-293T conNSP5C145A transfection 24 48 72h KO.tif.tif]

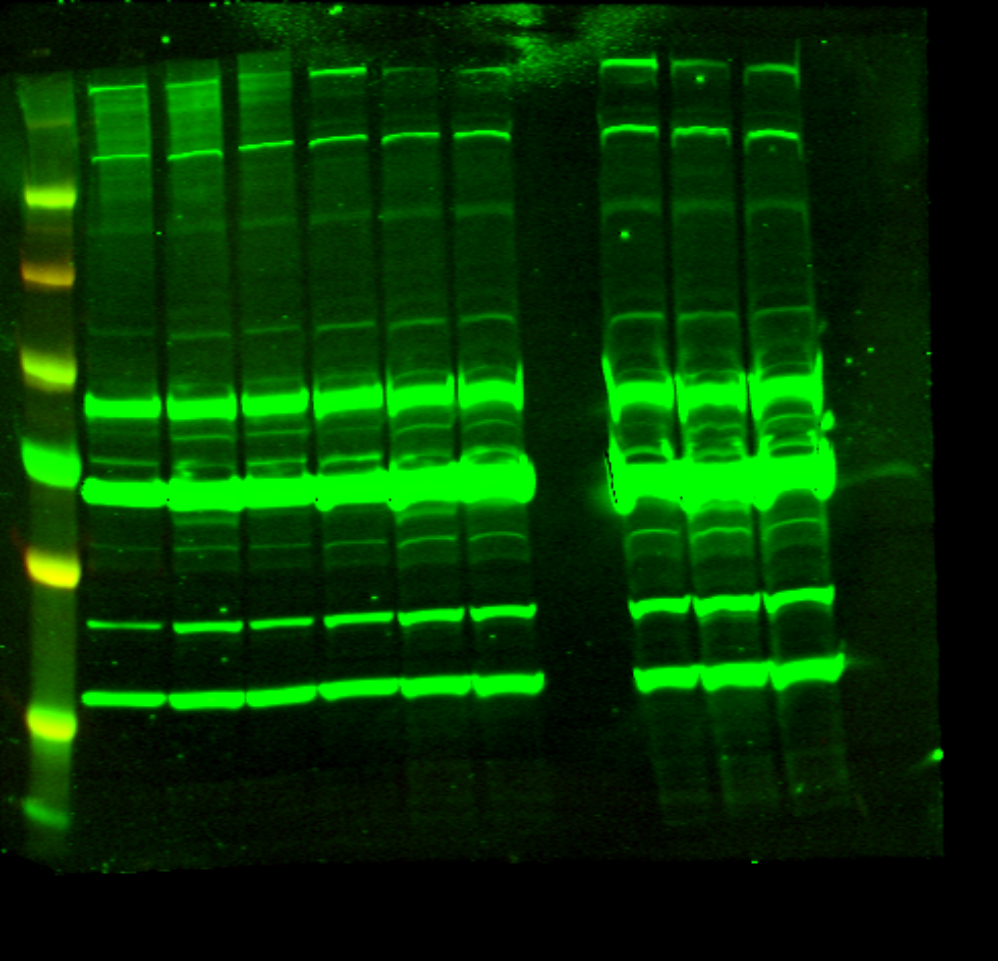

Supplement: Figure 3—source data 1. [file elife-90316-fig3-data1.zip › Rep3/102521-TRMT1G3-293T conNSP5C145A transfection 48h 5 1020uL load.tif.tif]

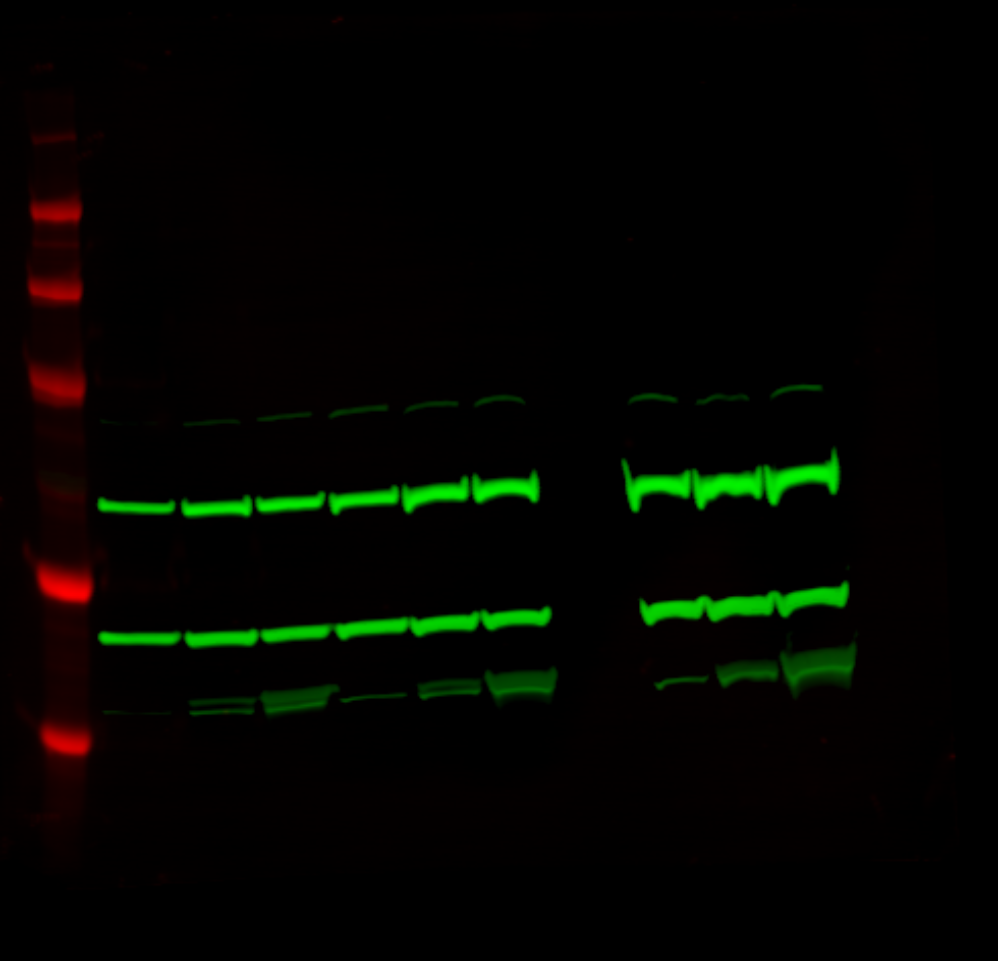

Supplement: Figure 3—source data 1. [file elife-90316-fig3-data1.zip › Rep3/102521-Actin-293T conNSP5C145A transfection 48h 5 1020uL load.tif.tif]

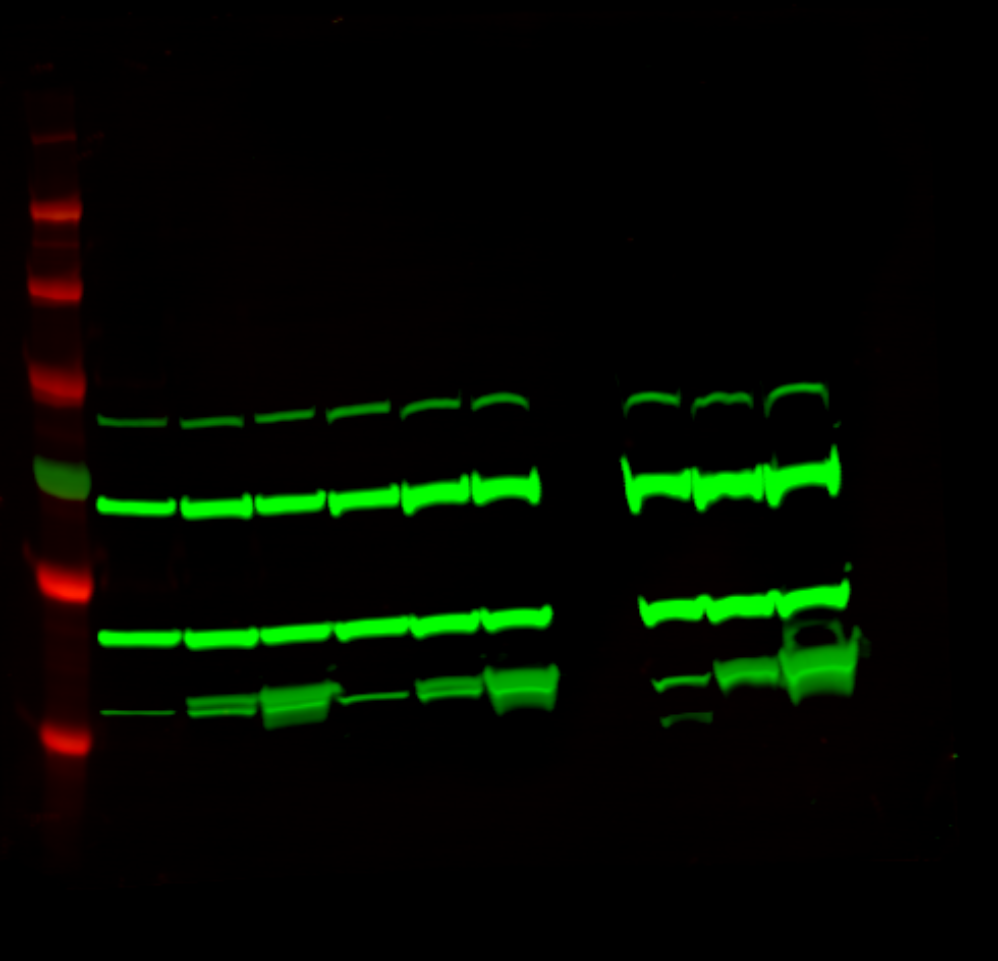

Supplement: Figure 3—source data 1. [file elife-90316-fig3-data1.zip › Rep3/102521-Strep-293T conNSP5C145A transfection 48h 5 1020uL load.tif.tif]

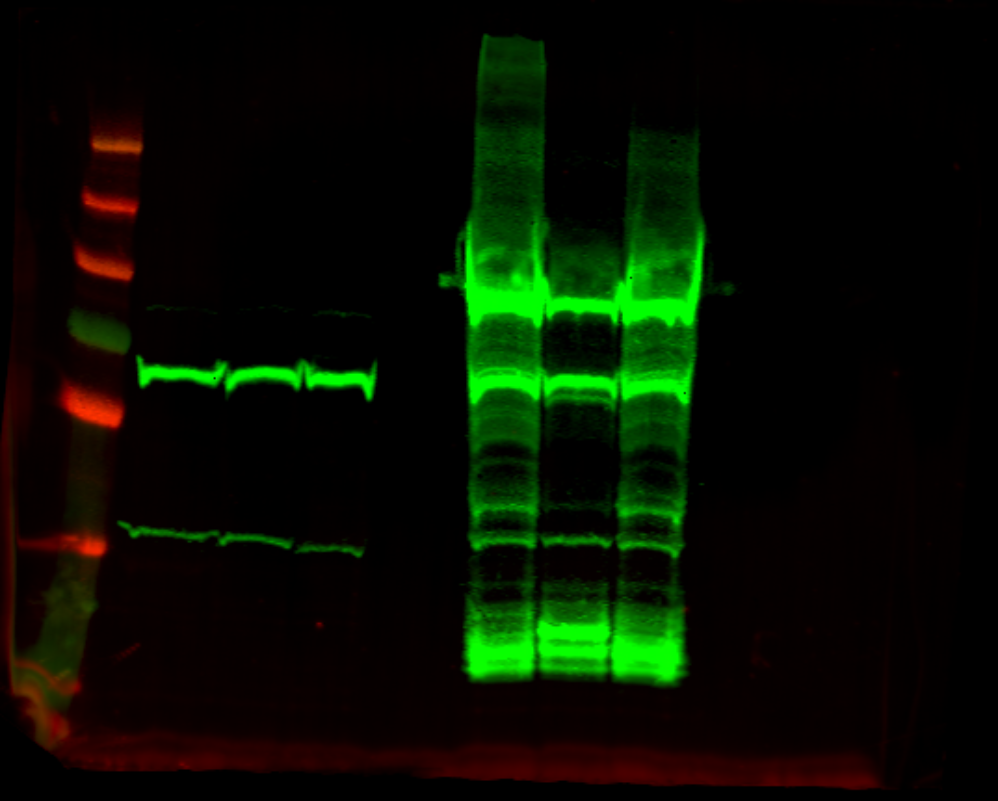

Supplement: Figure 4—source data 1. [file elife-90316-fig4-data1.zip › 4A/Rep2/061721-TRMT1G3-293T TRMT1flag conNSP5C145A transfection.tif.tif]

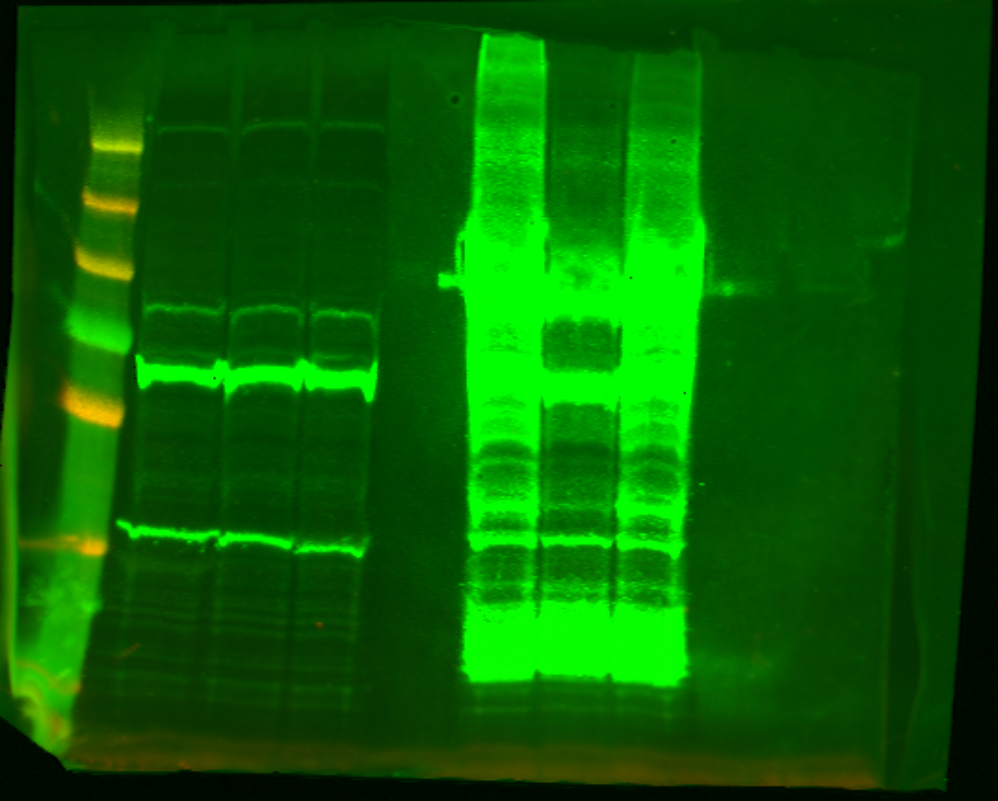

Supplement: Figure 4—source data 1. [file elife-90316-fig4-data1.zip › 4A/Rep2/061721-TRMT1G3-293T TRMT1flag conNSP5C145A transfection- overexposure.tif.png]

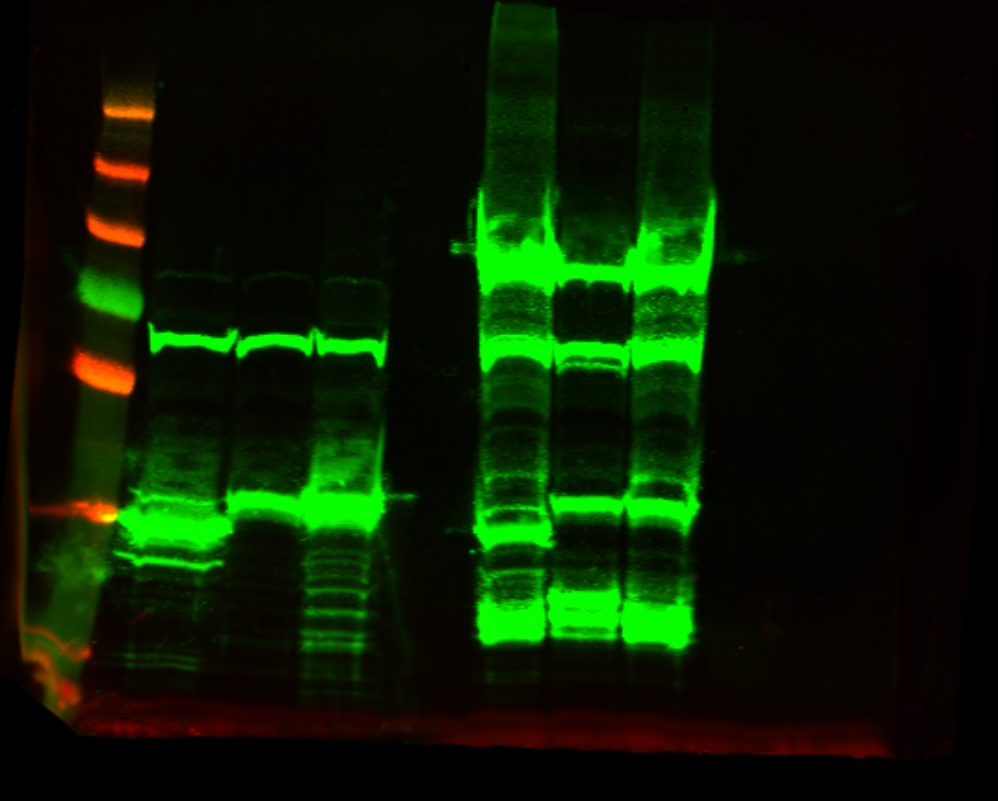

Supplement: Figure 4—source data 1. [file elife-90316-fig4-data1.zip › 4A/Rep2/061721-Strep-293T TRMT1flag conNSP5C145A transfection.tif.tif]

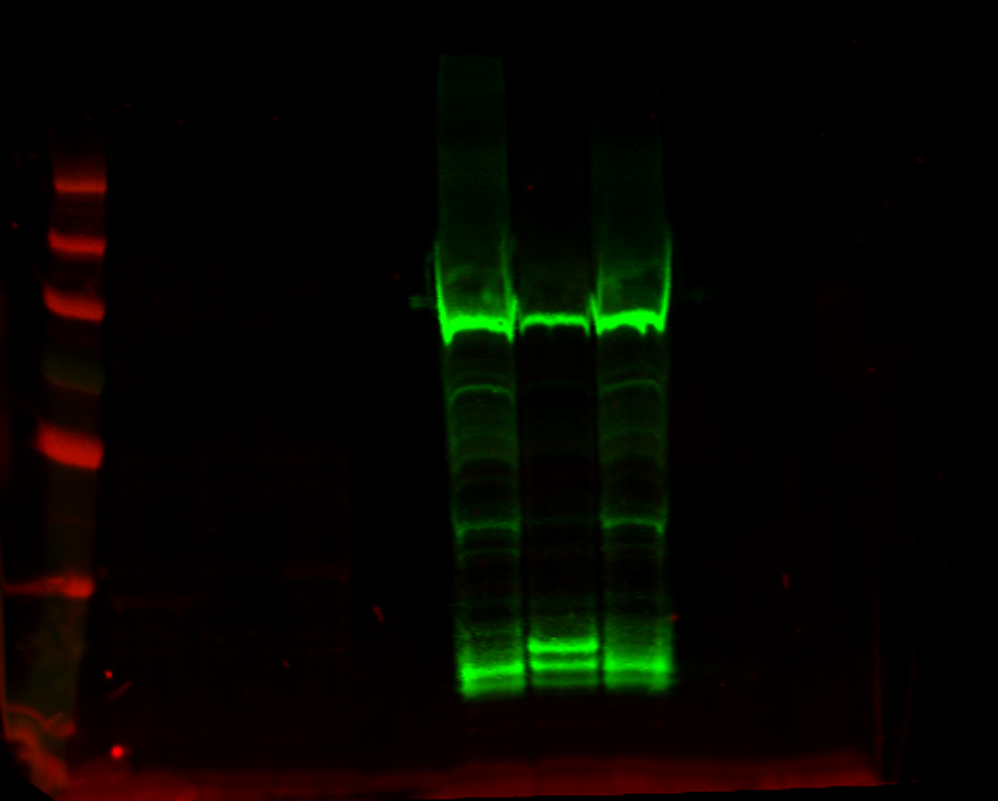

Supplement: Figure 4—source data 1. [file elife-90316-fig4-data1.zip › 4A/Rep2/061721-Flag-293T TRMT1flag conNSP5C145A transfection.tif.tif]

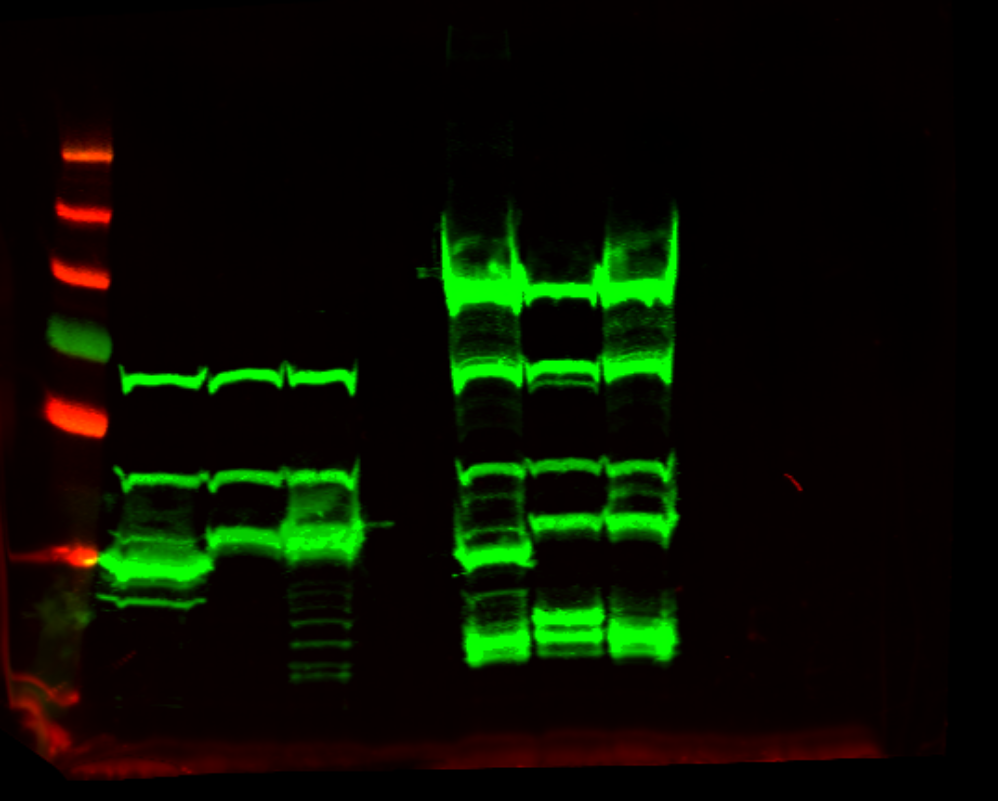

Supplement: Figure 4—source data 1. [file elife-90316-fig4-data1.zip › 4A/Rep2/061721-Actin-293T TRMT1flag conNSP5C145A transfection.tif.tif]

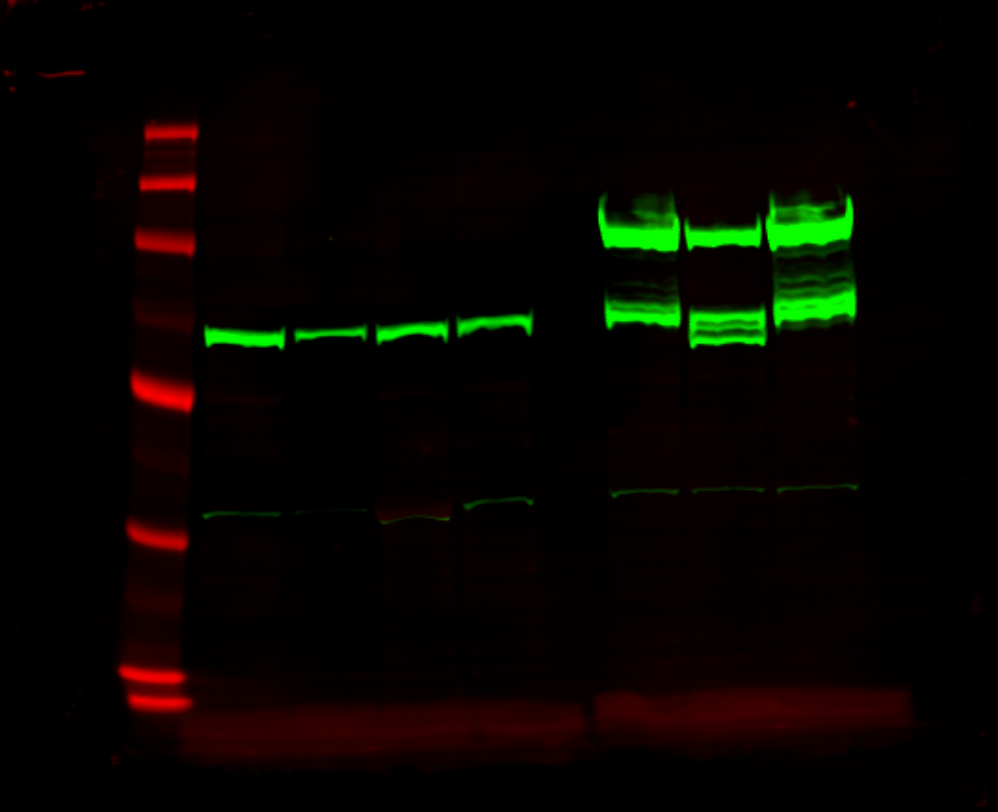

Supplement: Figure 4—source data 1. [file elife-90316-fig4-data1.zip › 4A/Rep1/Figure 4A 060820-TRMT1G3-293T TRMT1flag conNSP5C145A transfection.tif]

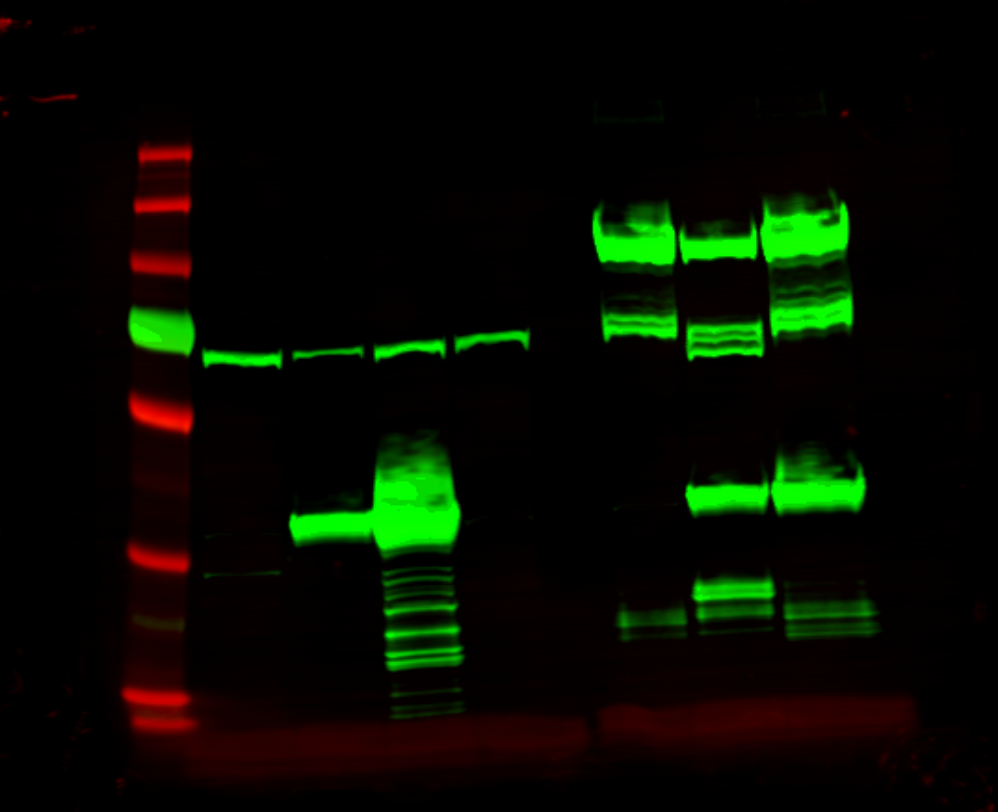

Supplement: Figure 4—source data 1. [file elife-90316-fig4-data1.zip › 4A/Rep1/Figure 4A 060820-flag-293T TRMT1flag conNSP5C145A transfection.tif]

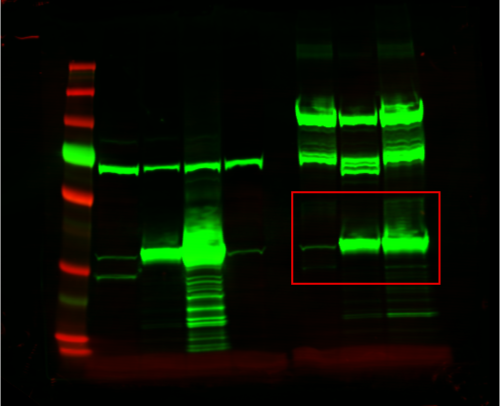

Supplement: Figure 4—source data 1. [file elife-90316-fig4-data1.zip › 4A/Rep1/Figure 4A 060820-Strep-293T TRMT1flag conNSP5C145A transfection labeled.tif]

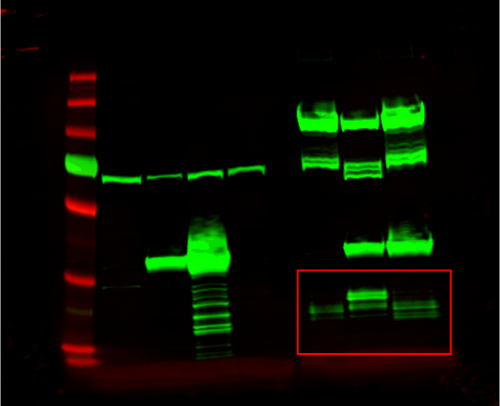

Supplement: Figure 4—source data 1. [file elife-90316-fig4-data1.zip › 4A/Rep1/Figure 4A 060820-flag-293T TRMT1flag conNSP5C145A transfection labeled.tif]

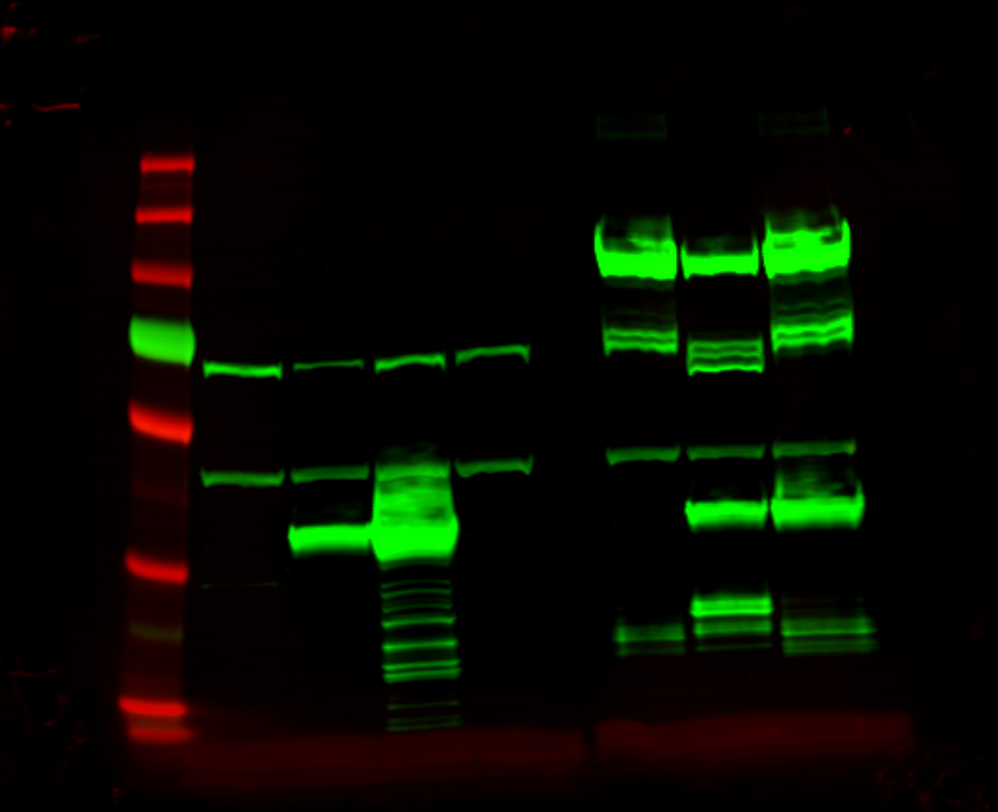

Supplement: Figure 4—source data 1. [file elife-90316-fig4-data1.zip › 4A/Rep1/Figure 4A 060820-Actin-293T TRMT1flag conNSP5C145A transfection.tif]

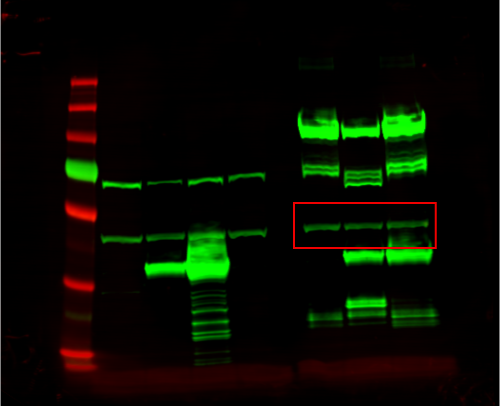

Supplement: Figure 4—source data 1. [file elife-90316-fig4-data1.zip › 4A/Rep1/Figure 4A 060820-Actin-293T TRMT1flag conNSP5C145A transfection labeled.tif]

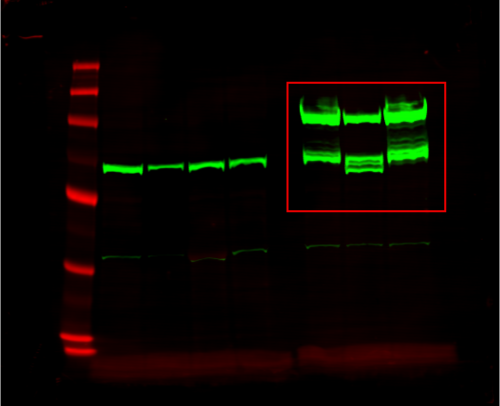

Supplement: Figure 4—source data 1. [file elife-90316-fig4-data1.zip › 4A/Rep1/Figure 4A 060820-TRMT1G3-293T TRMT1flag conNSP5C145A transfection labeled.tif]

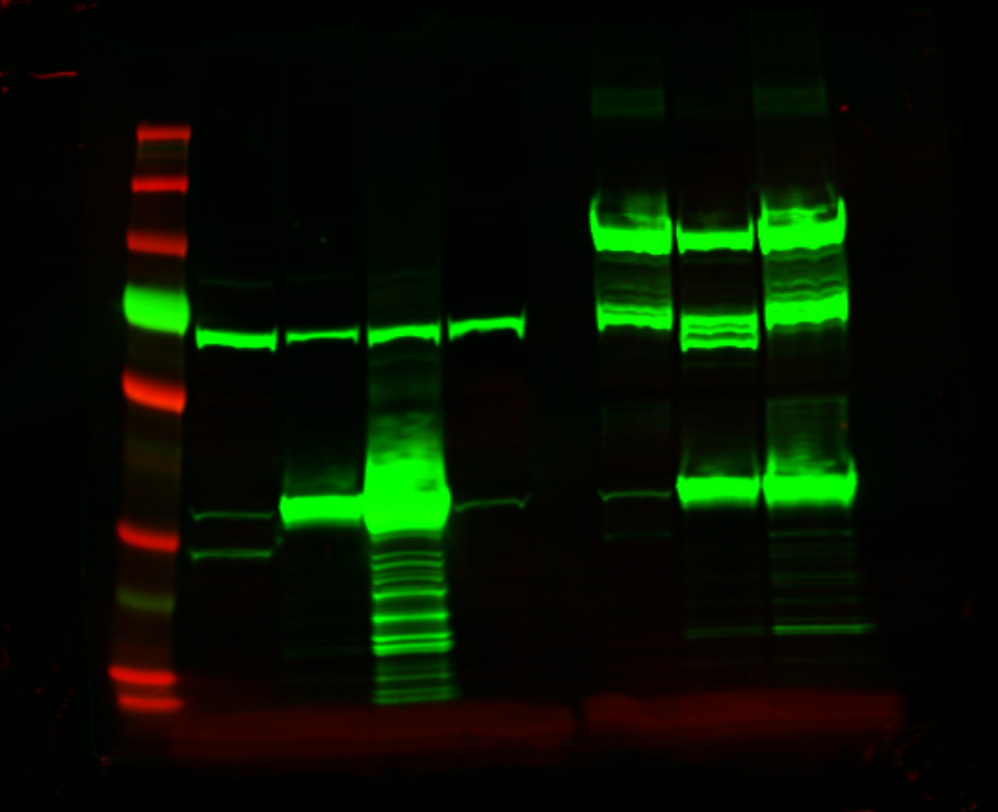

Supplement: Figure 4—source data 1. [file elife-90316-fig4-data1.zip › 4A/Rep1/Figure 4A 060820-Strep-293T TRMT1flag conNSP5C145A transfection.tif]

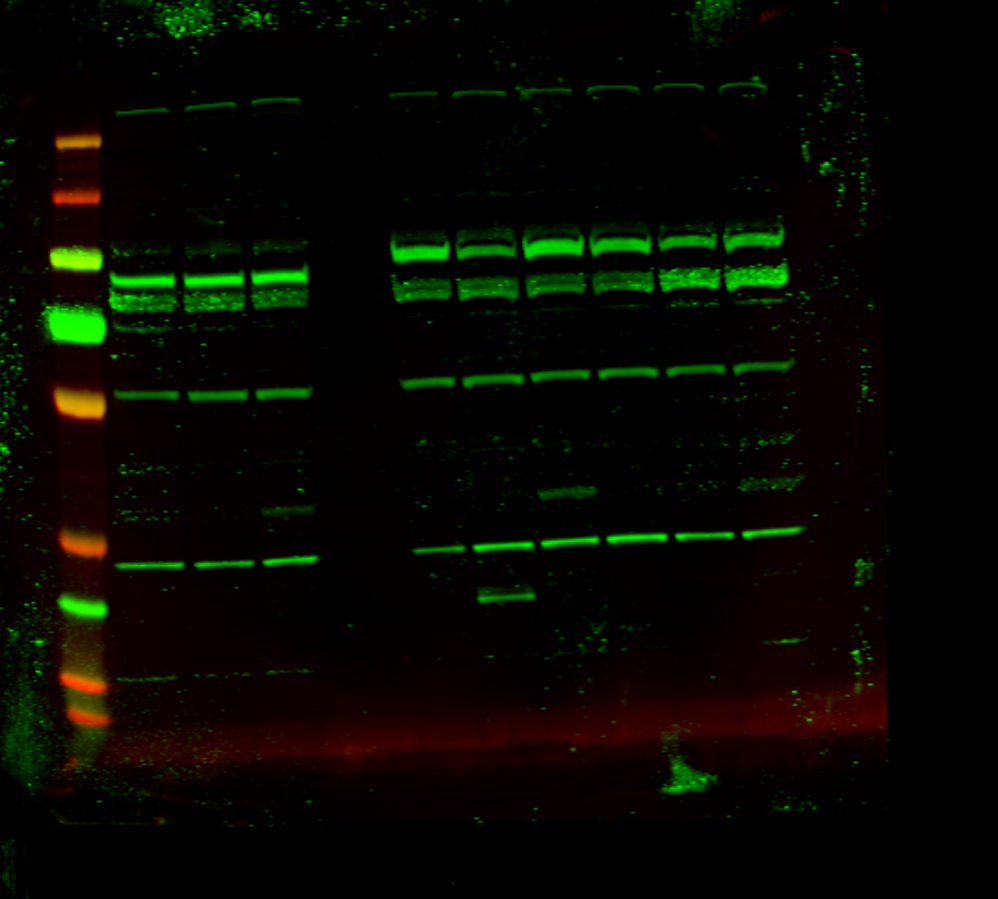

Supplement: Figure 4—source data 1. [file elife-90316-fig4-data1.zip › 4C/Exp 3/112021-TRMT1Cterm-293T TRMT1flag Q530NTRMT1flag conNSP5C145A transfection.tif.tif]

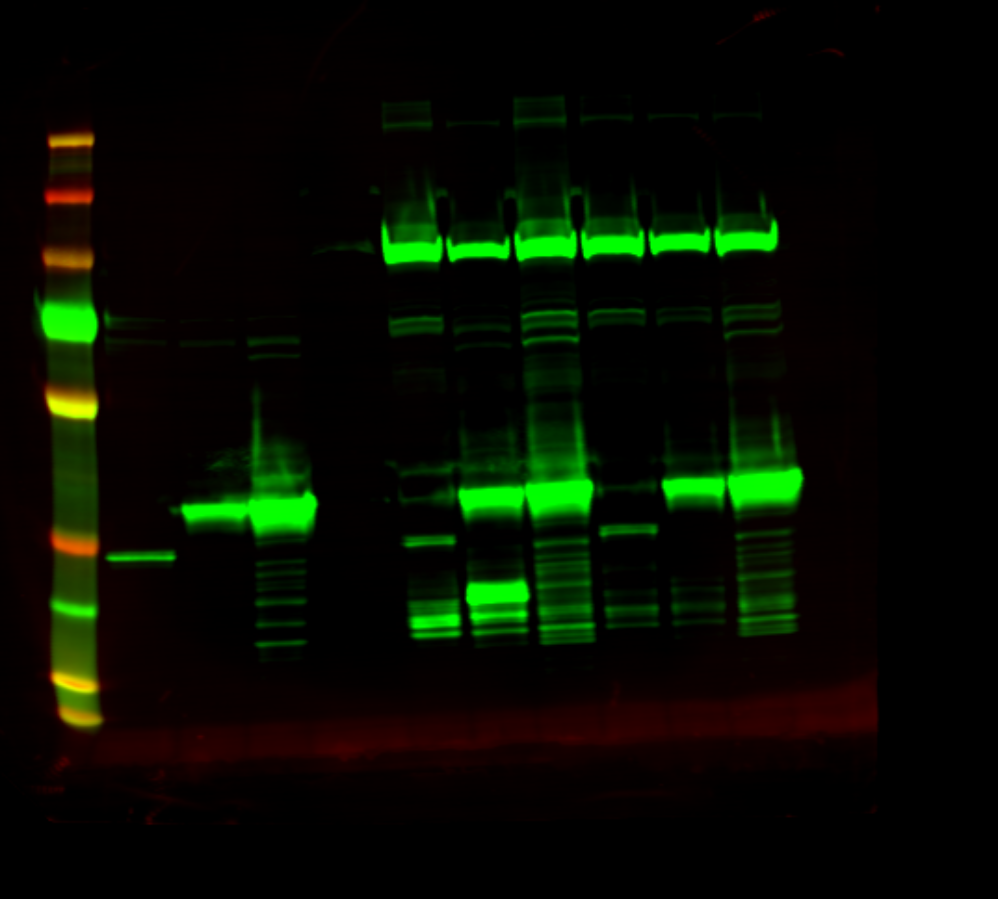

Supplement: Figure 4—source data 1. [file elife-90316-fig4-data1.zip › 4C/Exp 3/112021-strep-293T TRMT1flag Q530NTRMT1flag conNSP5C145A transfection.tif.tif]

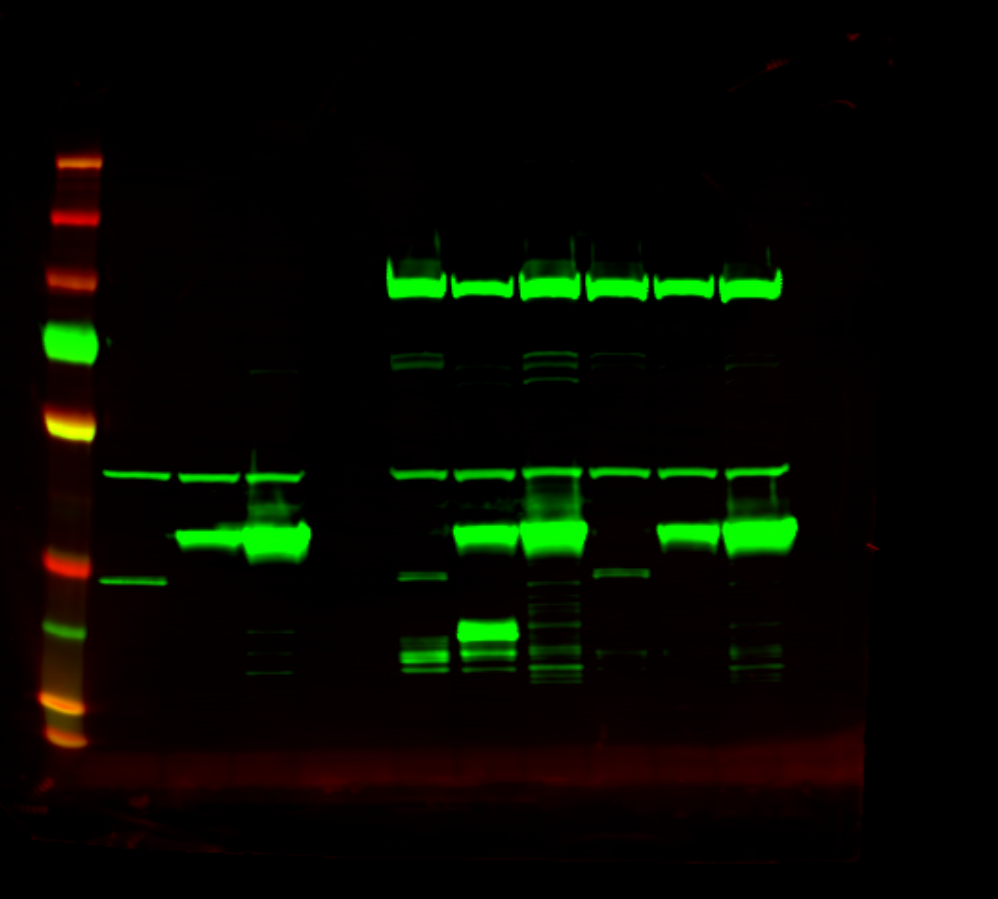

Supplement: Figure 4—source data 1. [file elife-90316-fig4-data1.zip › 4C/Exp 3/112021-actin-293T TRMT1flag Q530NTRMT1flag conNSP5C145A transfection.tif.tif]

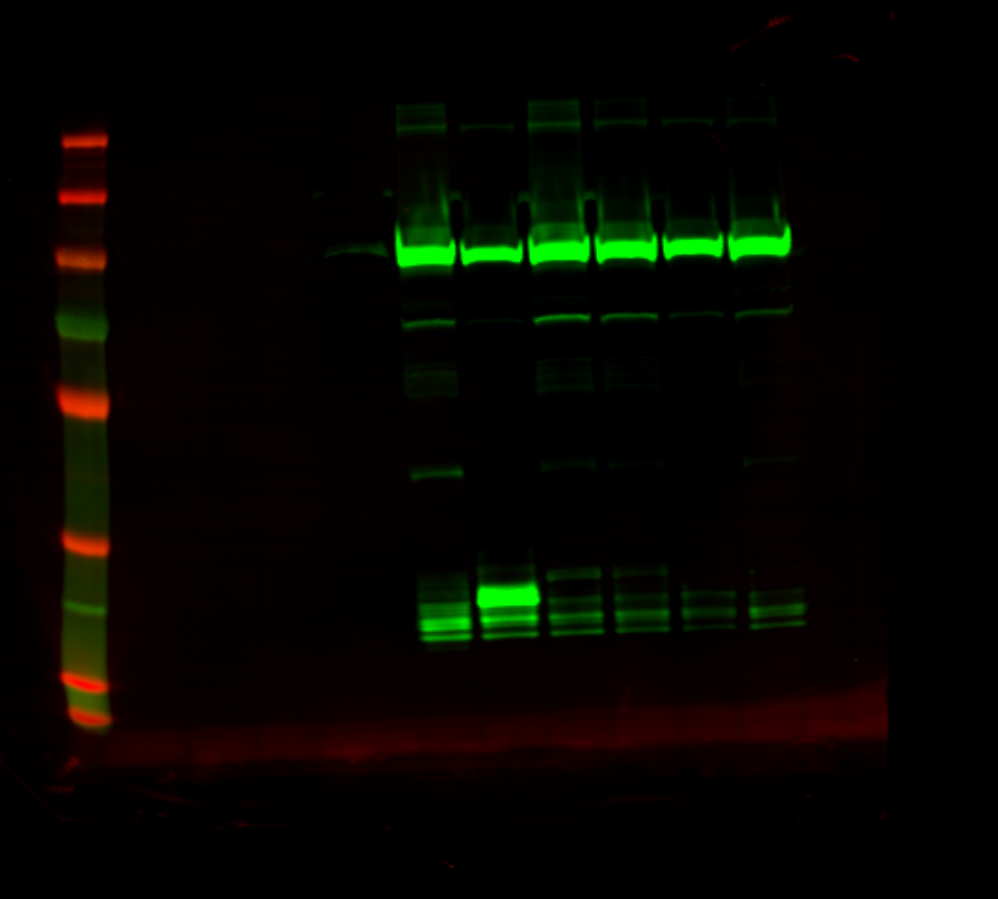

Supplement: Figure 4—source data 1. [file elife-90316-fig4-data1.zip › 4C/Exp 3/112021-flag-293T TRMT1flag Q530NTRMT1flag conNSP5C145A transfection.tif.tif]

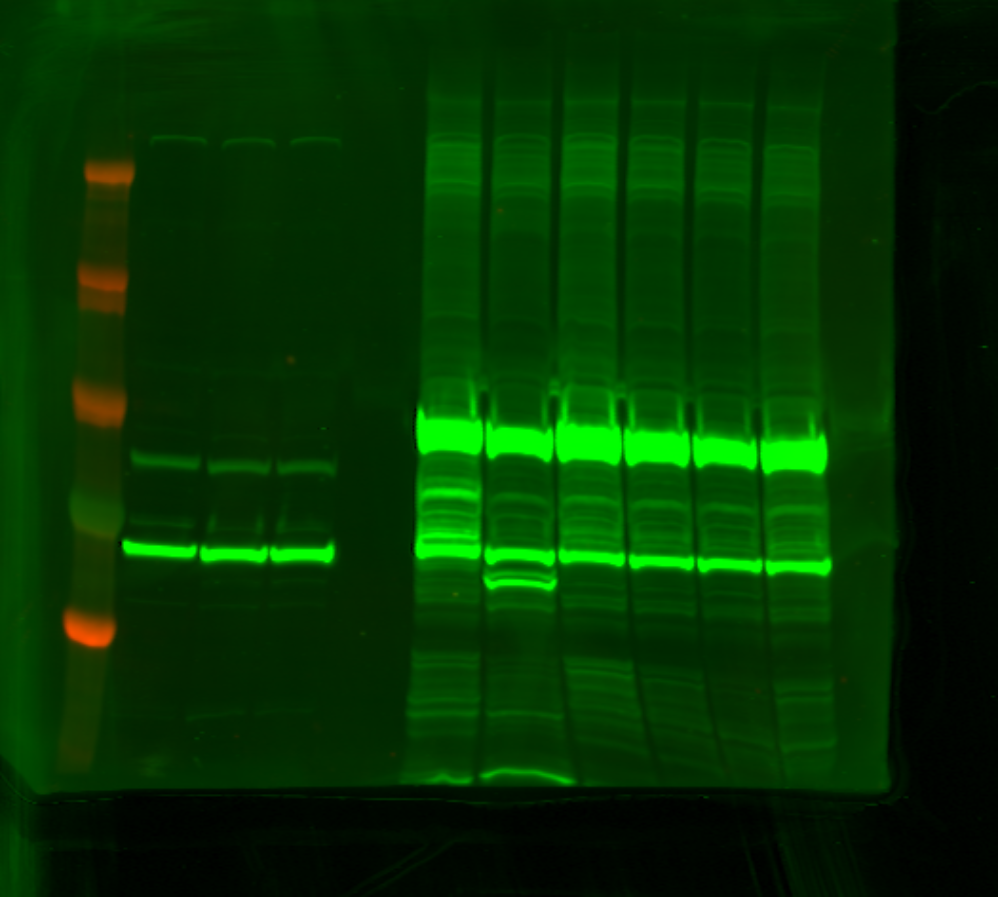

Supplement: Figure 4—source data 1. [file elife-90316-fig4-data1.zip › 4C/Exp 2/111821-TRMT1G3-293T TRMT1flag Q530NTRMT1flag conNSP5C145A transfection.tif.tif]

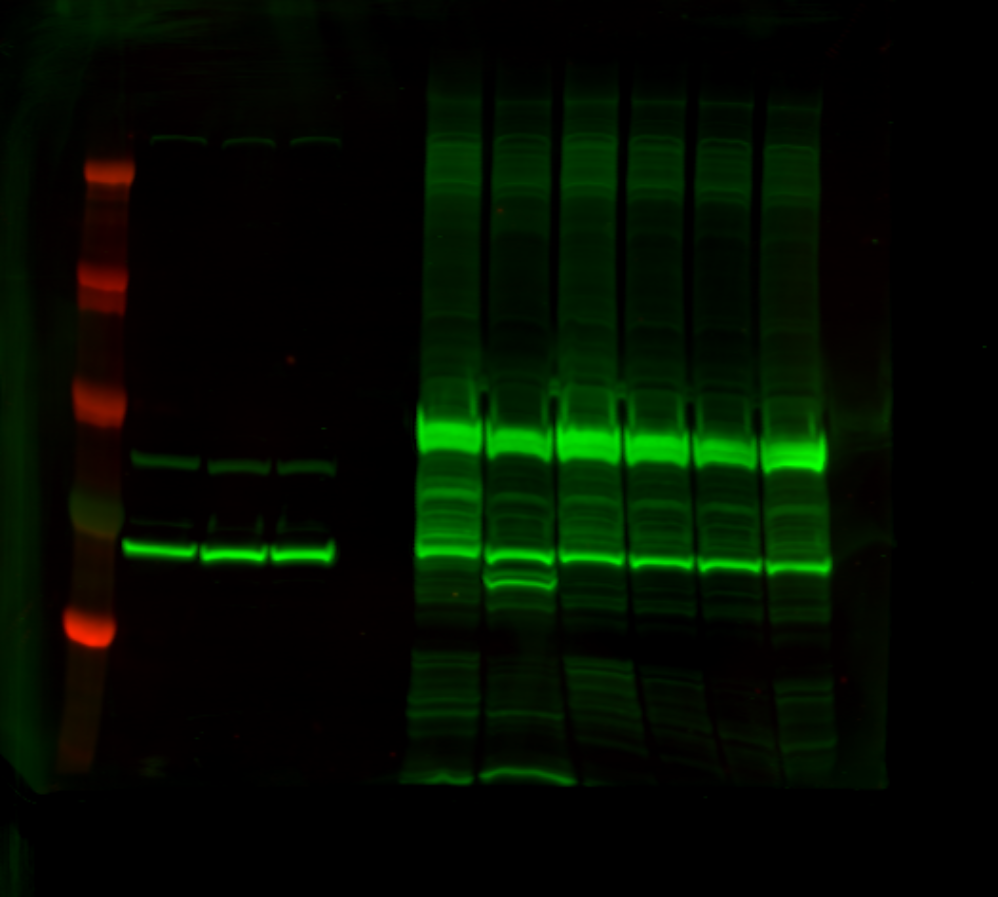

Supplement: Figure 4—source data 1. [file elife-90316-fig4-data1.zip › 4C/Exp 2/111821-TRMT1G3-293T TRMT1flag Q530NTRMT1flag conNSP5C145A transfection-less exposure.tif.tif]

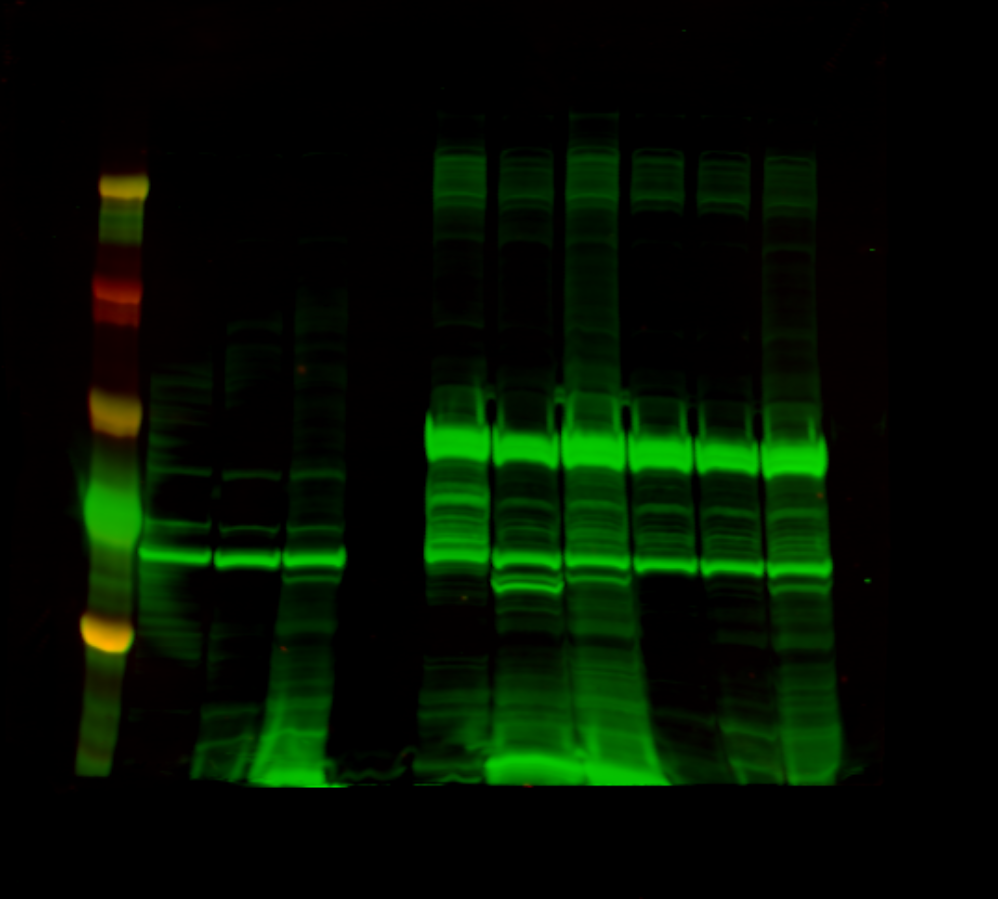

Supplement: Figure 4—source data 1. [file elife-90316-fig4-data1.zip › 4C/Exp 2/111821-Strep-293T TRMT1flag Q530NTRMT1flag conNSP5C145A transfection.tif.tif]

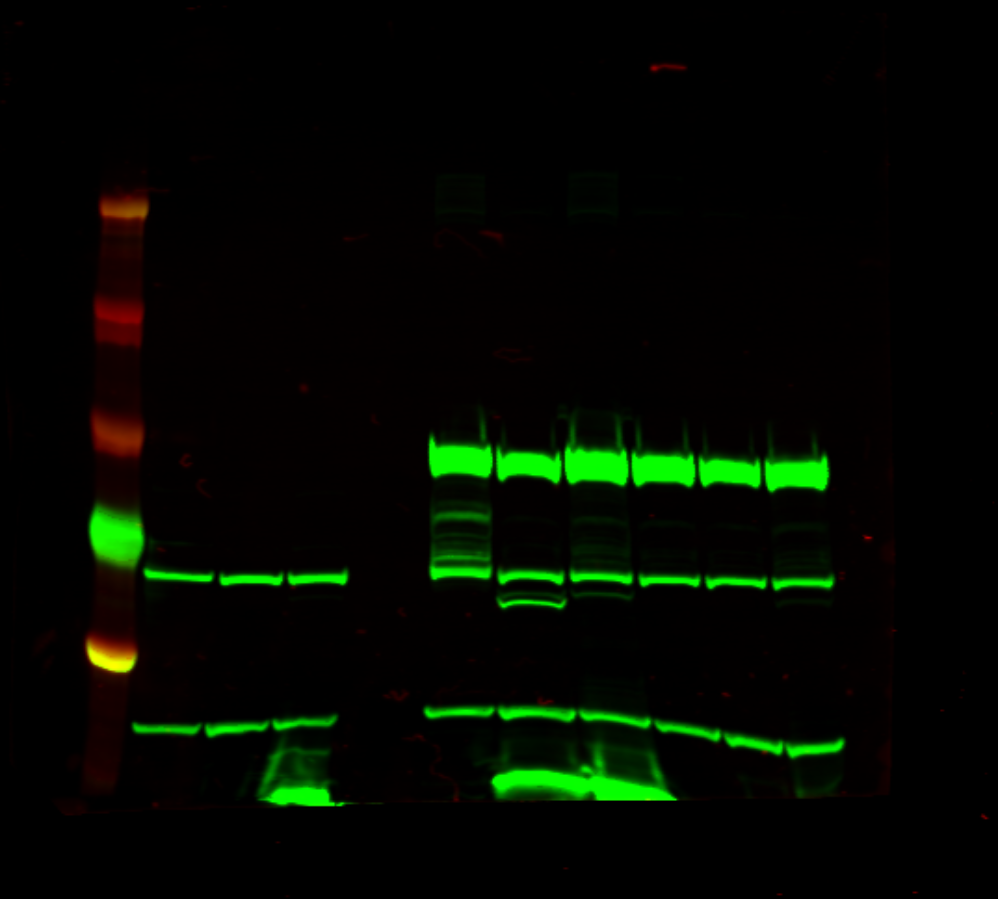

Supplement: Figure 4—source data 1. [file elife-90316-fig4-data1.zip › 4C/Exp 2/111821-actin-293T TRMT1flag Q530NTRMT1flag conNSP5C145A transfection.tif.tif]

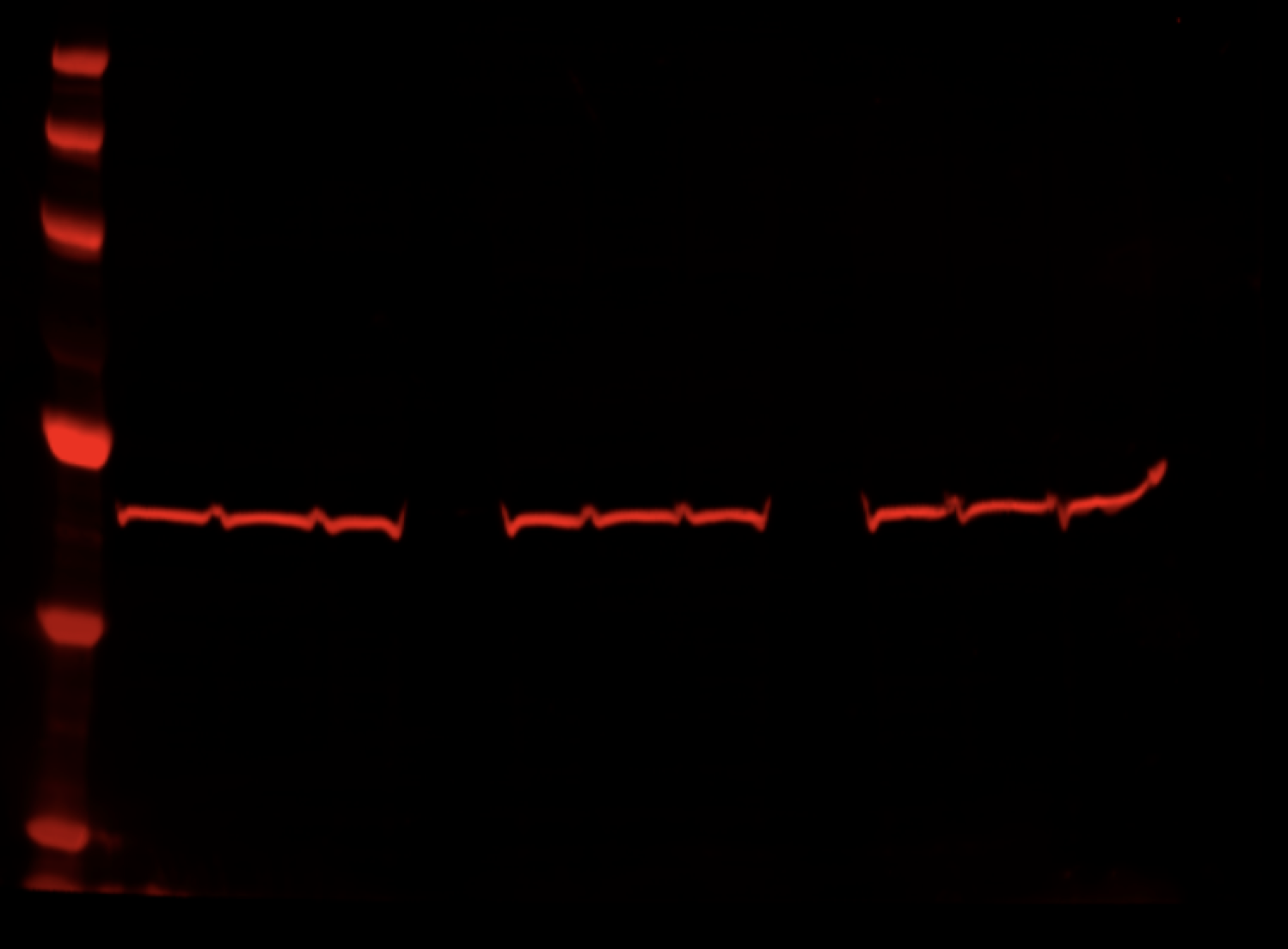

Supplement: Figure 4—source data 1. [file elife-90316-fig4-data1.zip › 4C/Exp 1/Figure 4C anti-actin Q530N.tif]

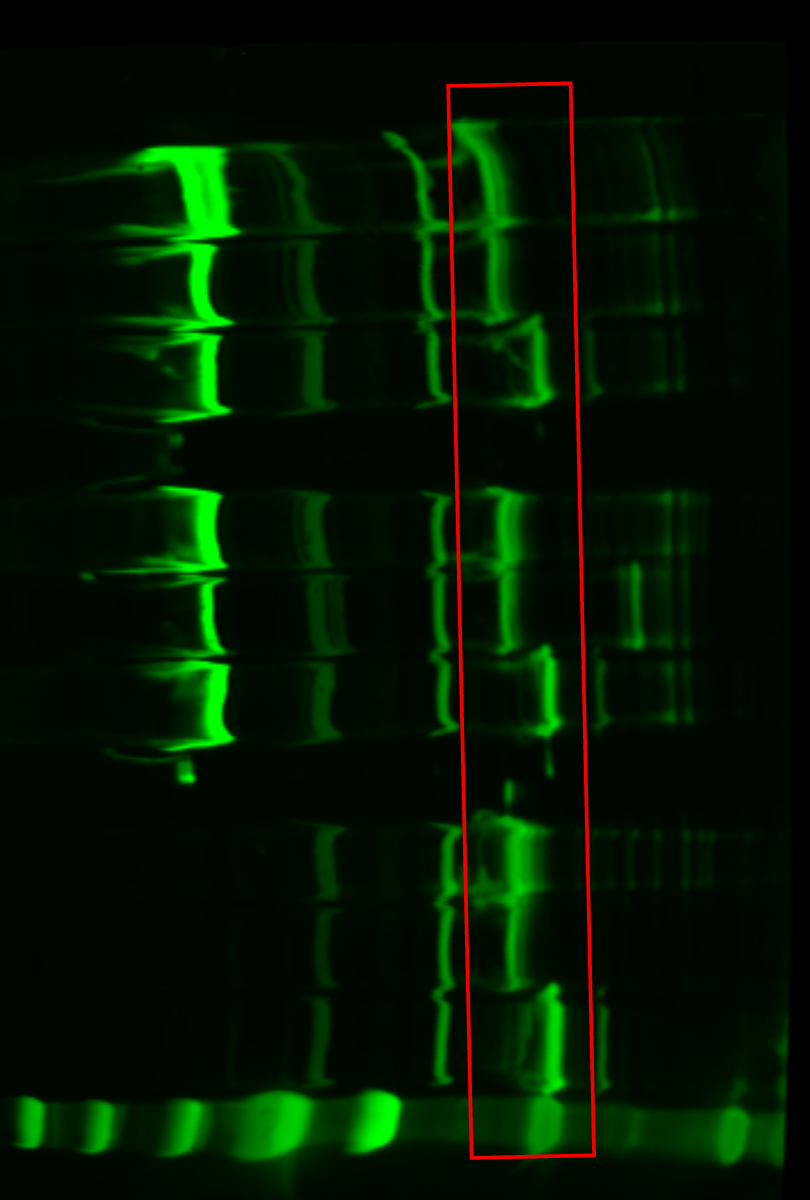

Supplement: Figure 4—source data 1. [file elife-90316-fig4-data1.zip › 4C/Exp 1/Figure 4C anti-strep Q530N labeled.tif]

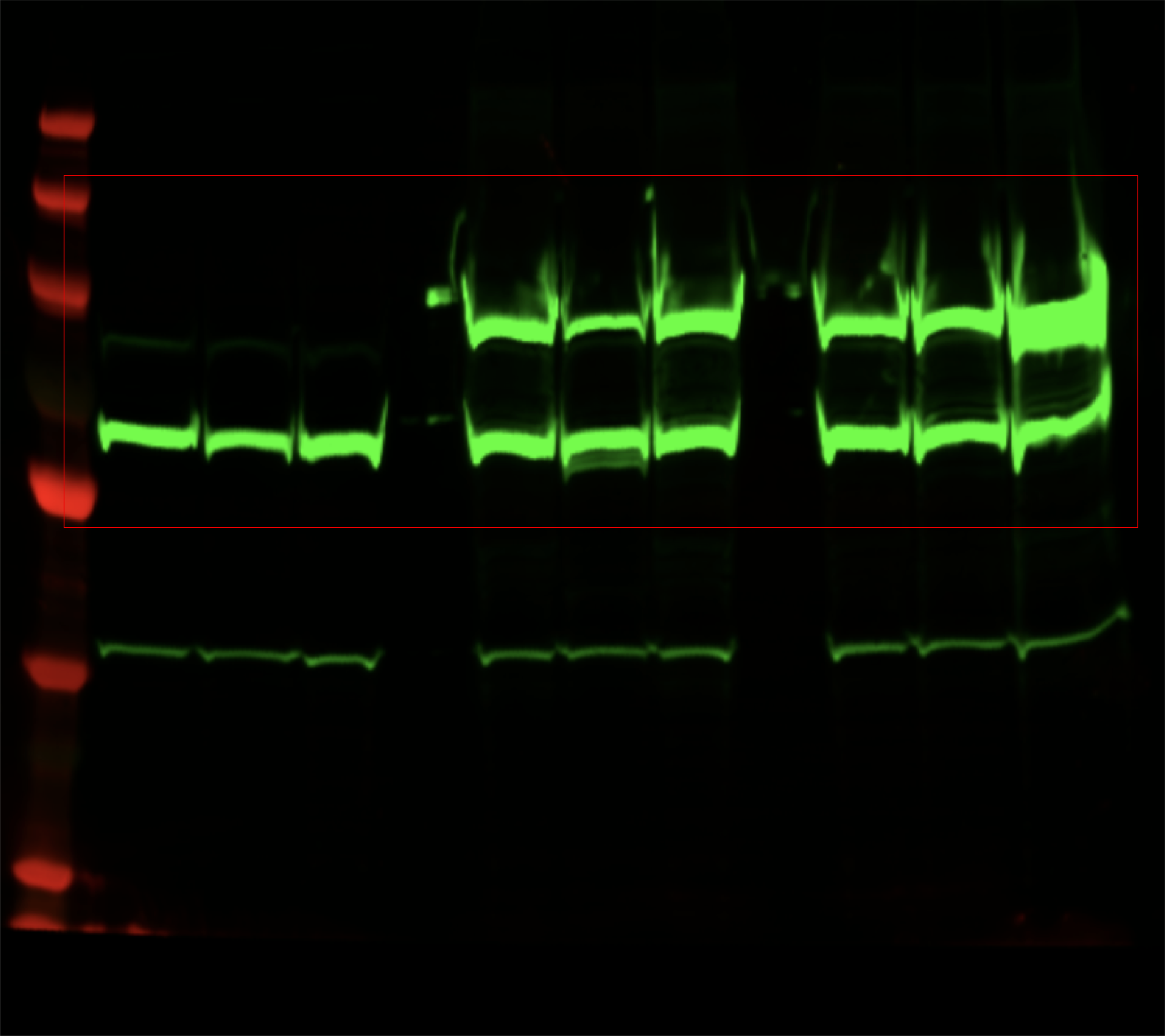

Supplement: Figure 4—source data 1. [file elife-90316-fig4-data1.zip › 4C/Exp 1/Figure 4C anti-TRMT1 Q530N labeled.tif]

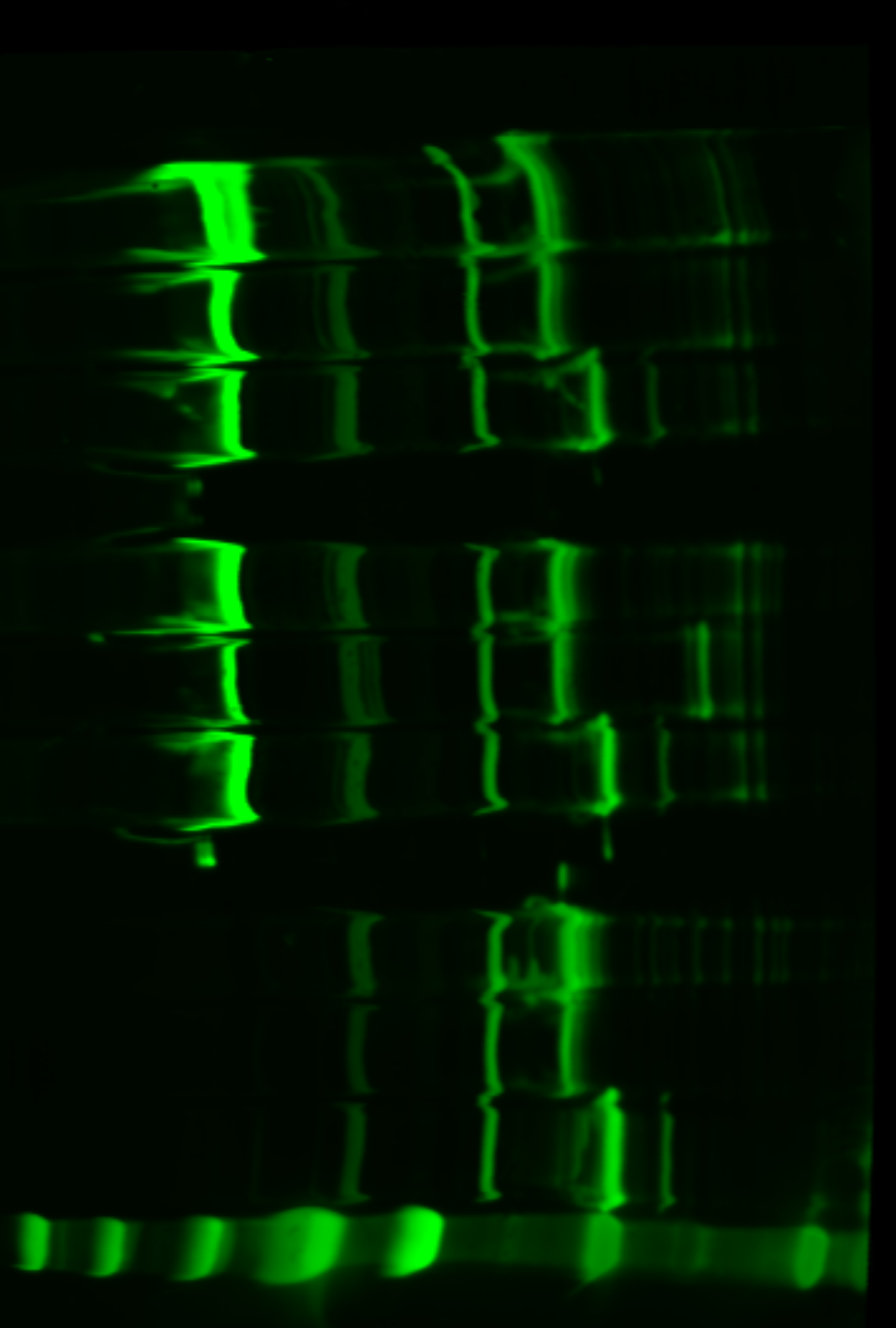

Supplement: Figure 4—source data 1. [file elife-90316-fig4-data1.zip › 4C/Exp 1/Figure 4C anti-strep Q530N.tif]

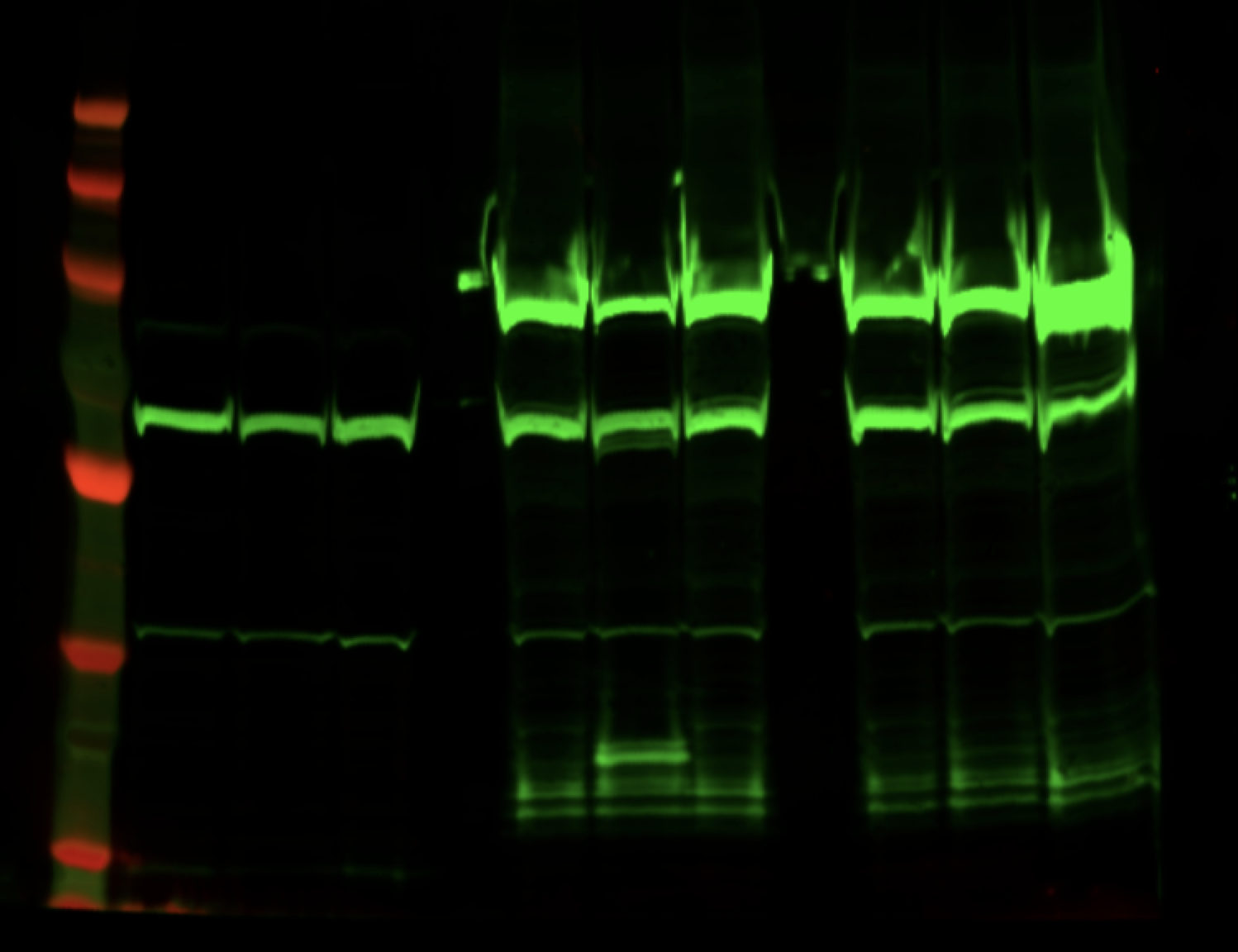

Supplement: Figure 4—source data 1. [file elife-90316-fig4-data1.zip › 4C/Exp 1/Figure 4C anti-FLAG Q503N.tif]

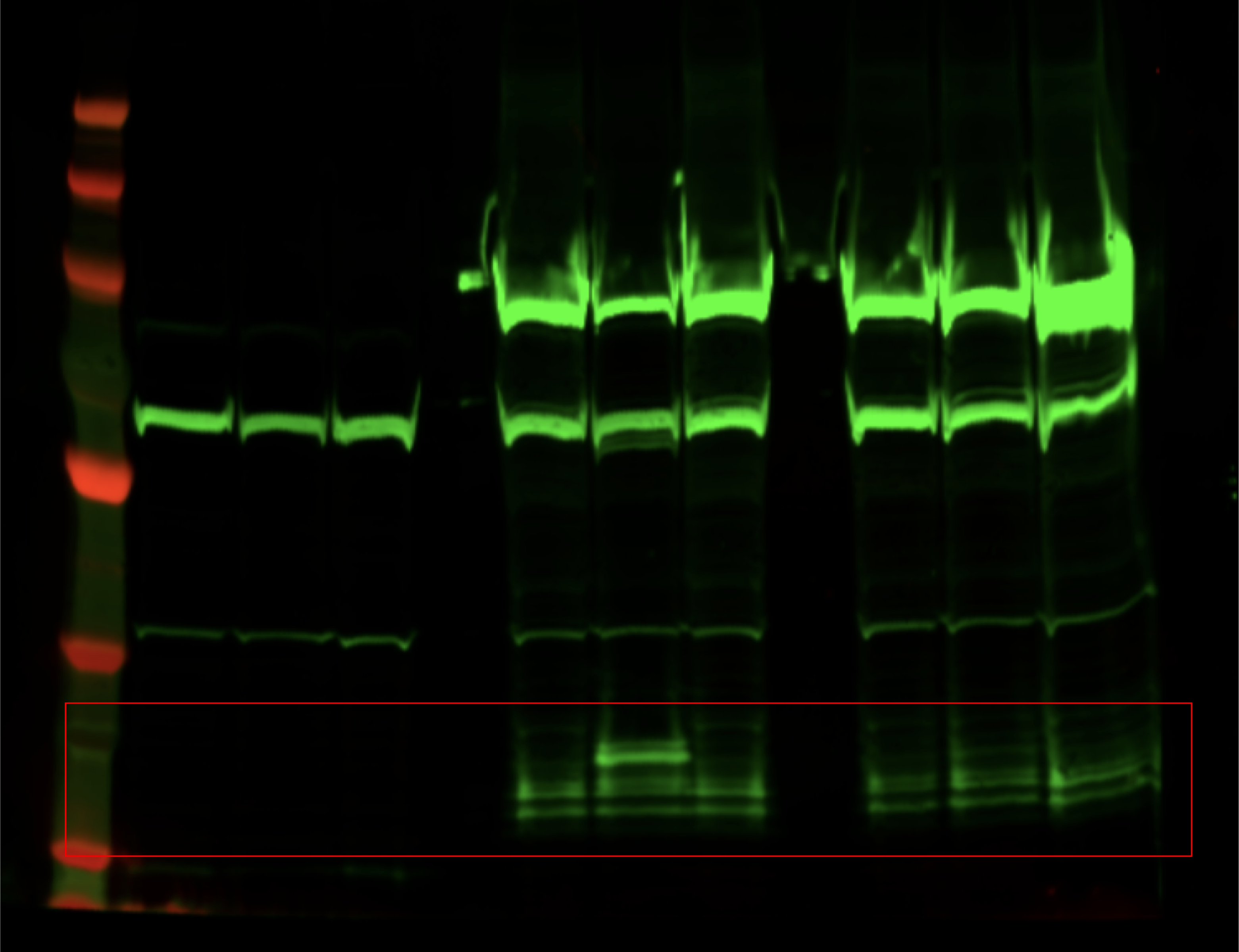

Supplement: Figure 4—source data 1. [file elife-90316-fig4-data1.zip › 4C/Exp 1/Figure 4C anti-FLAG Q503N labeled.tif]

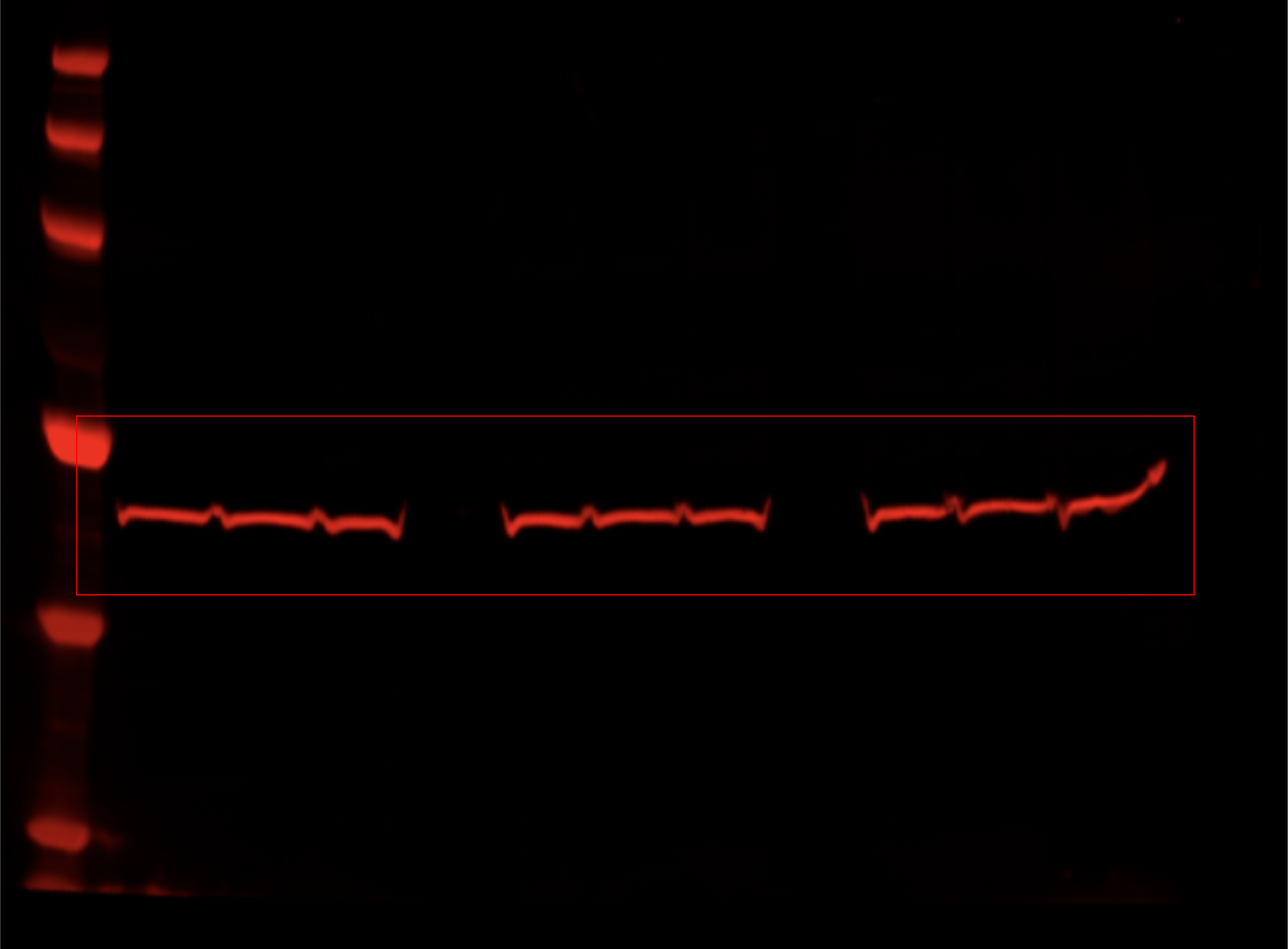

Supplement: Figure 4—source data 1. [file elife-90316-fig4-data1.zip › 4C/Exp 1/Figure 4C anti-actin Q530N labeled.tif]

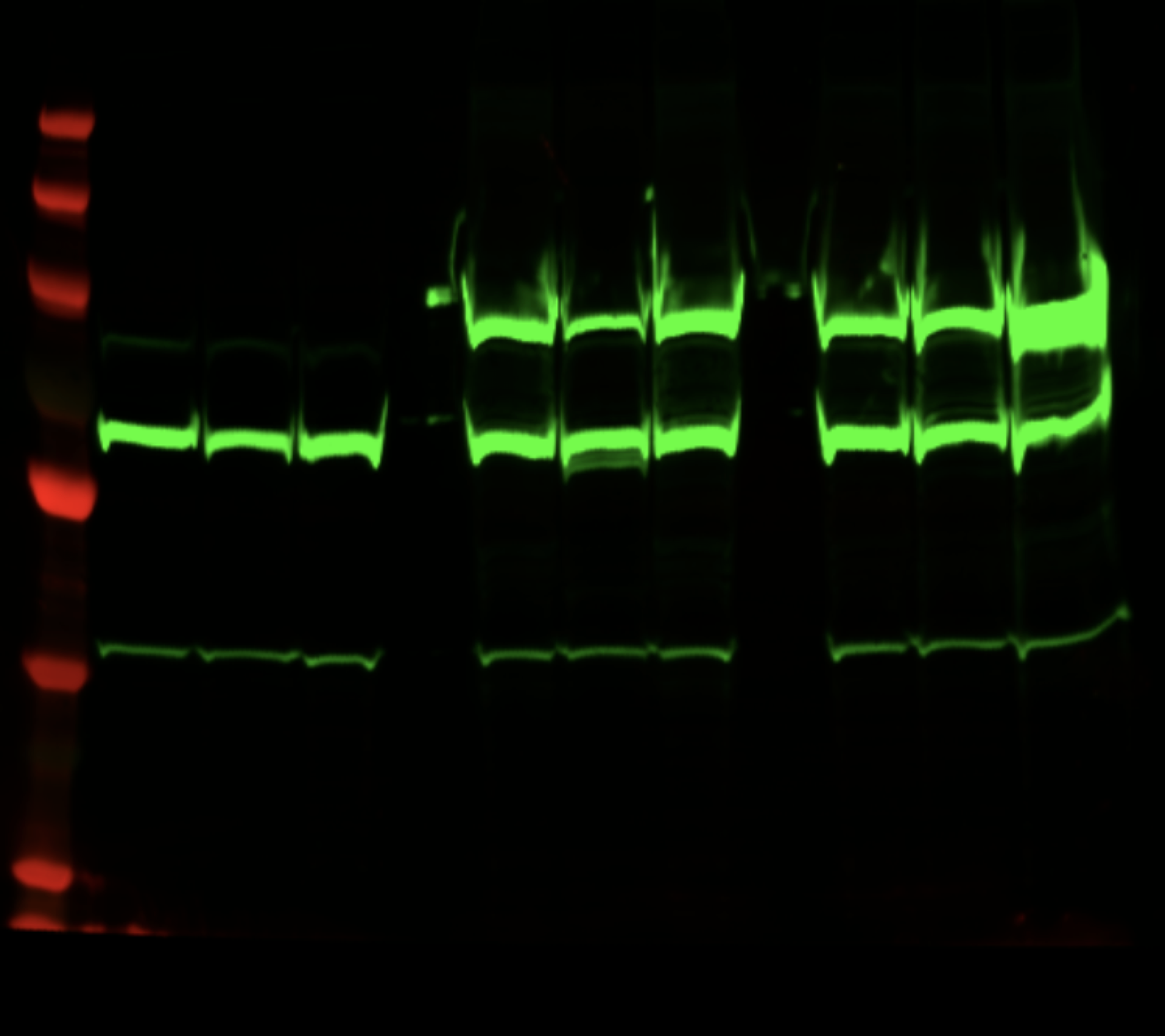

Supplement: Figure 4—source data 1. [file elife-90316-fig4-data1.zip › 4C/Exp 1/Figure 4C anti-TRMT1 Q530N.tif]

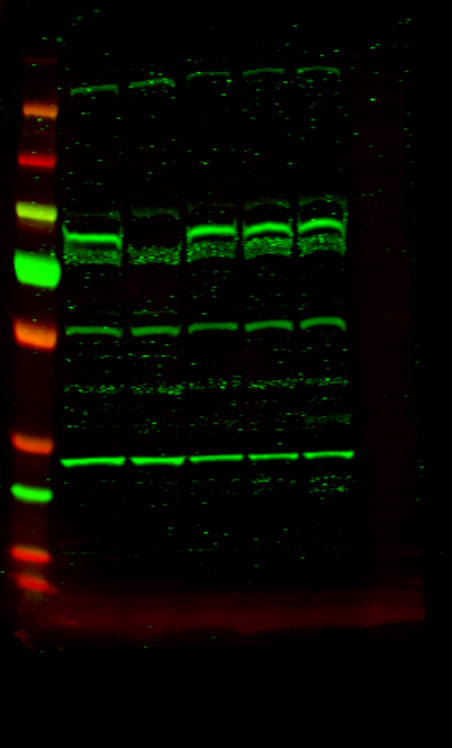

Supplement: Figure 4—figure supplement 1—source data 1. [file elife-90316-fig4-figsupp1-data1.zip › 20211101-TRMT1Cterm-293TScr KO vec NSP5 C145A.tif]

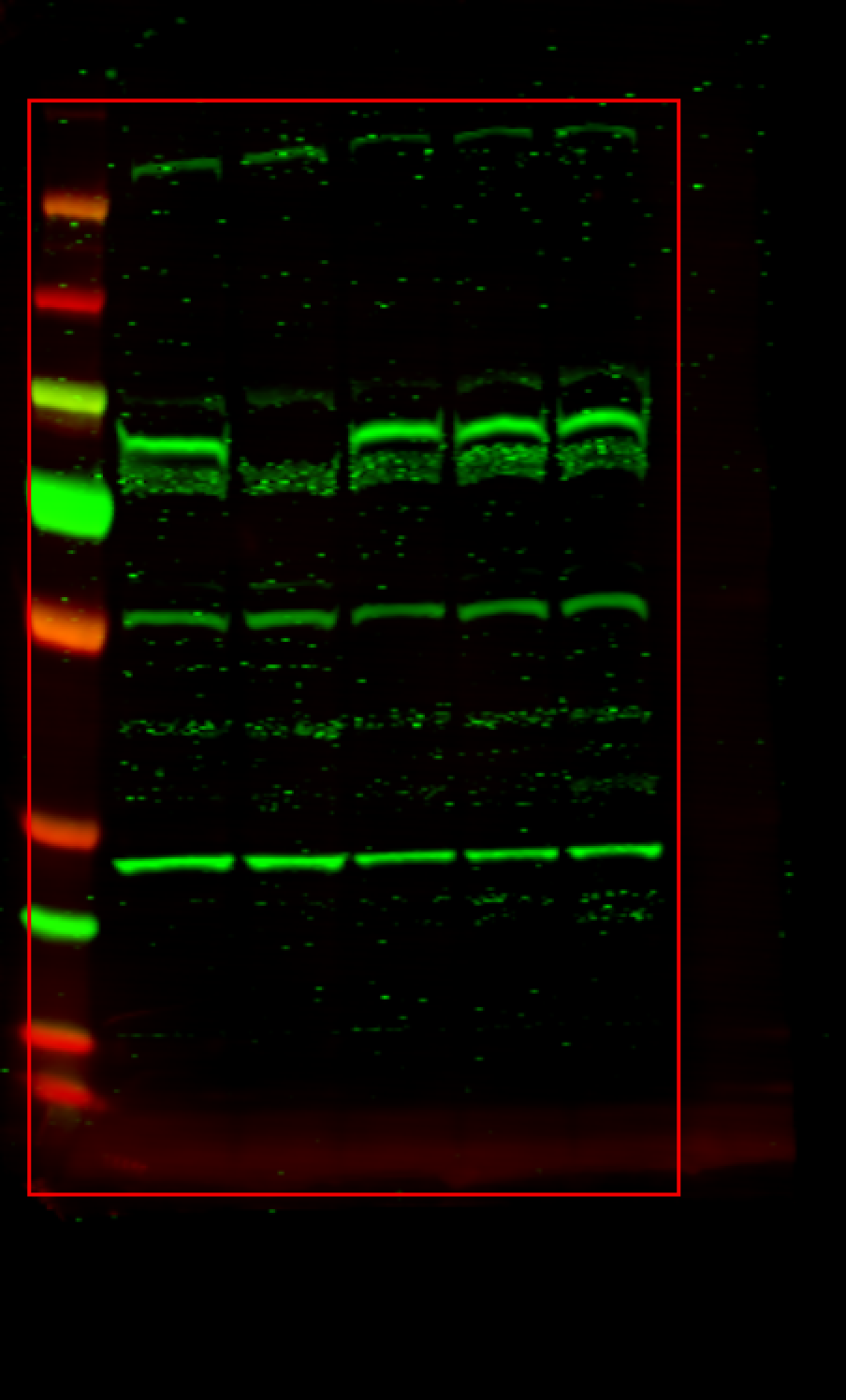

Supplement: Figure 4—figure supplement 1—source data 1. [file elife-90316-fig4-figsupp1-data1.zip › 20211101-TRMT1Cterm-293TScr KO vec NSP5 C145A labeled.tif]

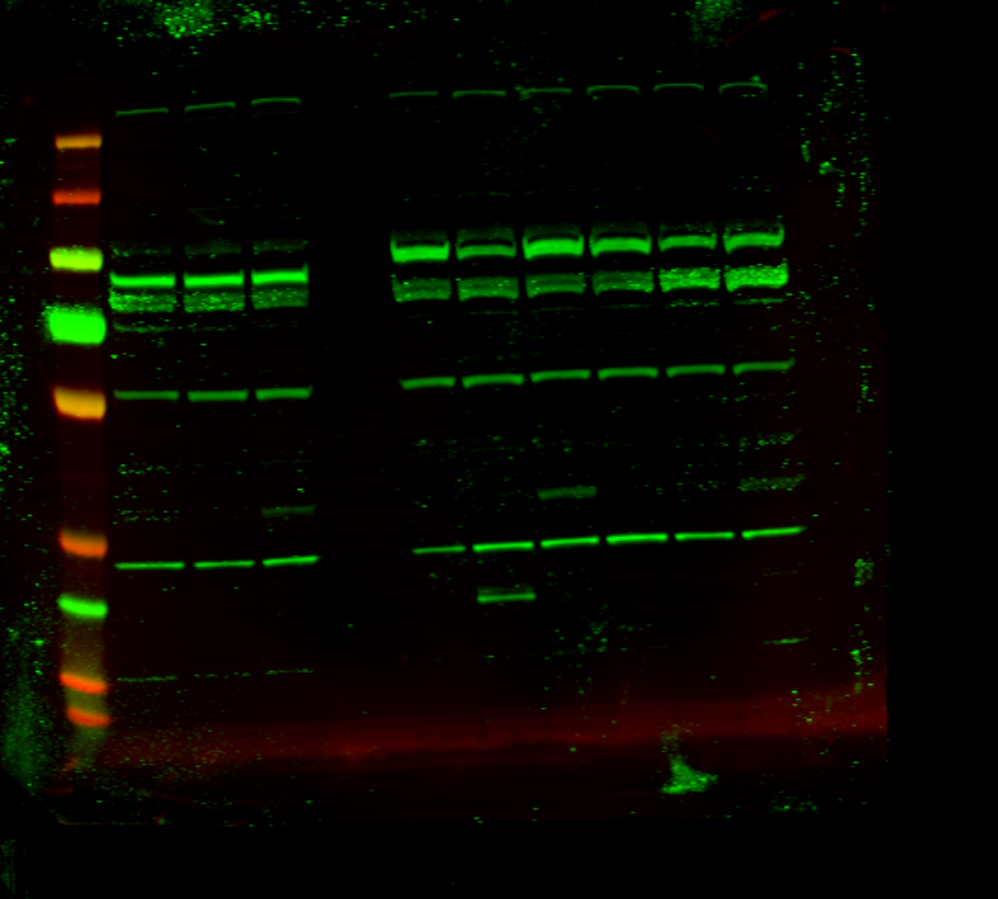

Supplement: Figure 4—figure supplement 1—source data 1. [file elife-90316-fig4-figsupp1-data1.zip › 20211118-TRMT1Cterm-293T falg-TRMT1 vec NSP5 C145A flagQ530N.tif]

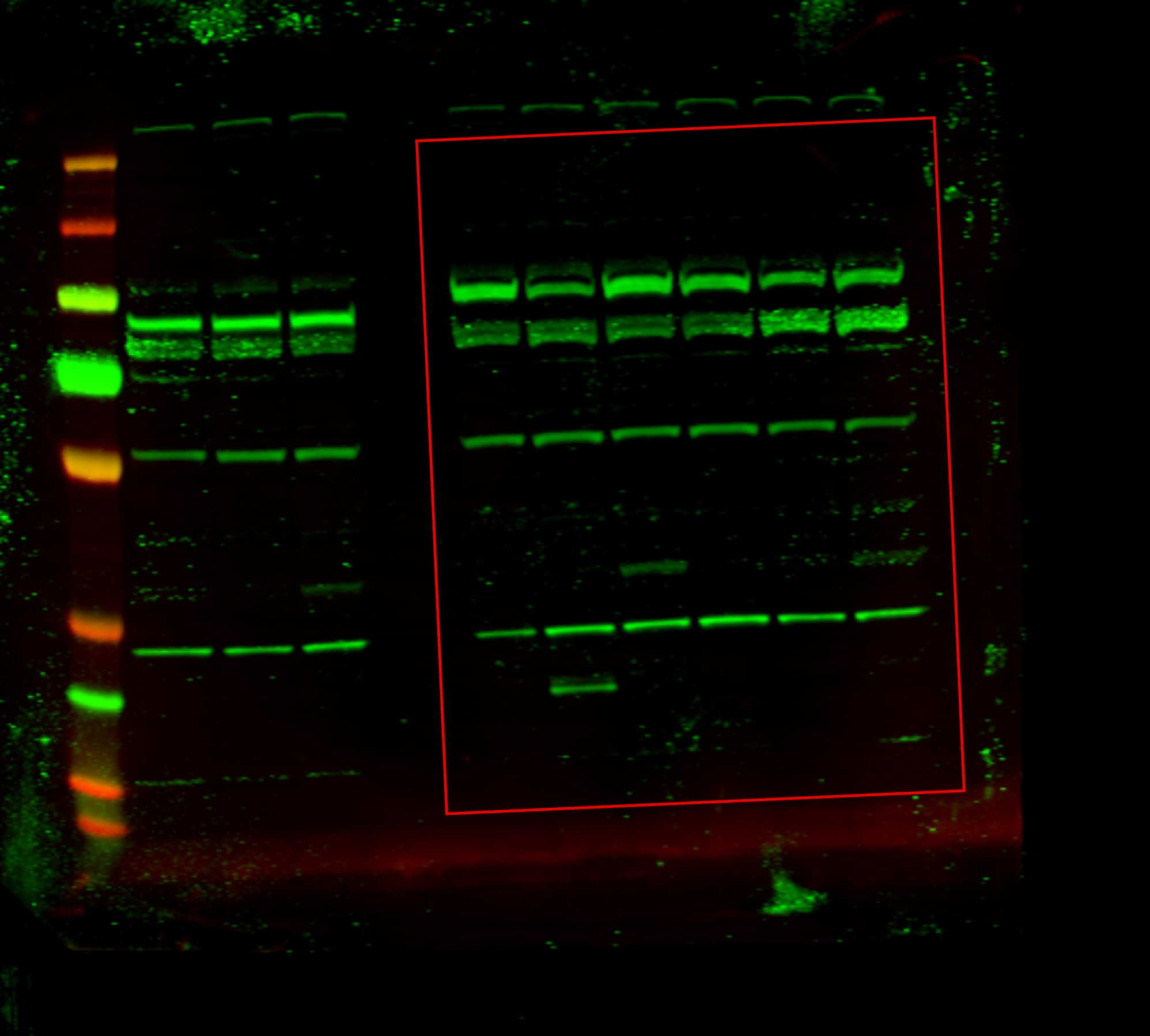

Supplement: Figure 4—figure supplement 1—source data 1. [file elife-90316-fig4-figsupp1-data1.zip › 20211118-TRMT1Cterm-293T falg-TRMT1 vec NSP5 C145A flagQ530N labeled.tif]

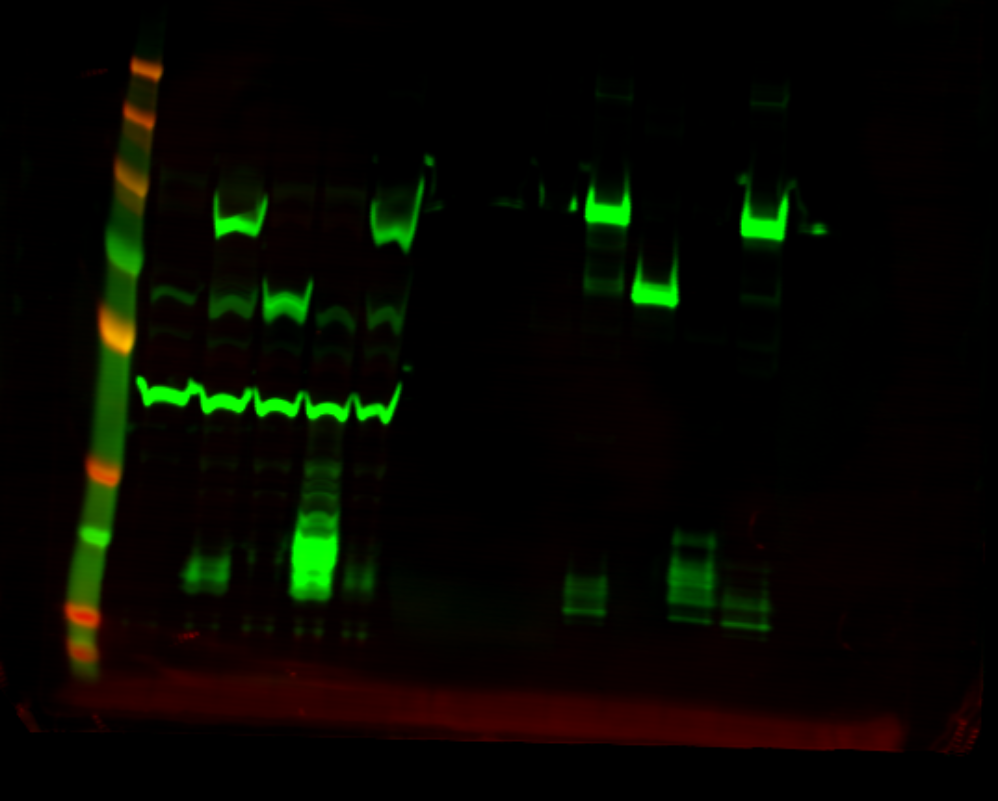

Supplement: Figure 5—source data 1. [file elife-90316-fig5-data1.zip › 5B and C/Rep 2/Figure 5B 082121-actin-purification flag-TRMT1 fragments.tif]

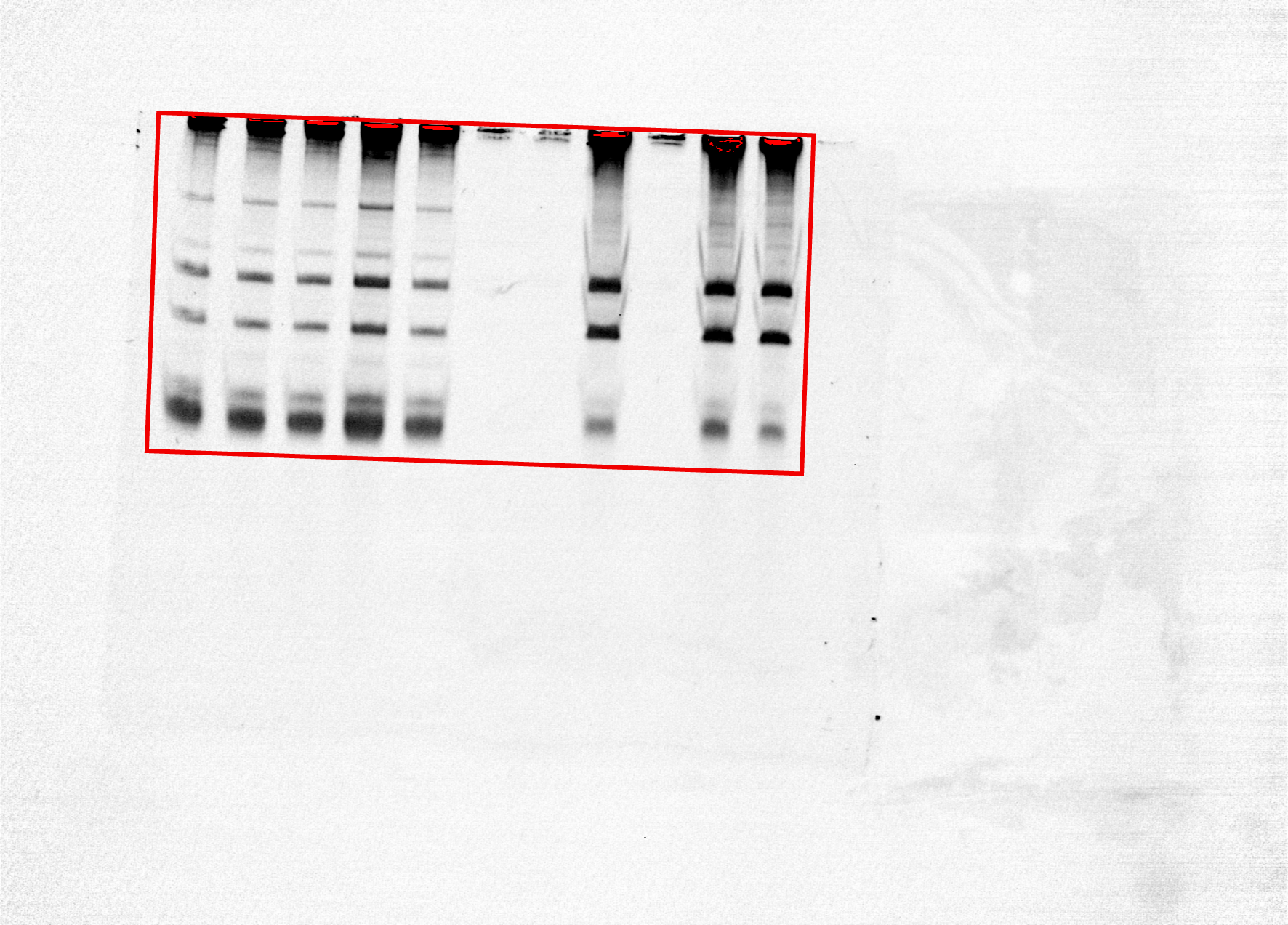

Supplement: Figure 5—source data 1. [file elife-90316-fig5-data1.zip › 5B and C/Rep 2/Figure 5C 20210826 SYBRGOLD 293T- TRMT1 fragments labeled.tif]

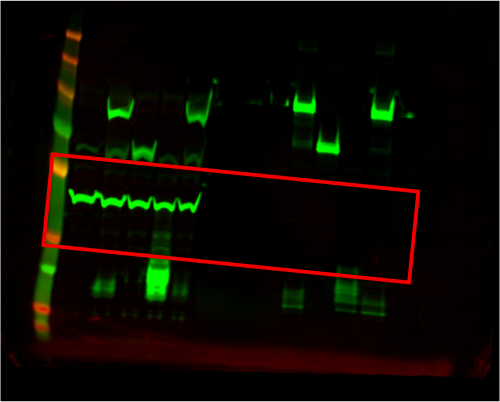

Supplement: Figure 5—source data 1. [file elife-90316-fig5-data1.zip › 5B and C/Rep 2/Figure 5B 082121-actin-purification flag-TRMT1 fragments labeled.tif]

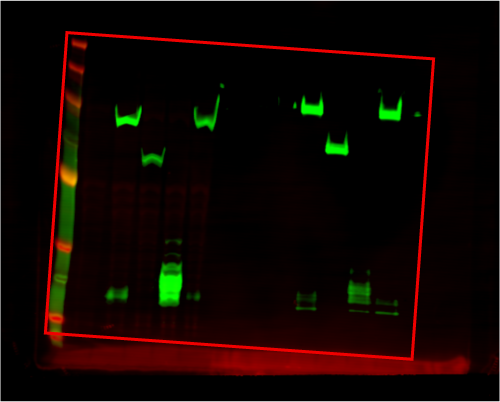

Supplement: Figure 5—source data 1. [file elife-90316-fig5-data1.zip › 5B and C/Rep 2/Figure 5B 082121-flag-purification flag-TRMT1 fragments labeled.tif]

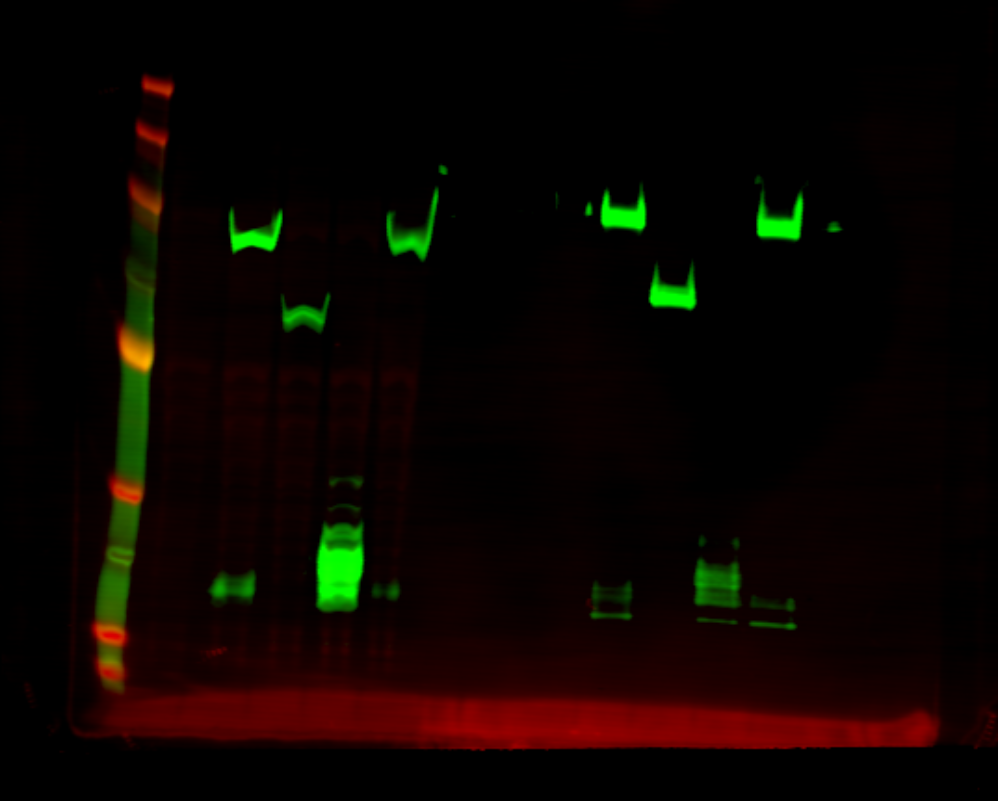

Supplement: Figure 5—source data 1. [file elife-90316-fig5-data1.zip › 5B and C/Rep 2/Figure 5B 082121-flag-purification flag-TRMT1 fragments.tif]

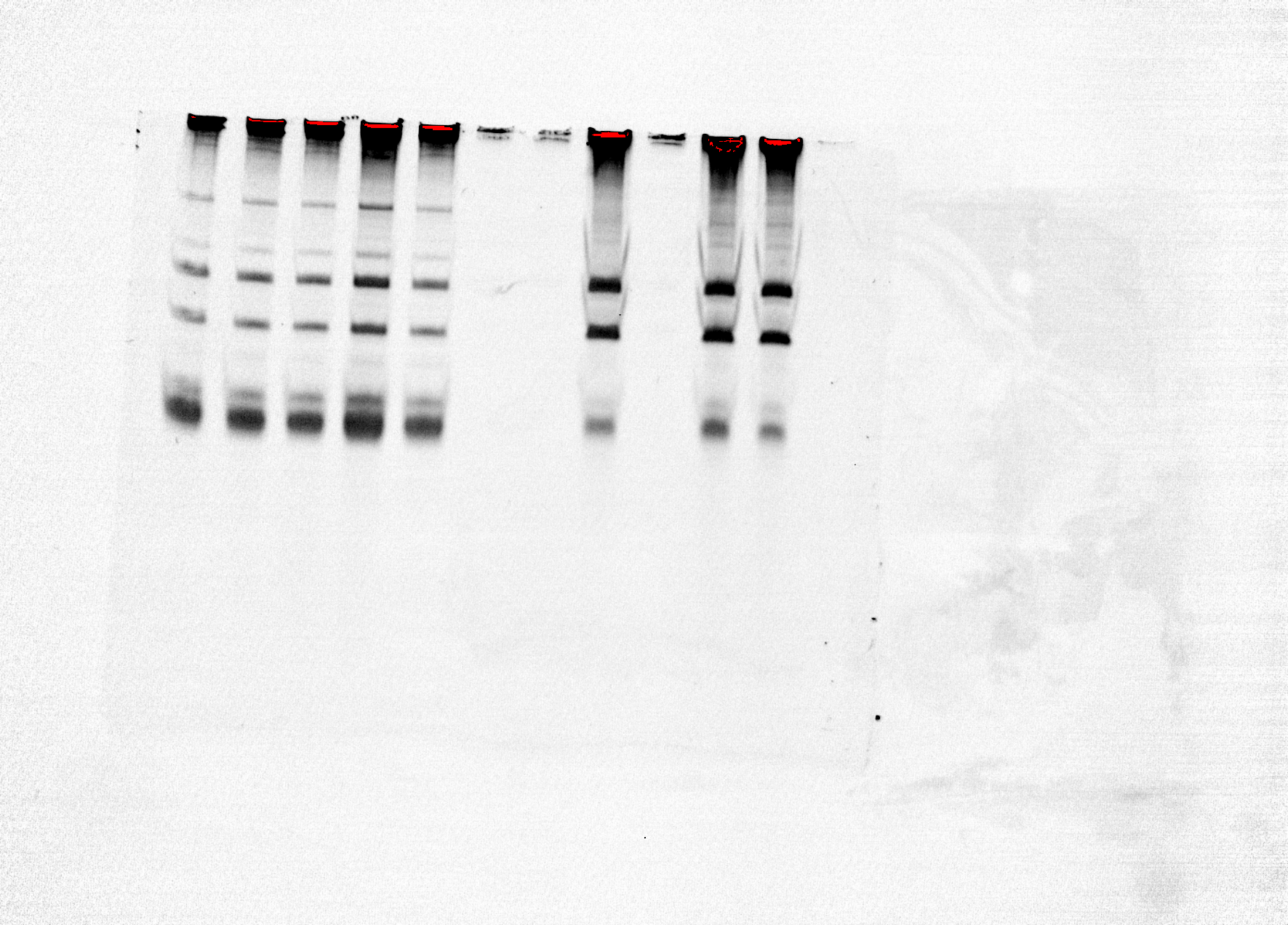

Supplement: Figure 5—source data 1. [file elife-90316-fig5-data1.zip › 5B and C/Rep 2/Figure 5C 20210826 SYBRGOLD 293T- TRMT1 fragments.tif]

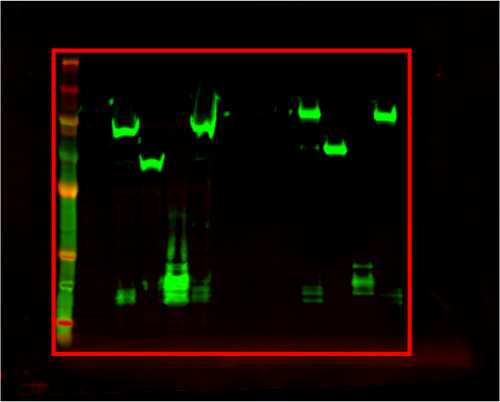

Supplement: Figure 5—source data 1. [file elife-90316-fig5-data1.zip › 5B and C/Rep 1/Figure 5B 073121-flag-purification flag-TRMT1 fragments labeled.tif]

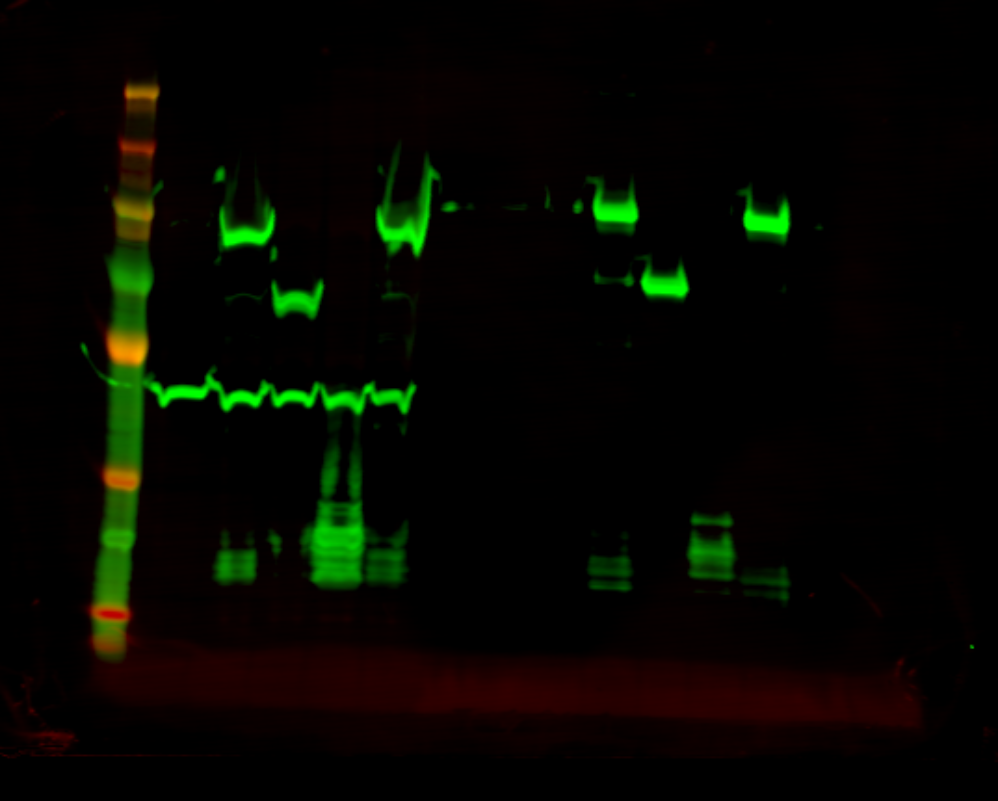

Supplement: Figure 5—source data 1. [file elife-90316-fig5-data1.zip › 5B and C/Rep 1/Figure 5B 073121-actin-purification flag-TRMT1 fragments.tif]

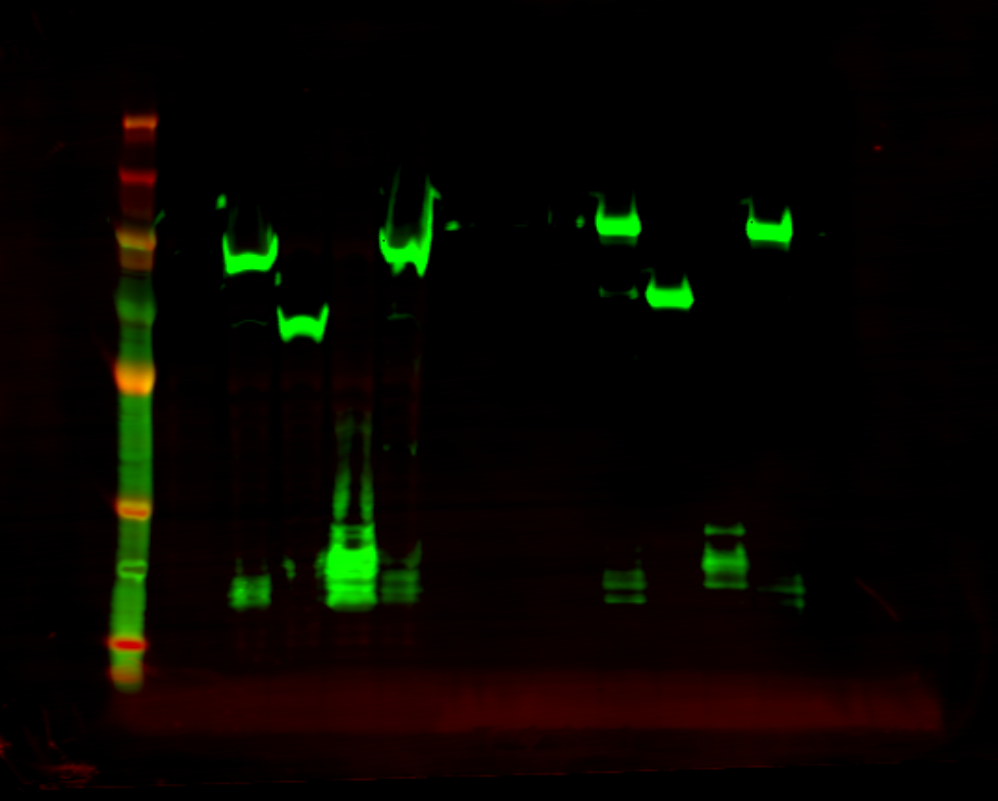

Supplement: Figure 5—source data 1. [file elife-90316-fig5-data1.zip › 5B and C/Rep 1/Figure 5B 073121-flag-purification flag-TRMT1 fragments.tif]

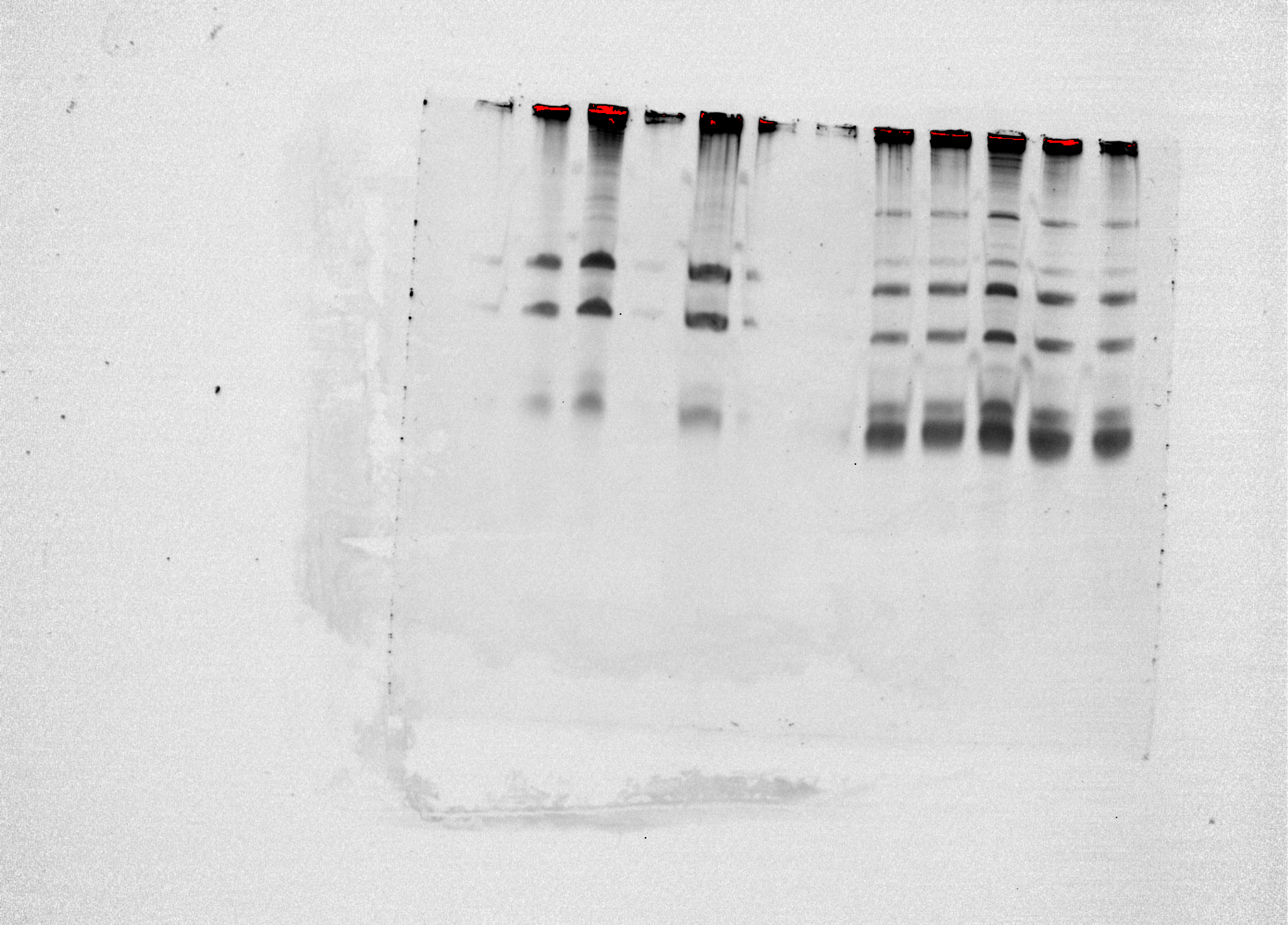

Supplement: Figure 5—source data 1. [file elife-90316-fig5-data1.zip › 5B and C/Rep 1/Figure 5C 20210804 SYBRGOLD 293T- TRMT1 fragments-2.tif]

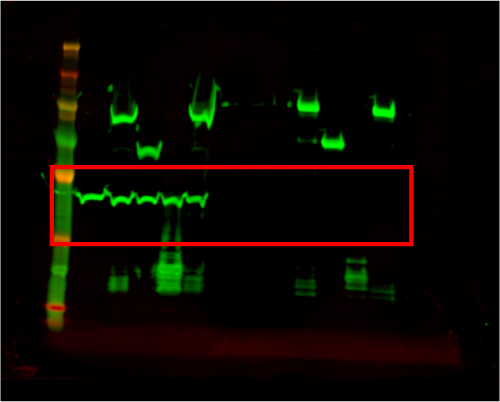

Supplement: Figure 5—source data 1. [file elife-90316-fig5-data1.zip › 5B and C/Rep 1/Figure 5B 073121-actin-purification flag-TRMT1 fragments labeled.tif]

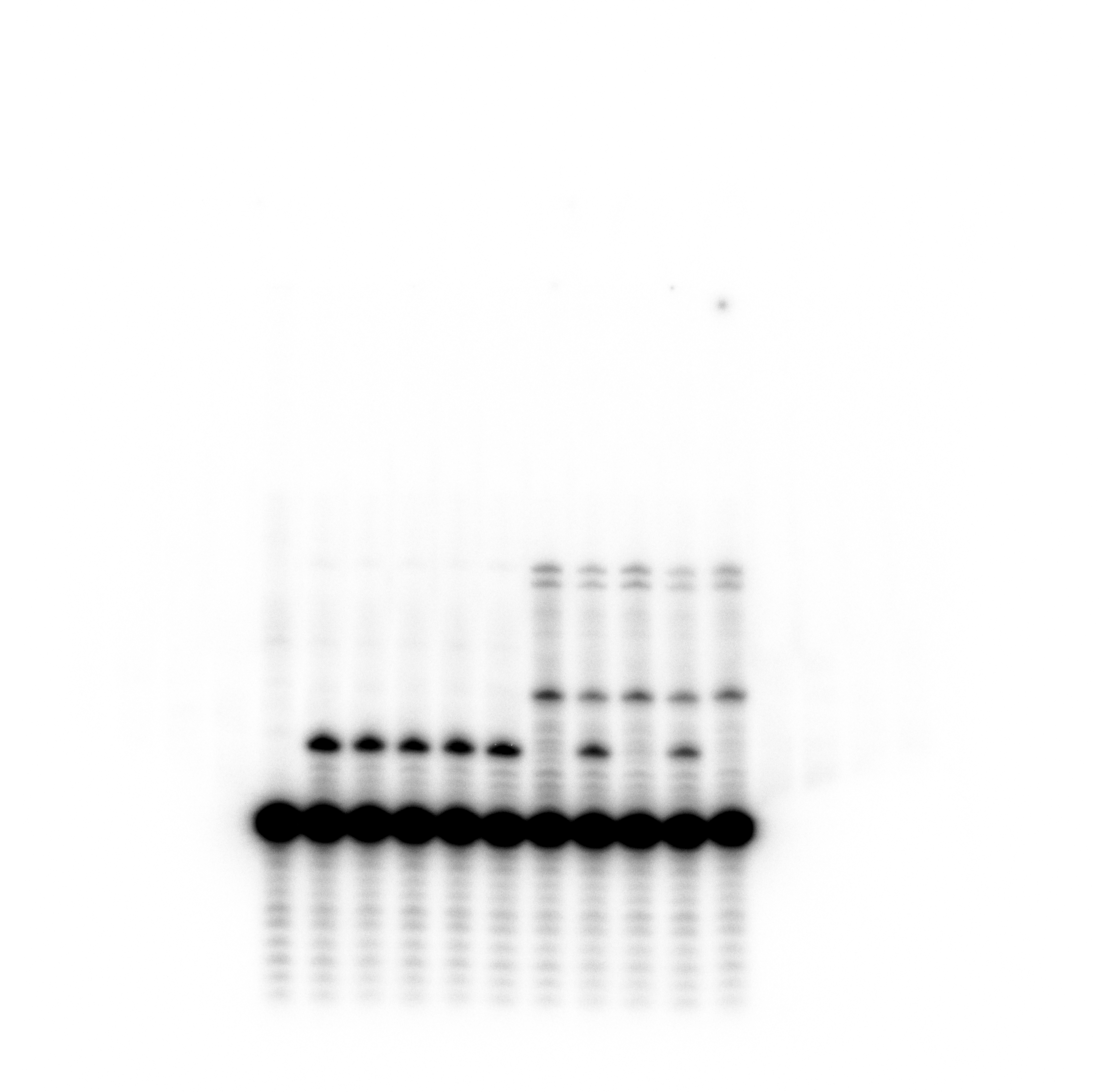

Supplement: Figure 5—source data 1. [file elife-90316-fig5-data1.zip › 5D and E/Figure 5E #82 082121 PE M44.tif]

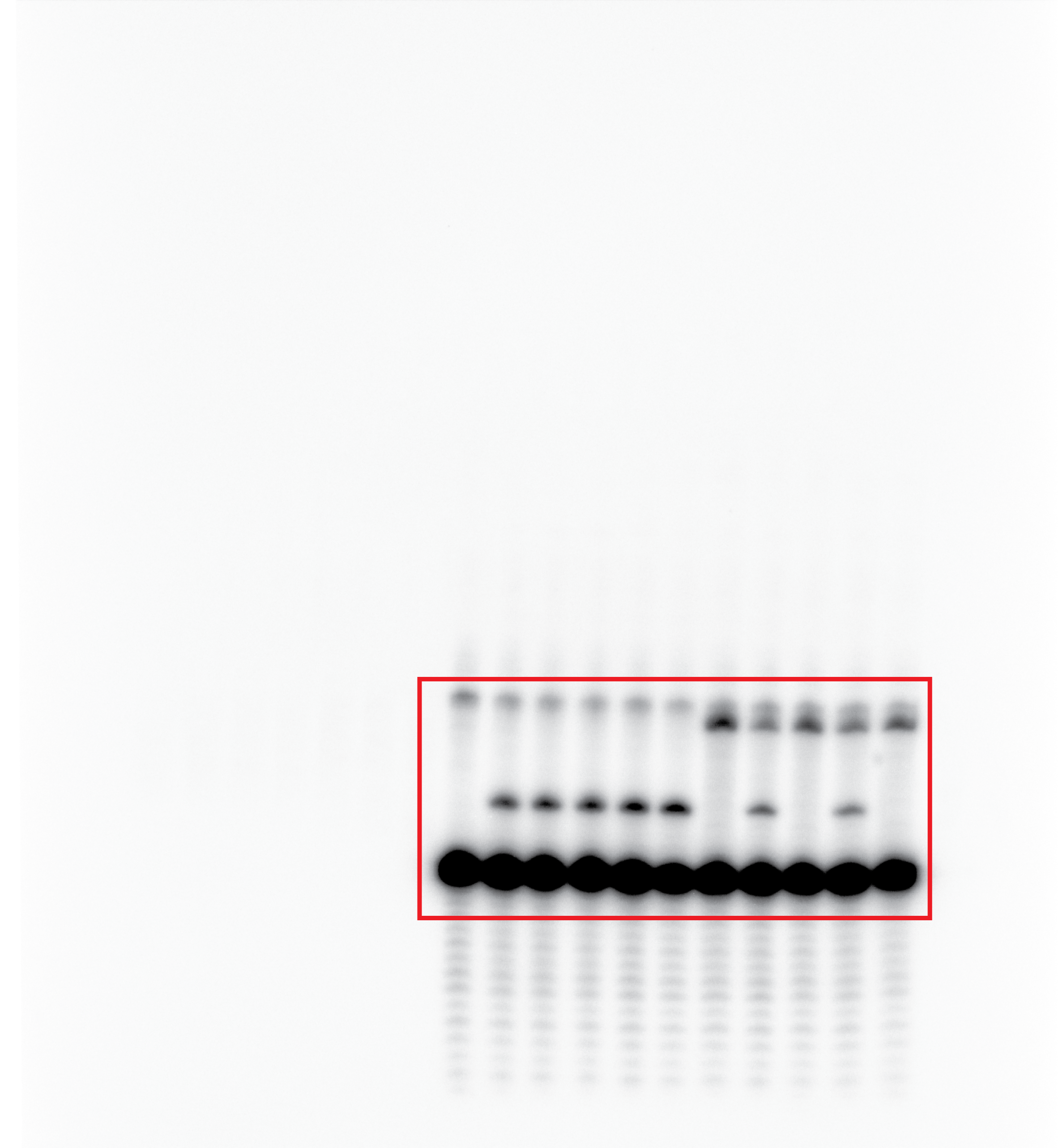

Supplement: Figure 5—source data 1. [file elife-90316-fig5-data1.zip › 5D and E/Figure 5E #81 081921 PE M43 labeled.tif]

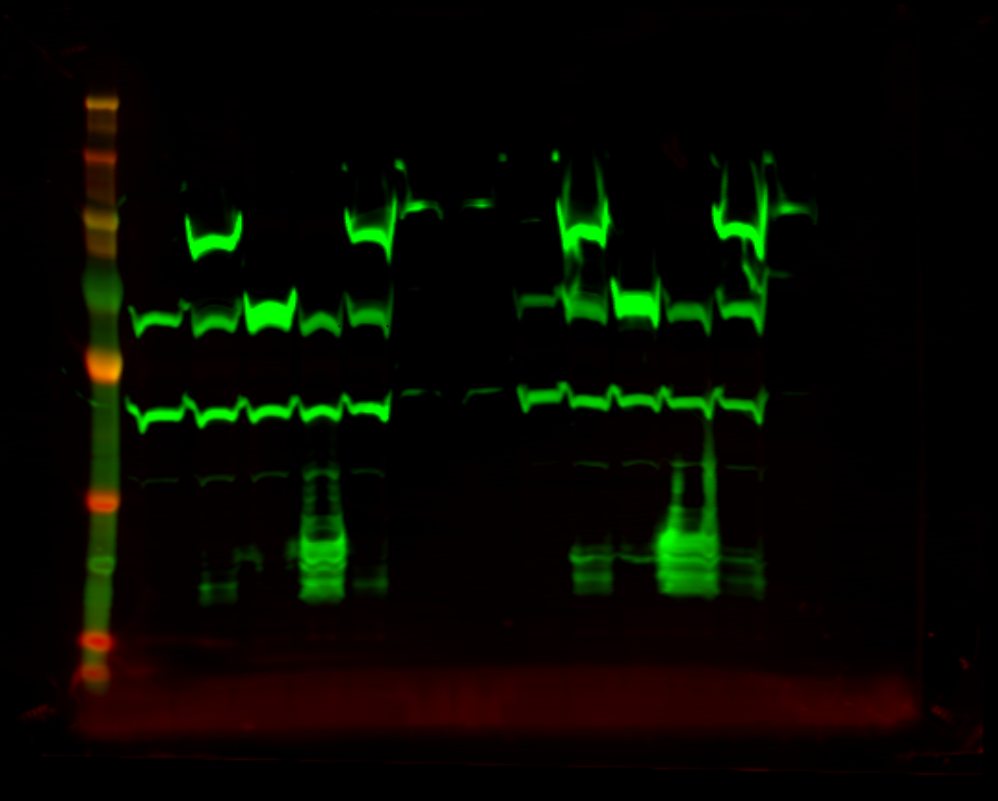

Supplement: Figure 5—source data 1. [file elife-90316-fig5-data1.zip › 5D and E/Figure 5D 081121-actin-Scr KO cell TRTM1-flag fragments transfection.tif]

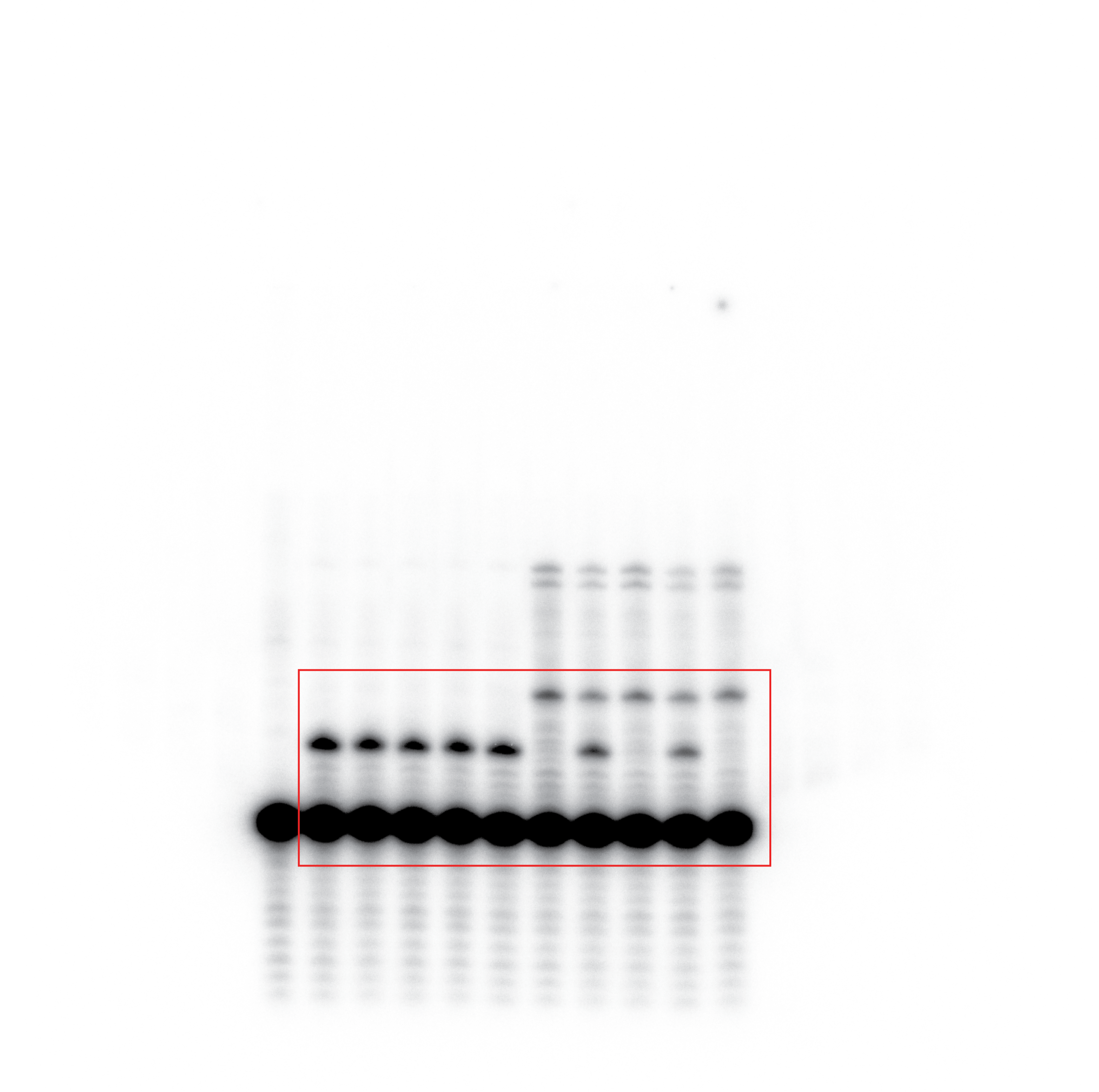

Supplement: Figure 5—source data 1. [file elife-90316-fig5-data1.zip › 5D and E/Figure 5E #82 082121 PE M44 labeled.tif]

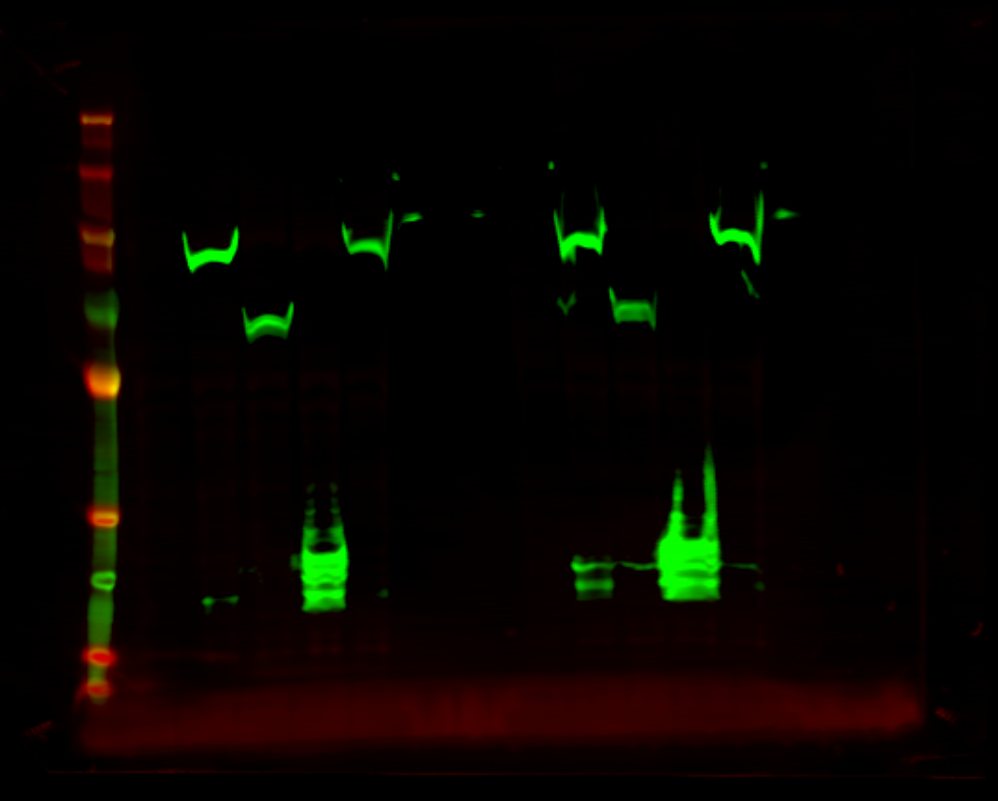

Supplement: Figure 5—source data 1. [file elife-90316-fig5-data1.zip › 5D and E/Figure 5D 081121-flag-Scr KO cell TRTM1-flag fragments transfection.tif]

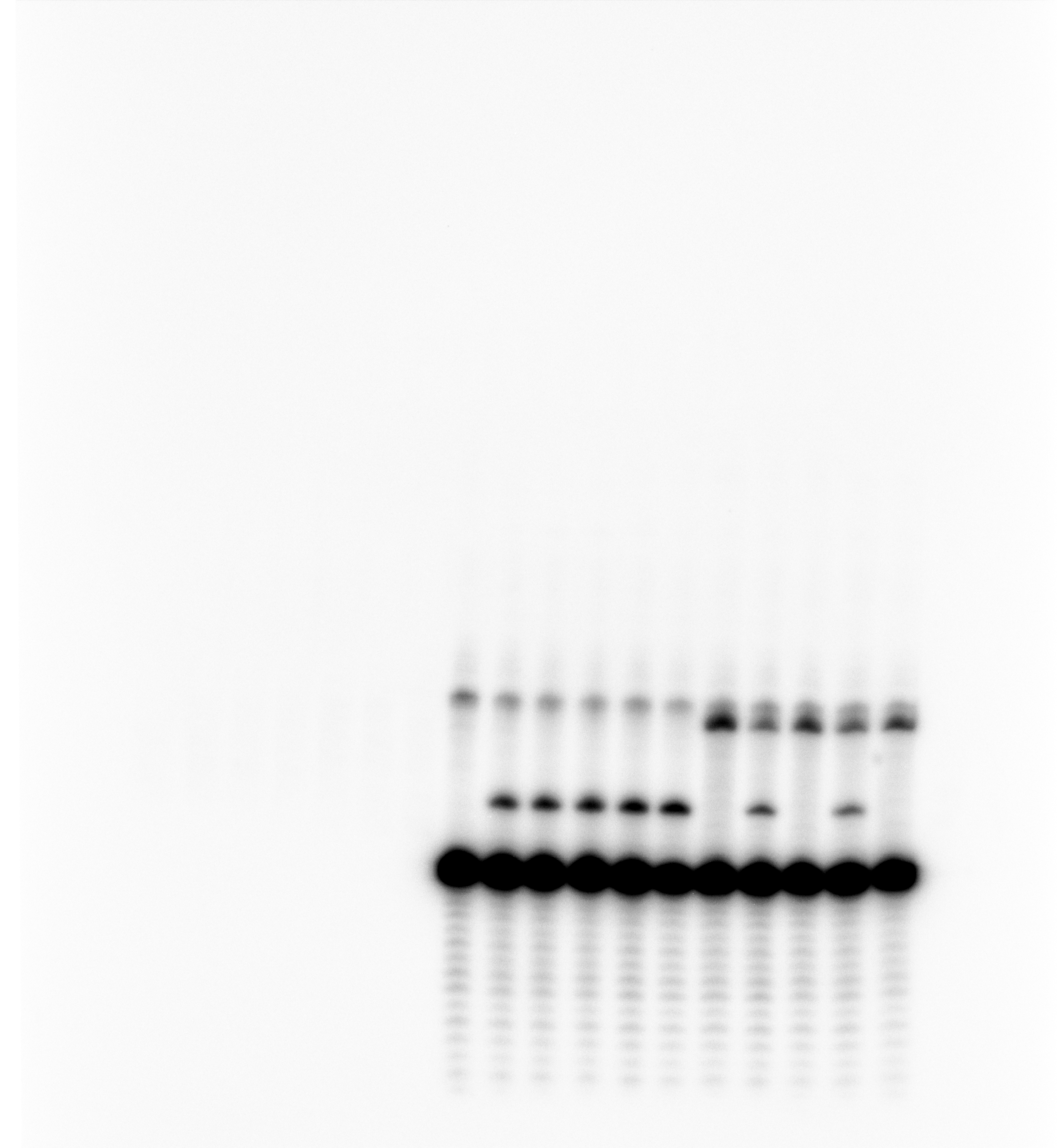

Supplement: Figure 5—source data 1. [file elife-90316-fig5-data1.zip › 5D and E/Figure 5E #81 081921 PE M43.tif]

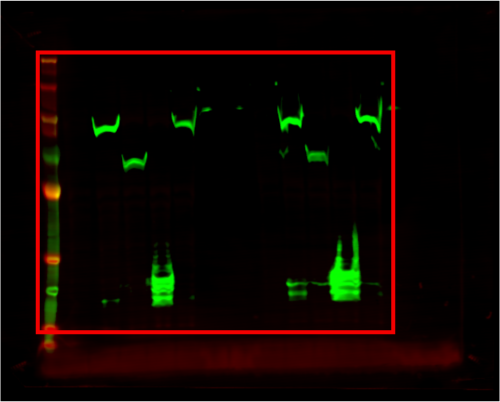

Supplement: Figure 5—source data 1. [file elife-90316-fig5-data1.zip › 5D and E/Figure 5D 081121-flag-Scr KO cell TRTM1-flag fragments transfection labeled.tif]

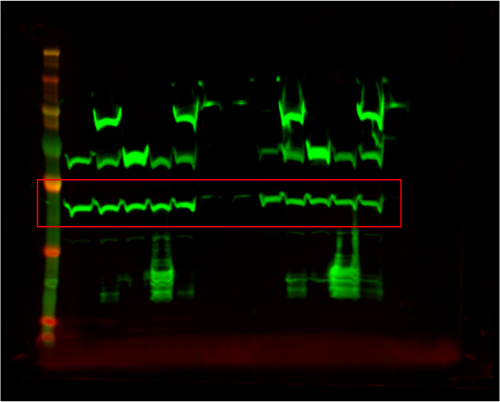

Supplement: Figure 5—source data 1. [file elife-90316-fig5-data1.zip › 5D and E/Figure 5D 081121-actin-Scr KO cell TRTM1-flag fragments transfection labeled.tif]

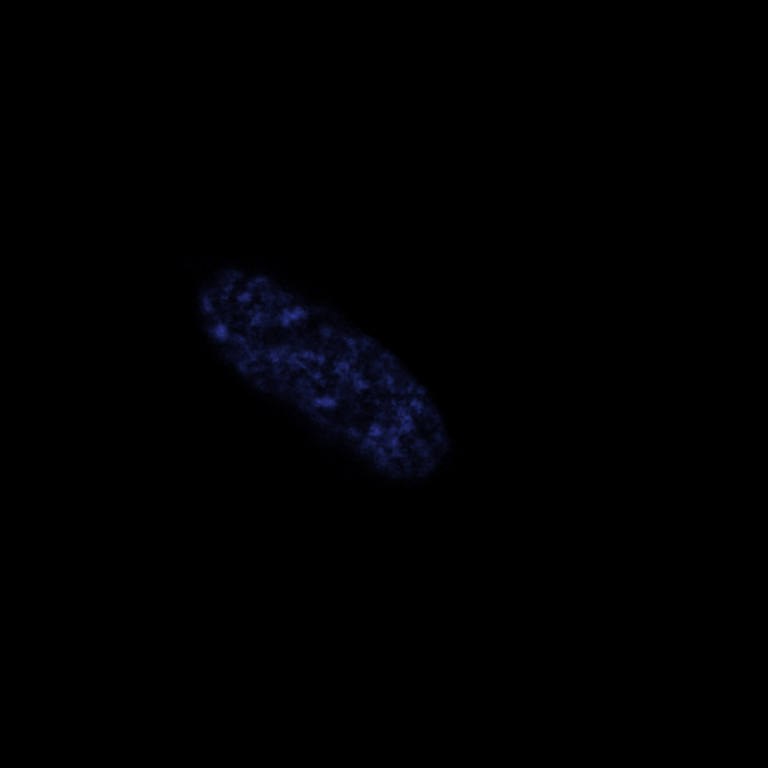

Supplement: Figure 5—figure supplement 1—source data 1. [file elife-90316-fig5-figsupp1-data1.zip › 1-530-GFP-blue.png]

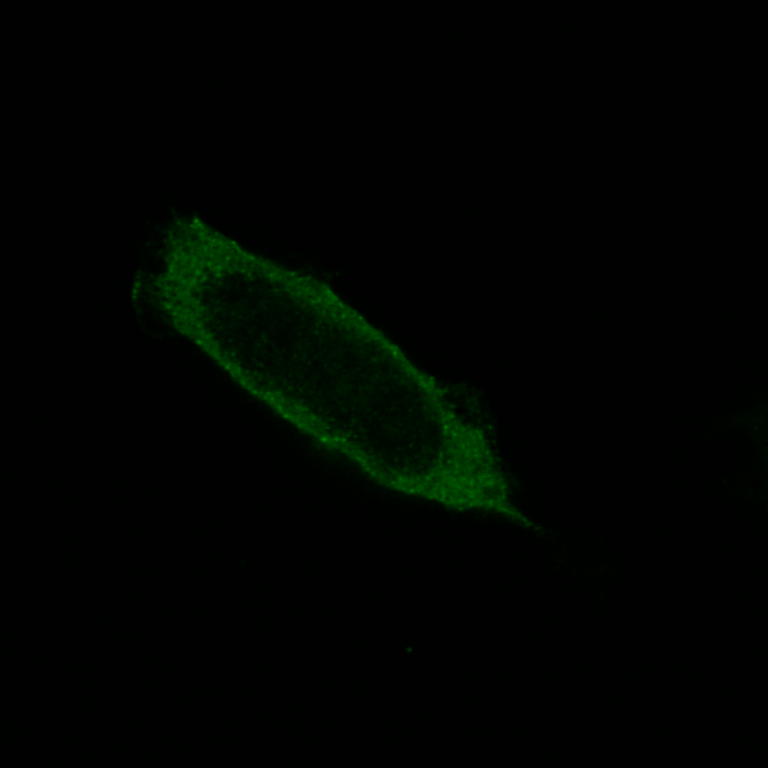

Supplement: Figure 5—figure supplement 1—source data 1. [file elife-90316-fig5-figsupp1-data1.zip › 1-530-GFP-green.png]

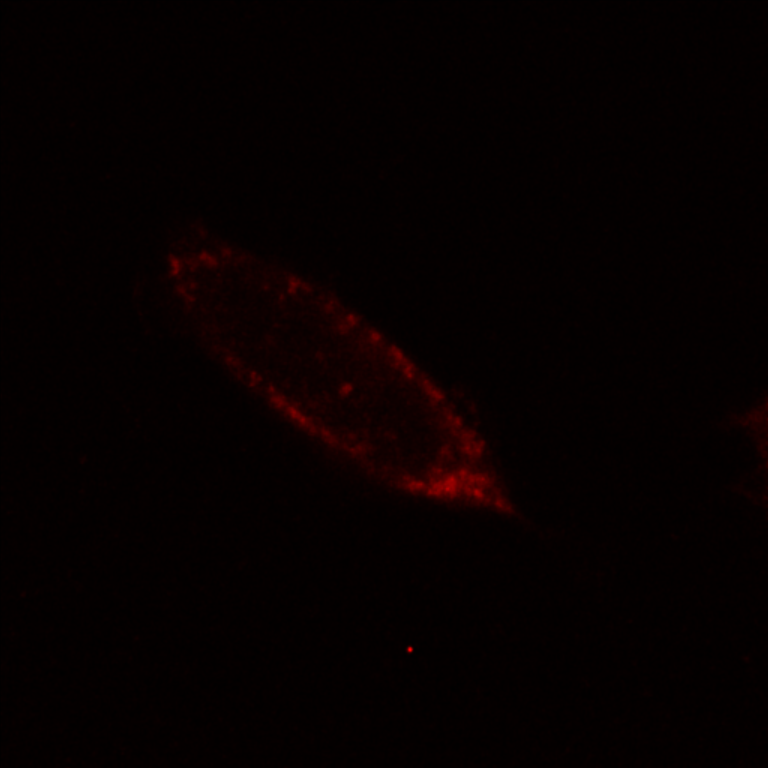

Supplement: Figure 5—figure supplement 1—source data 1. [file elife-90316-fig5-figsupp1-data1.zip › 1-530-GFP-red.png]

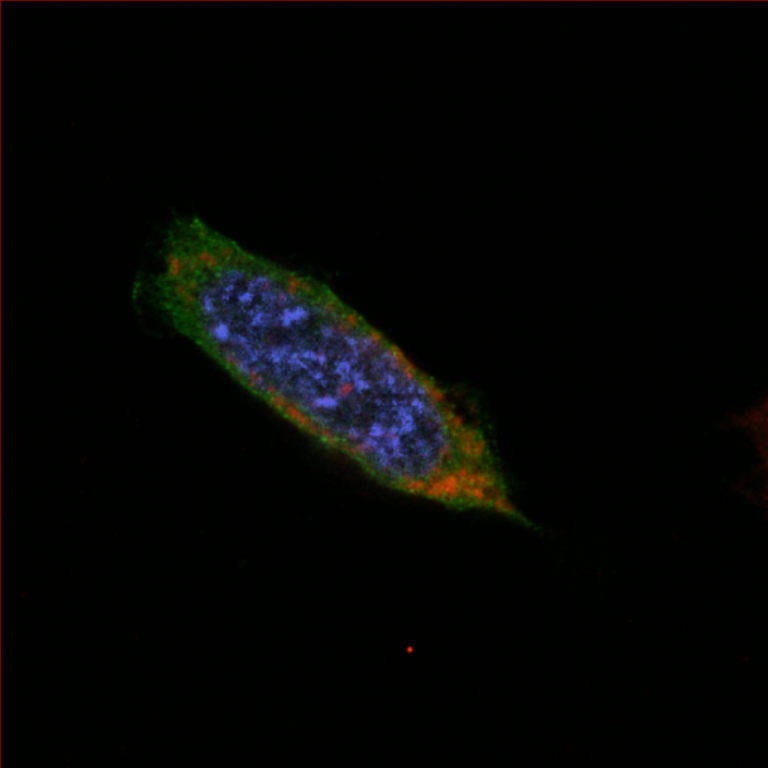

Supplement: Figure 5—figure supplement 1—source data 1. [file elife-90316-fig5-figsupp1-data1.zip › 1-530-GFP-total.png]

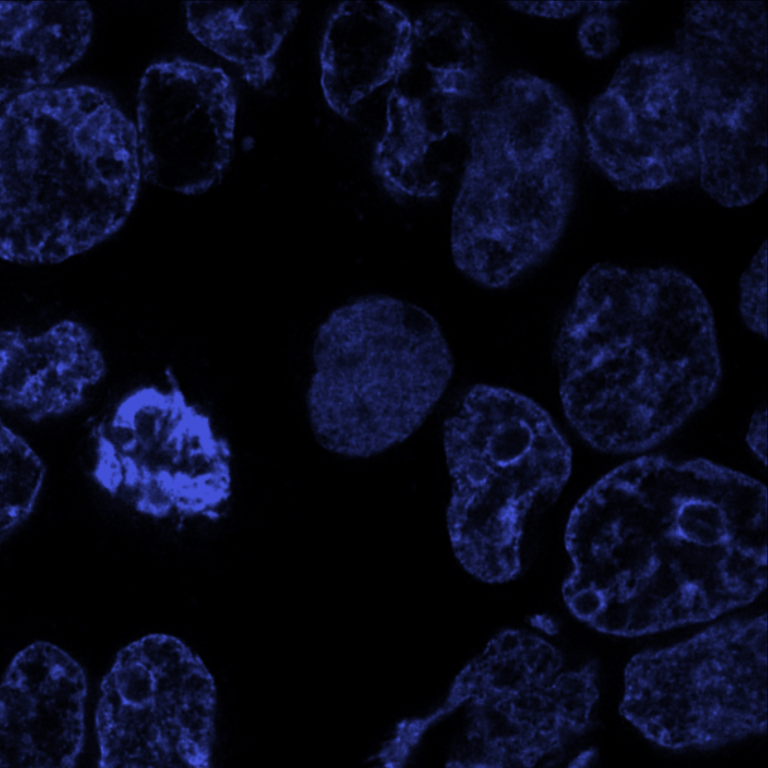

Supplement: Figure 5—figure supplement 1—source data 1. [file elife-90316-fig5-figsupp1-data1.zip › 530-end-GFP-blue.png]

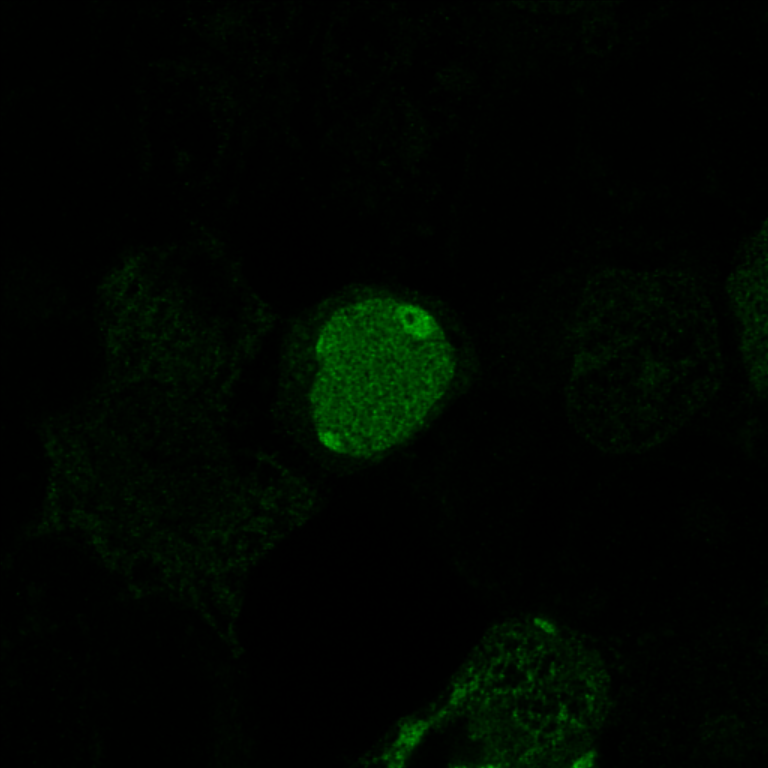

Supplement: Figure 5—figure supplement 1—source data 1. [file elife-90316-fig5-figsupp1-data1.zip › 530-end-GFP-green.png]

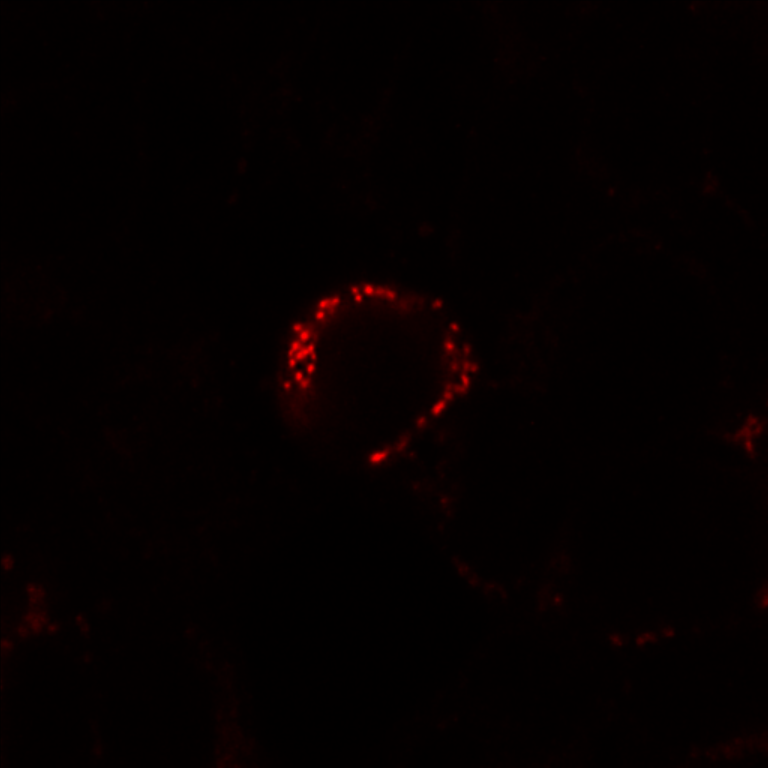

Supplement: Figure 5—figure supplement 1—source data 1. [file elife-90316-fig5-figsupp1-data1.zip › 530-end-GFP-red.png]

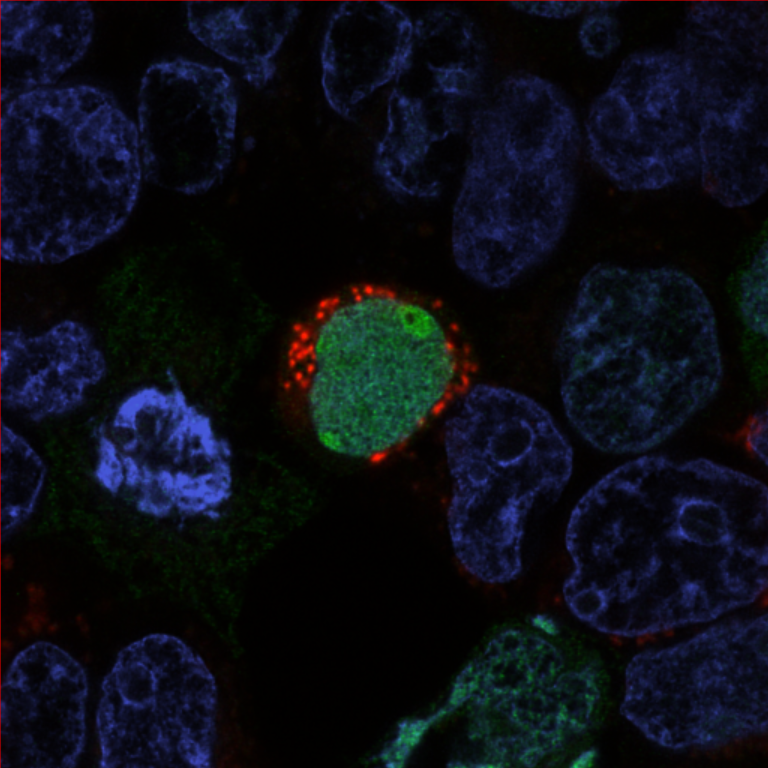

Supplement: Figure 5—figure supplement 1—source data 1. [file elife-90316-fig5-figsupp1-data1.zip › 530-end-GFP-total.png]

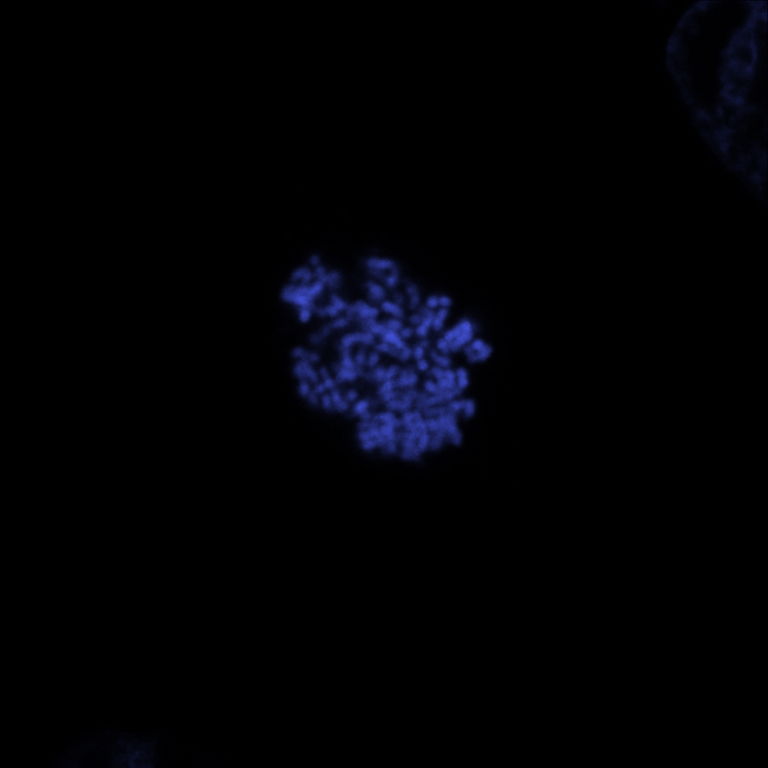

Supplement: Figure 5—figure supplement 1—source data 1. [file elife-90316-fig5-figsupp1-data1.zip › TRMT1-GFP-blue.png]
