# Supplementary figures and images for: Proteolytic cleavage and inactivation of the TRMT1 tRNA modification enzyme by SARS-CoV-2 main protease (part 2 of 2)
Source: eLife. 2024 May 30;12:RP90316. doi: 10.7554/eLife.90316 (PMC11139479; doi:10.7554/eLife.90316)

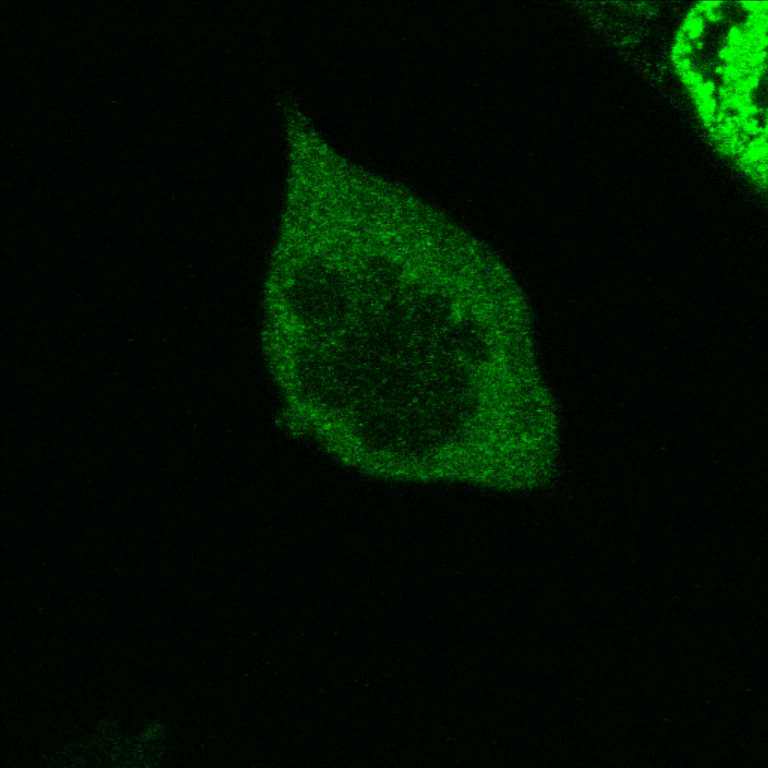

Supplement: Figure 5—figure supplement 1—source data 1. [file elife-90316-fig5-figsupp1-data1.zip › TRMT1-GFP-green.png]

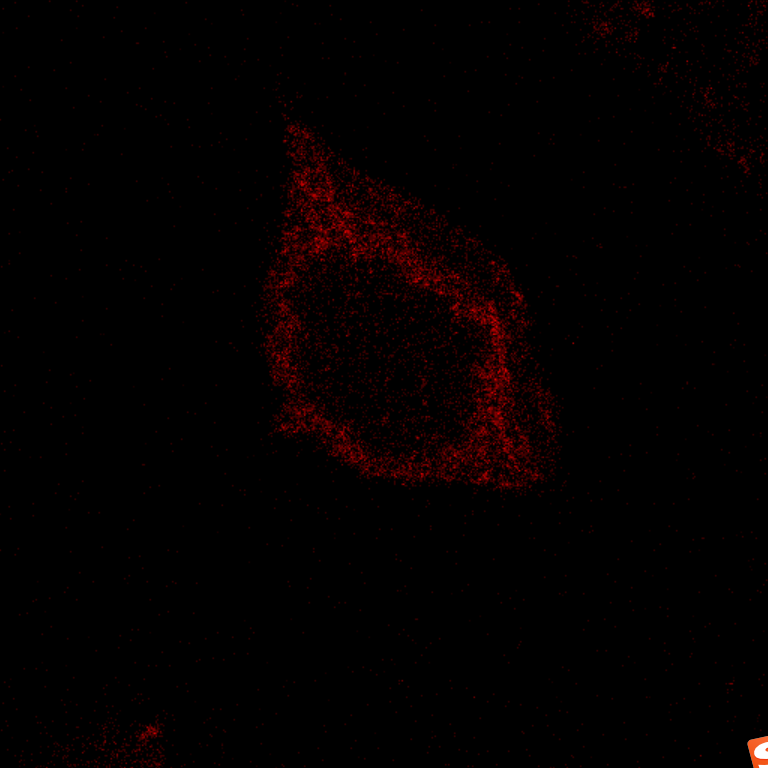

Supplement: Figure 5—figure supplement 1—source data 1. [file elife-90316-fig5-figsupp1-data1.zip › TRMT1-GFP-red.png]

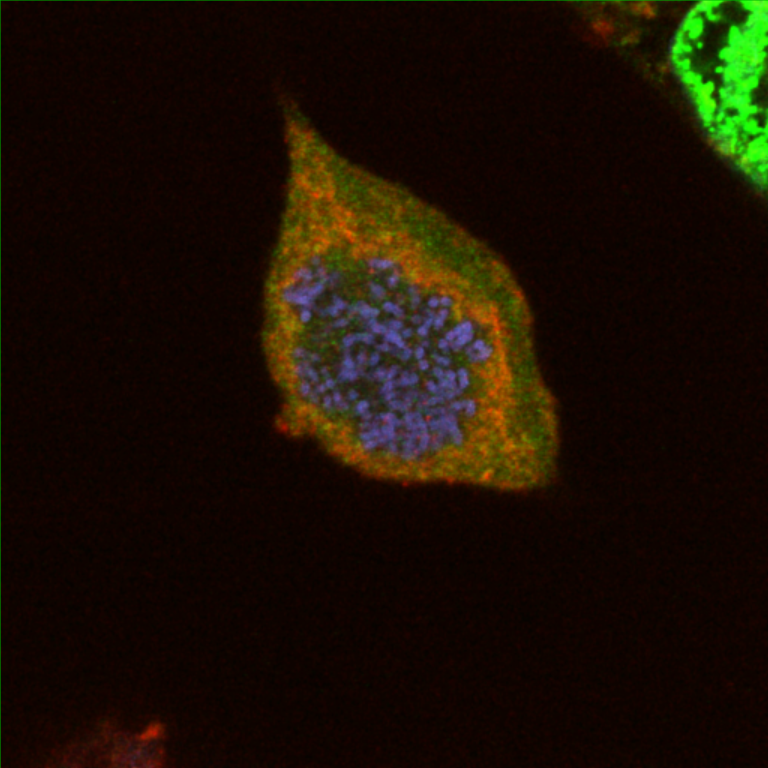

Supplement: Figure 5—figure supplement 1—source data 1. [file elife-90316-fig5-figsupp1-data1.zip › TRMT1-GFP-total.png]

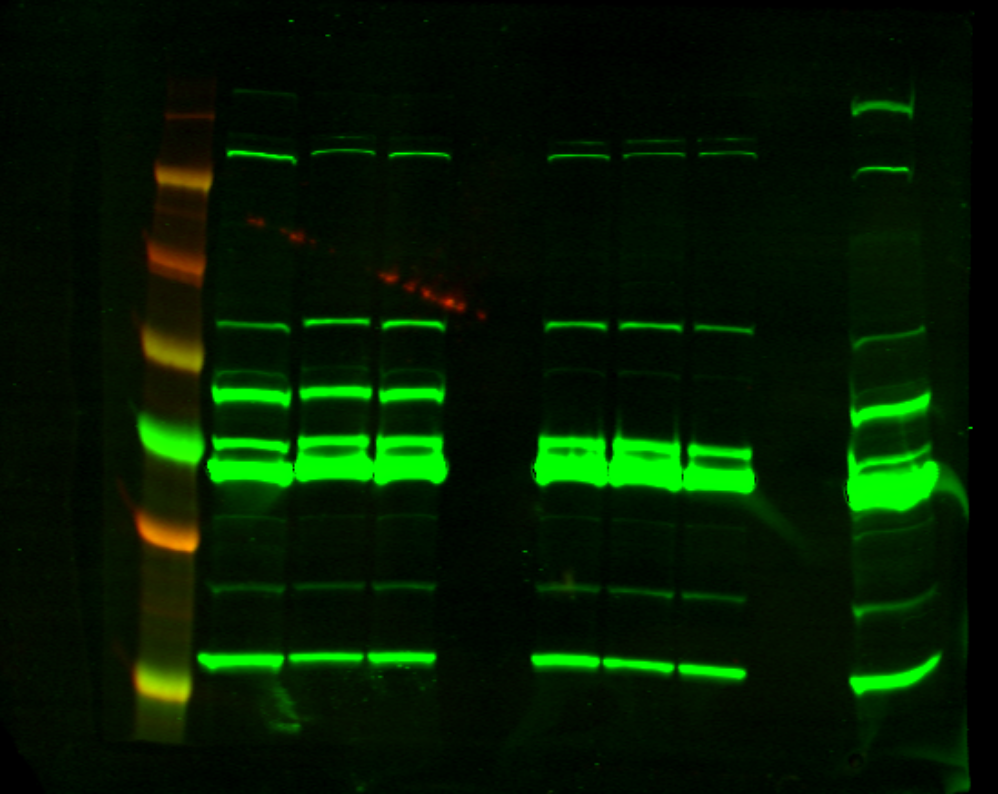

Supplement: Figure 6—source data 1. [file elife-90316-fig6-data1.zip › 6A and B/20220912-TRMT1G3-Ace2 Scr T1KO SARS infection 0 0.2 0.4.tif]

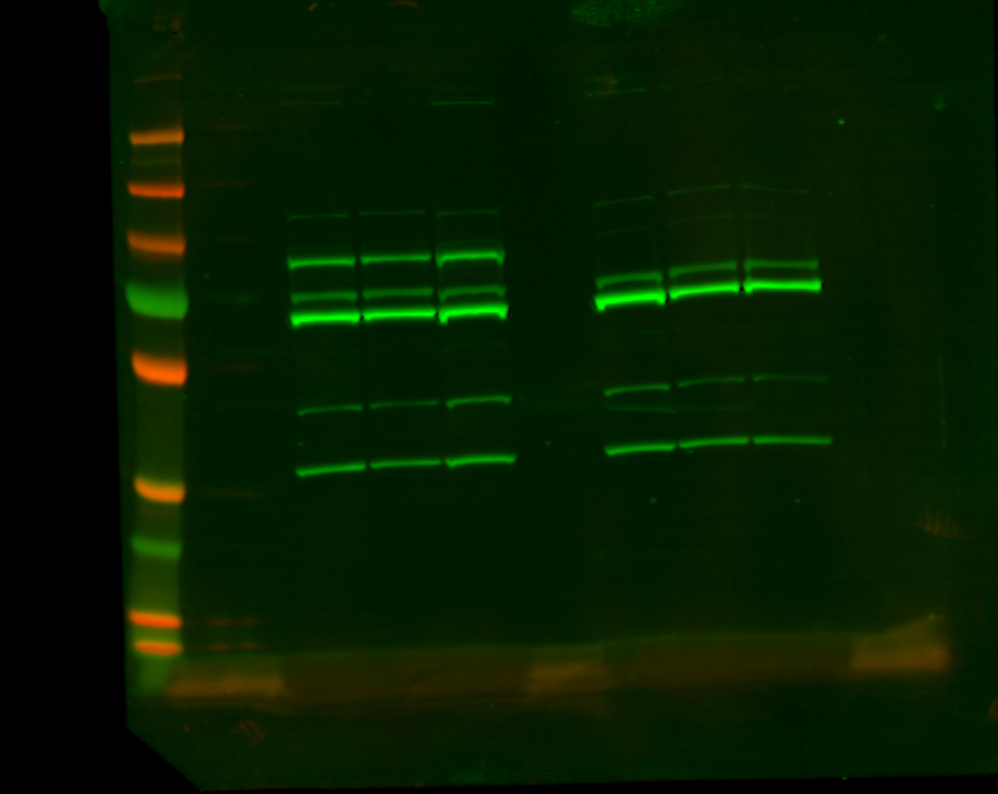

Supplement: Figure 6—source data 1. [file elife-90316-fig6-data1.zip › 6A and B/20230126-TRMT1-G3-293TScr KO 0 0.2 0.4SARS0908.tif]

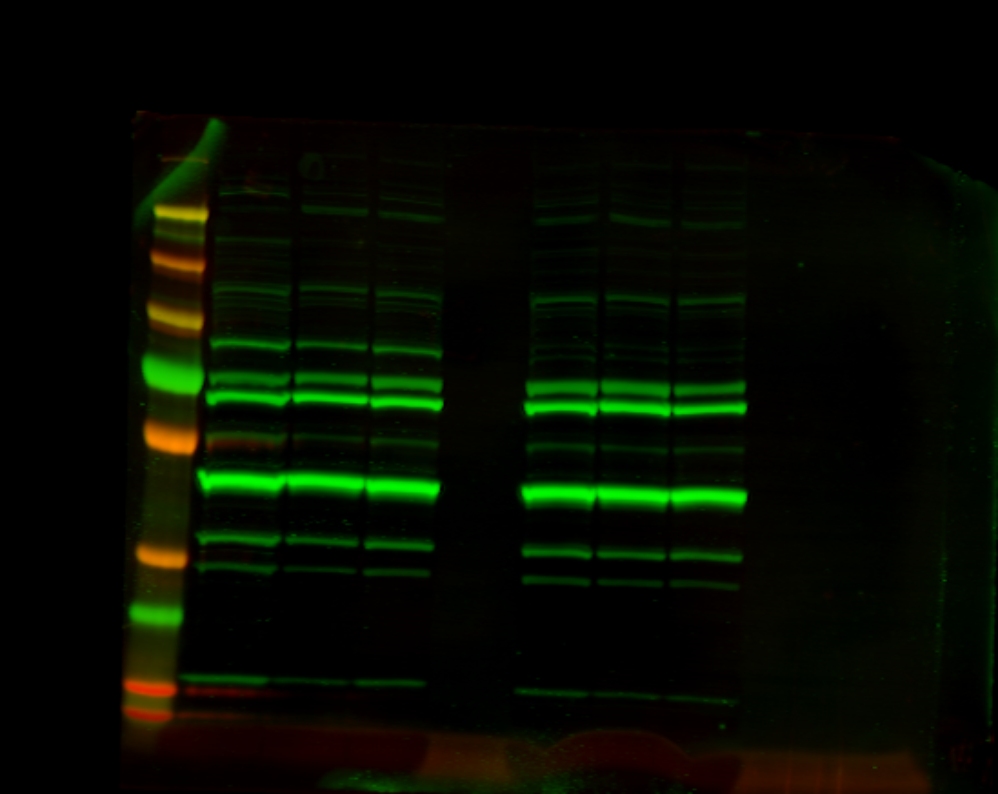

Supplement: Figure 6—source data 1. [file elife-90316-fig6-data1.zip › 6A and B/Figure 6A 20230129-Actin-293TScr KO 0 0.2 0.4SARS0826.tif]

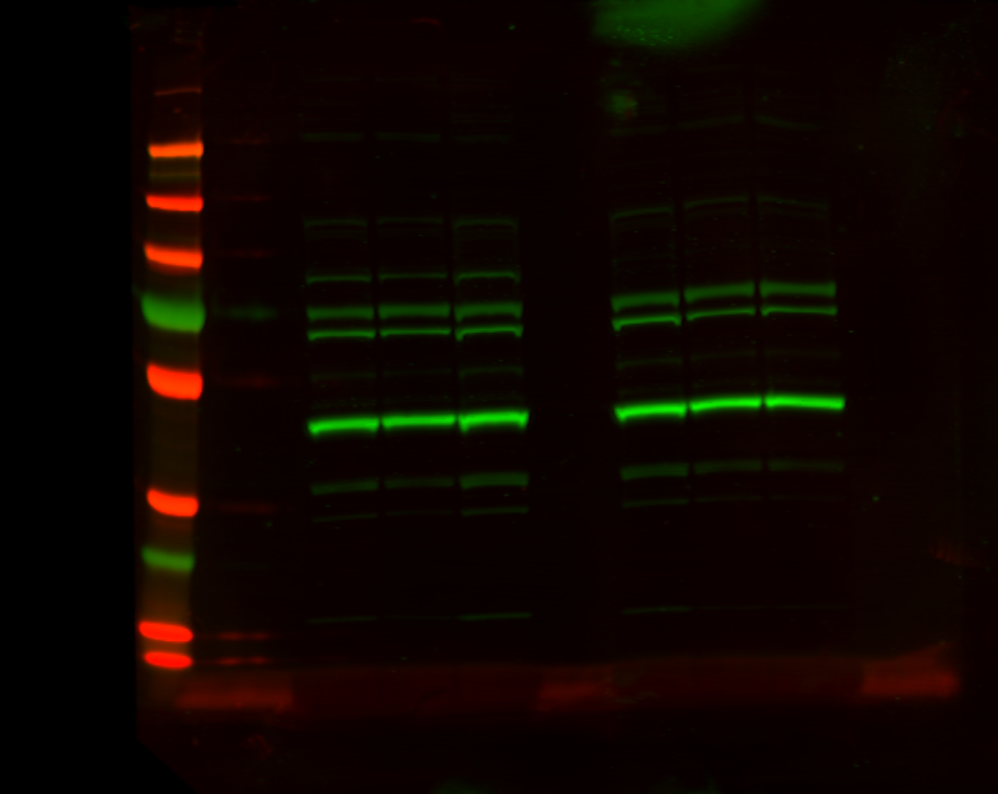

Supplement: Figure 6—source data 1. [file elife-90316-fig6-data1.zip › 6A and B/20230129-Actin-293TScr KO 0 0.2 0.4SARS0908.tif]

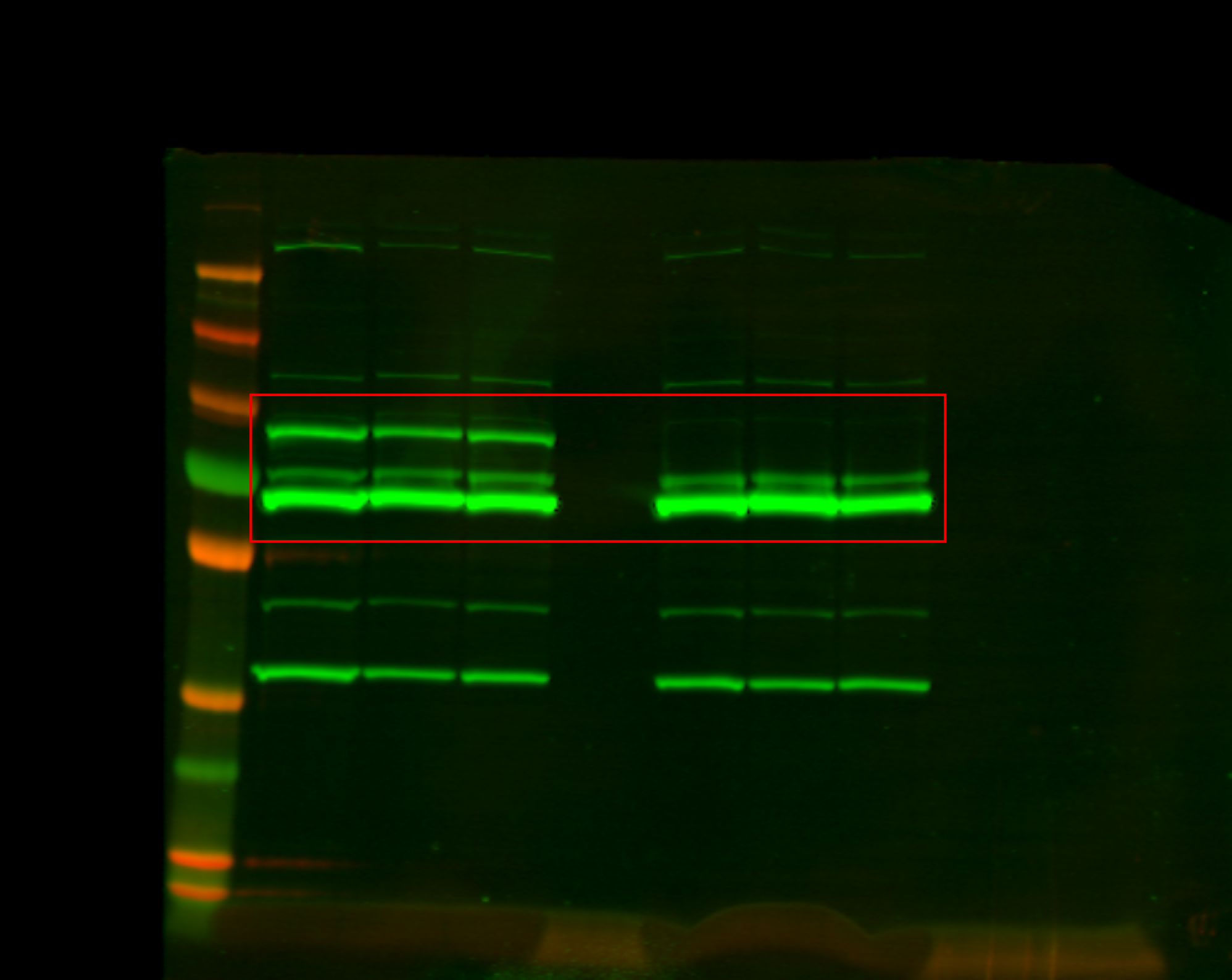

Supplement: Figure 6—source data 1. [file elife-90316-fig6-data1.zip › 6A and B/Figure 6A 20230126-TRMT1-G3-293TScr KO 0 0.2 0.4SARS0826 labeled.tif]

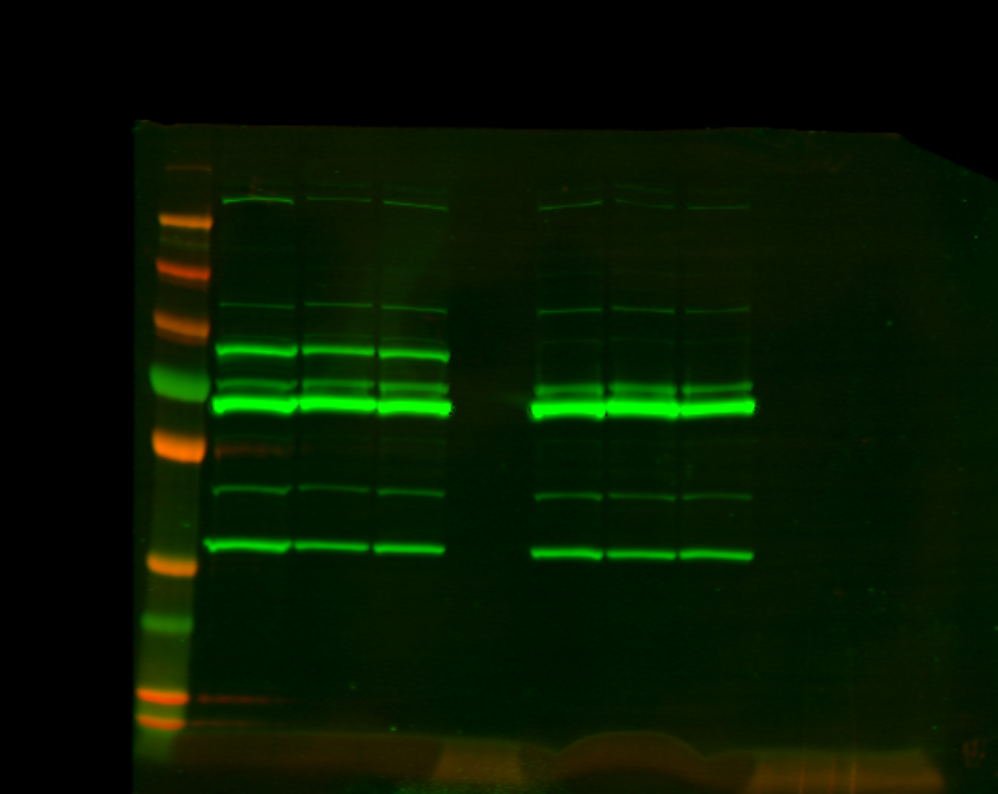

Supplement: Figure 6—source data 1. [file elife-90316-fig6-data1.zip › 6A and B/Figure 6A 20230126-TRMT1-G3-293TScr KO 0 0.2 0.4SARS0826.tif]

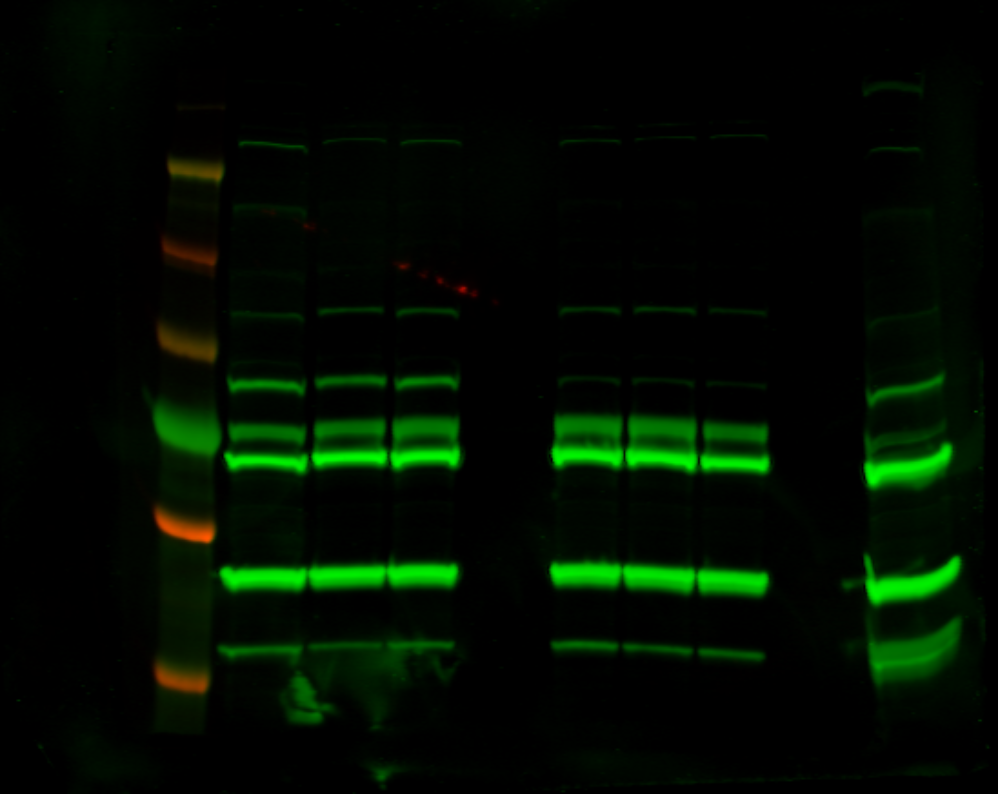

Supplement: Figure 6—source data 1. [file elife-90316-fig6-data1.zip › 6A and B/20220913-Actin-Ace2 Scr T1KO SARS infection 0 0.2 0.4.tif]

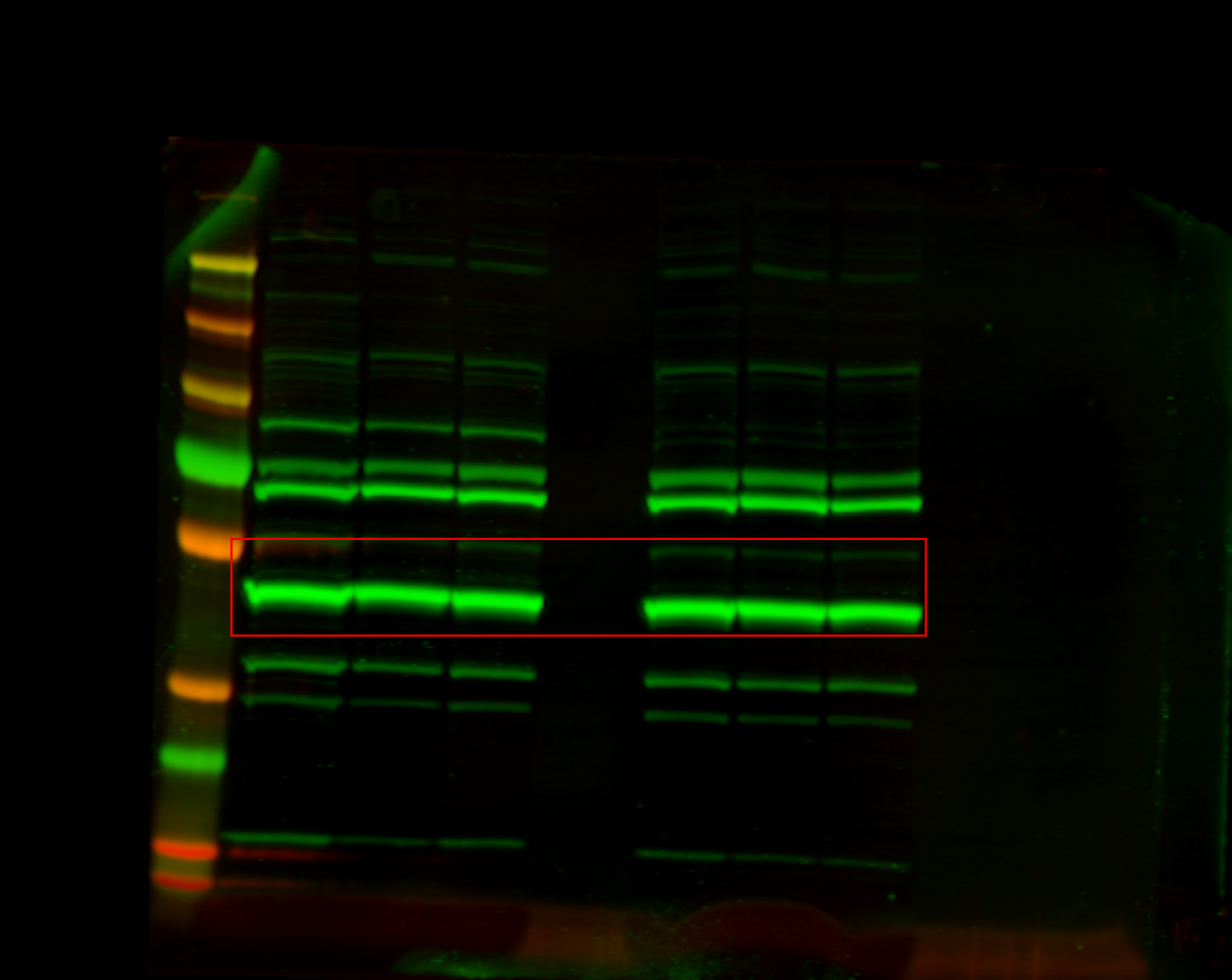

Supplement: Figure 6—source data 1. [file elife-90316-fig6-data1.zip › 6A and B/Figure 6A 20230129-Actin-293TScr KO 0 0.2 0.4SARS0826 labeled.tif]

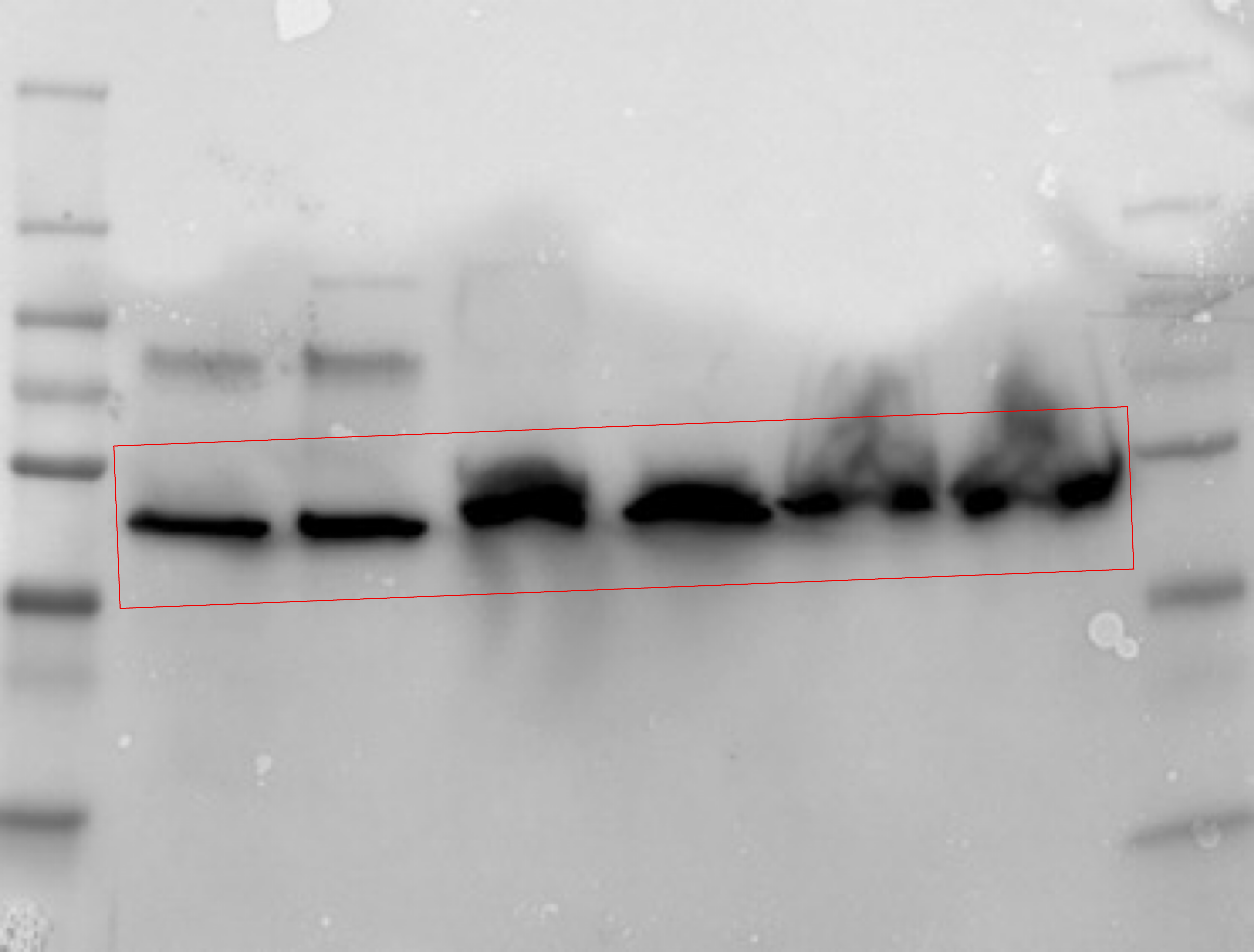

Supplement: Figure 6—source data 1. [file elife-90316-fig6-data1.zip › 6C and D/Figure 6C actin labeled.tif]

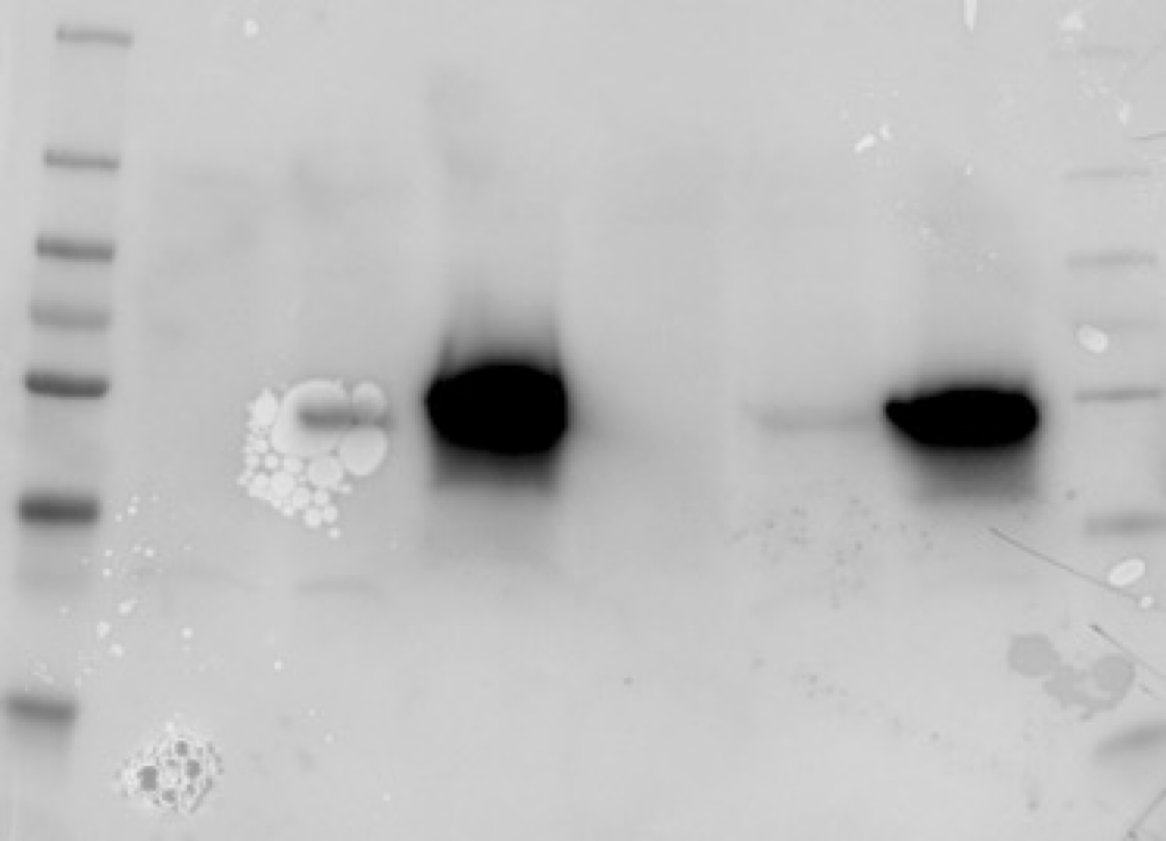

Supplement: Figure 6—source data 1. [file elife-90316-fig6-data1.zip › 6C and D/Figure 6C N protein.tiff]

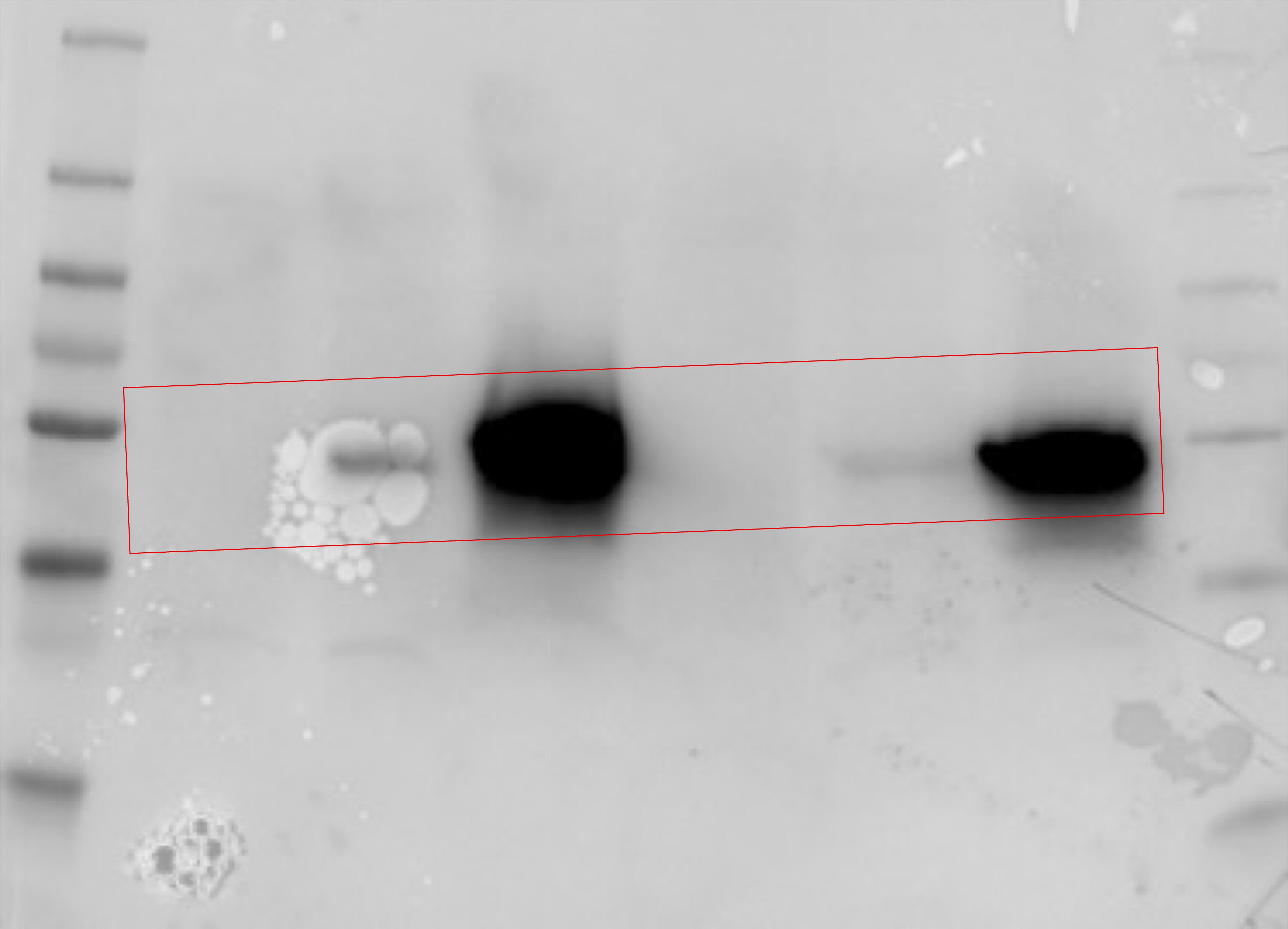

Supplement: Figure 6—source data 1. [file elife-90316-fig6-data1.zip › 6C and D/Figure 6C N protein labeled.tif]

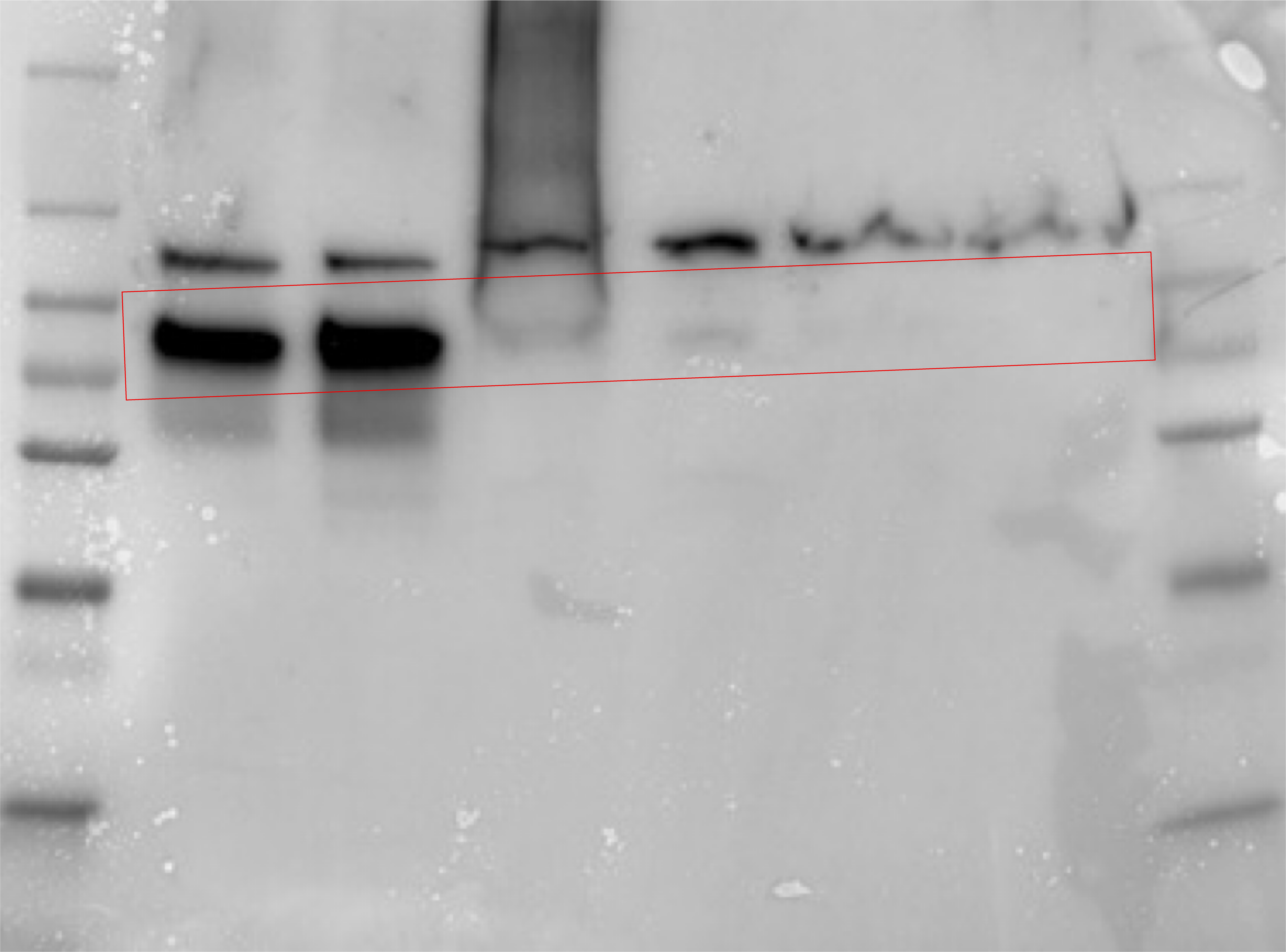

Supplement: Figure 6—source data 1. [file elife-90316-fig6-data1.zip › 6C and D/Figure 6C TRMT1 labeled.tif]

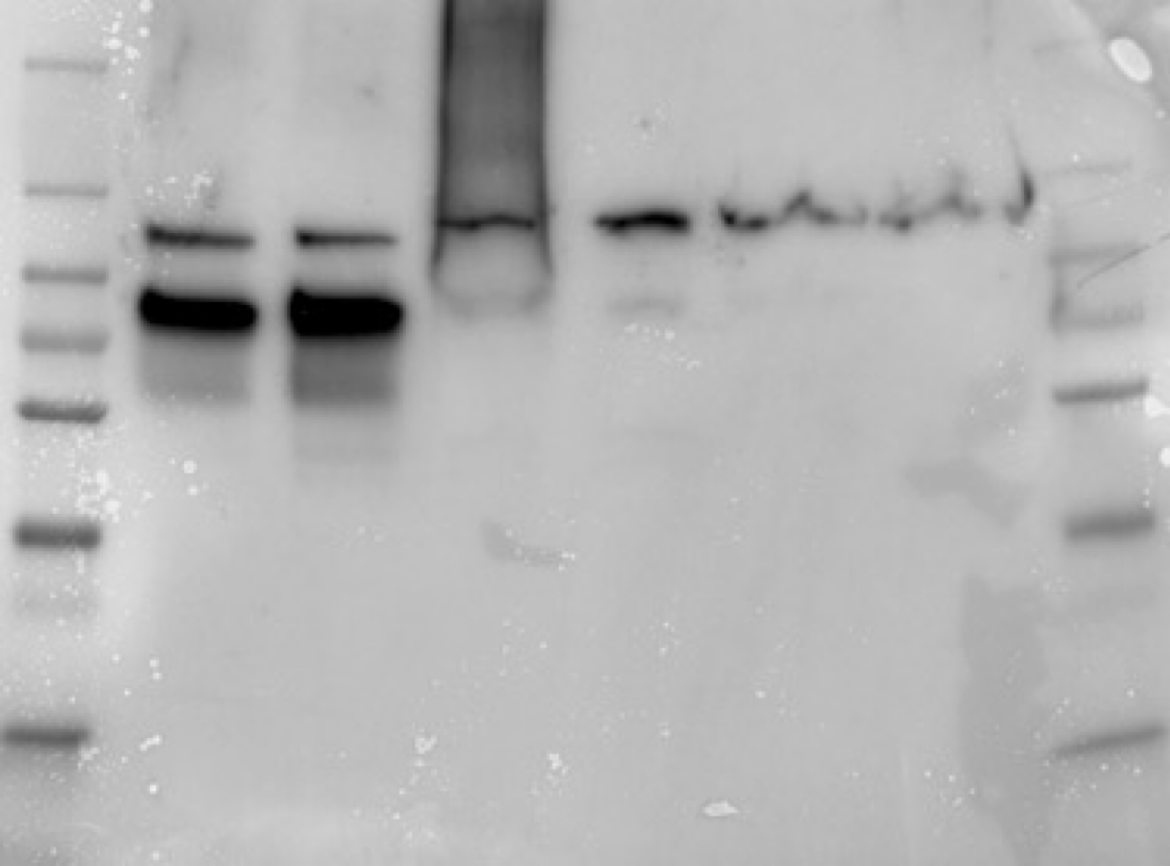

Supplement: Figure 6—source data 1. [file elife-90316-fig6-data1.zip › 6C and D/Figure 6C TRMT1.tiff]

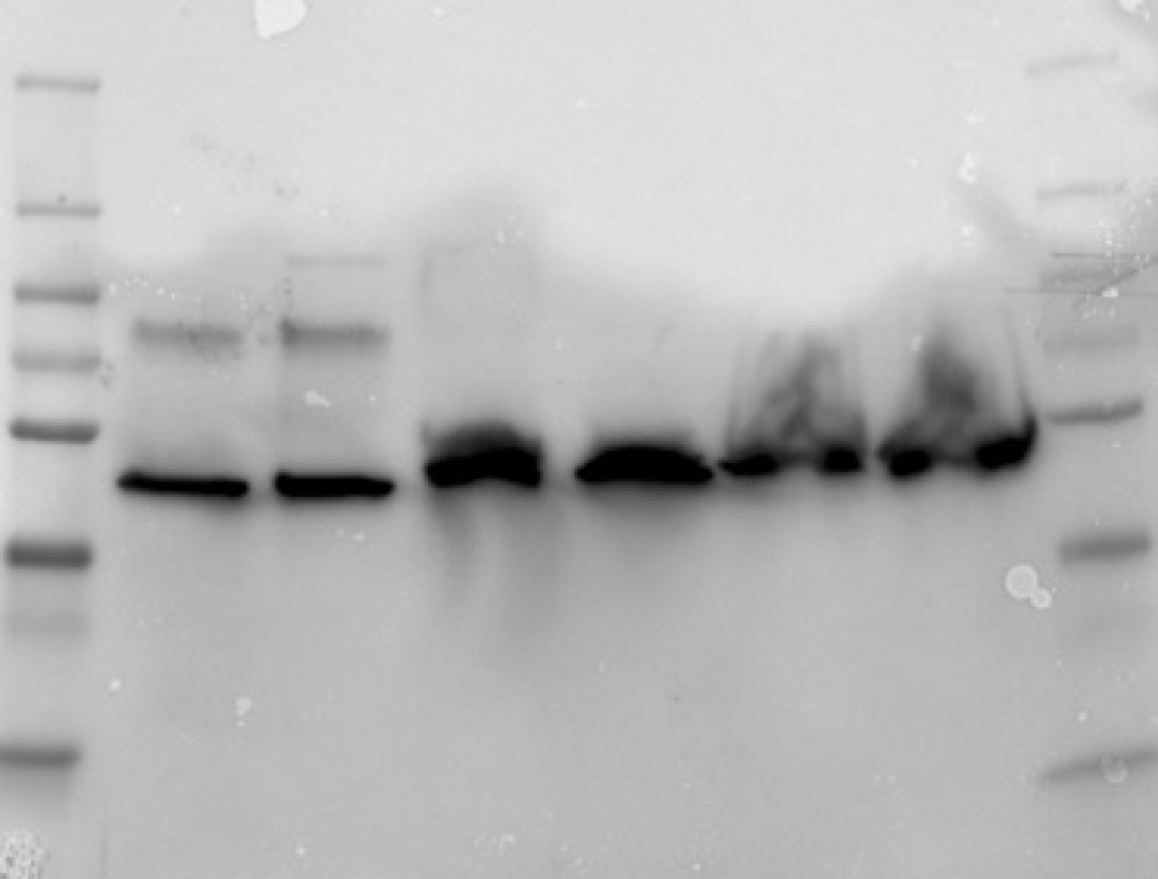

Supplement: Figure 6—source data 1. [file elife-90316-fig6-data1.zip › 6C and D/Figure 6C actin.tiff]

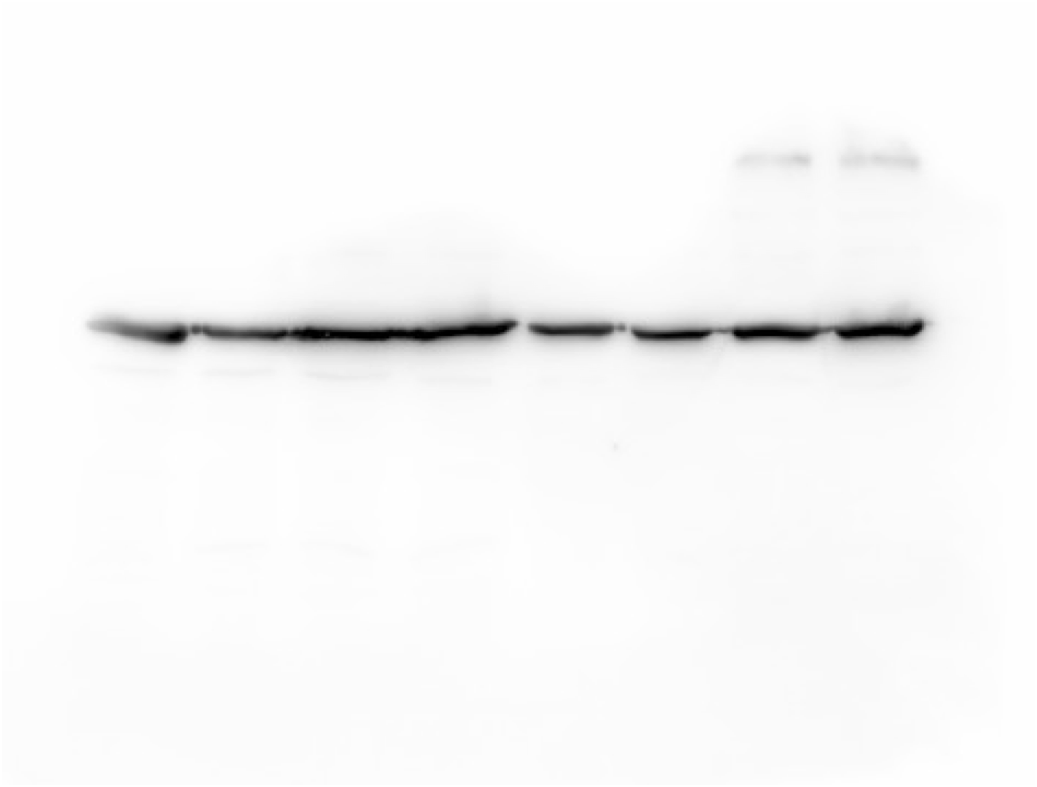

Supplement: Figure 6—figure supplement 1—source data 1. [file elife-90316-fig6-figsupp1-data1.zip › S6A/SFigure 4 blot actin.tiff]

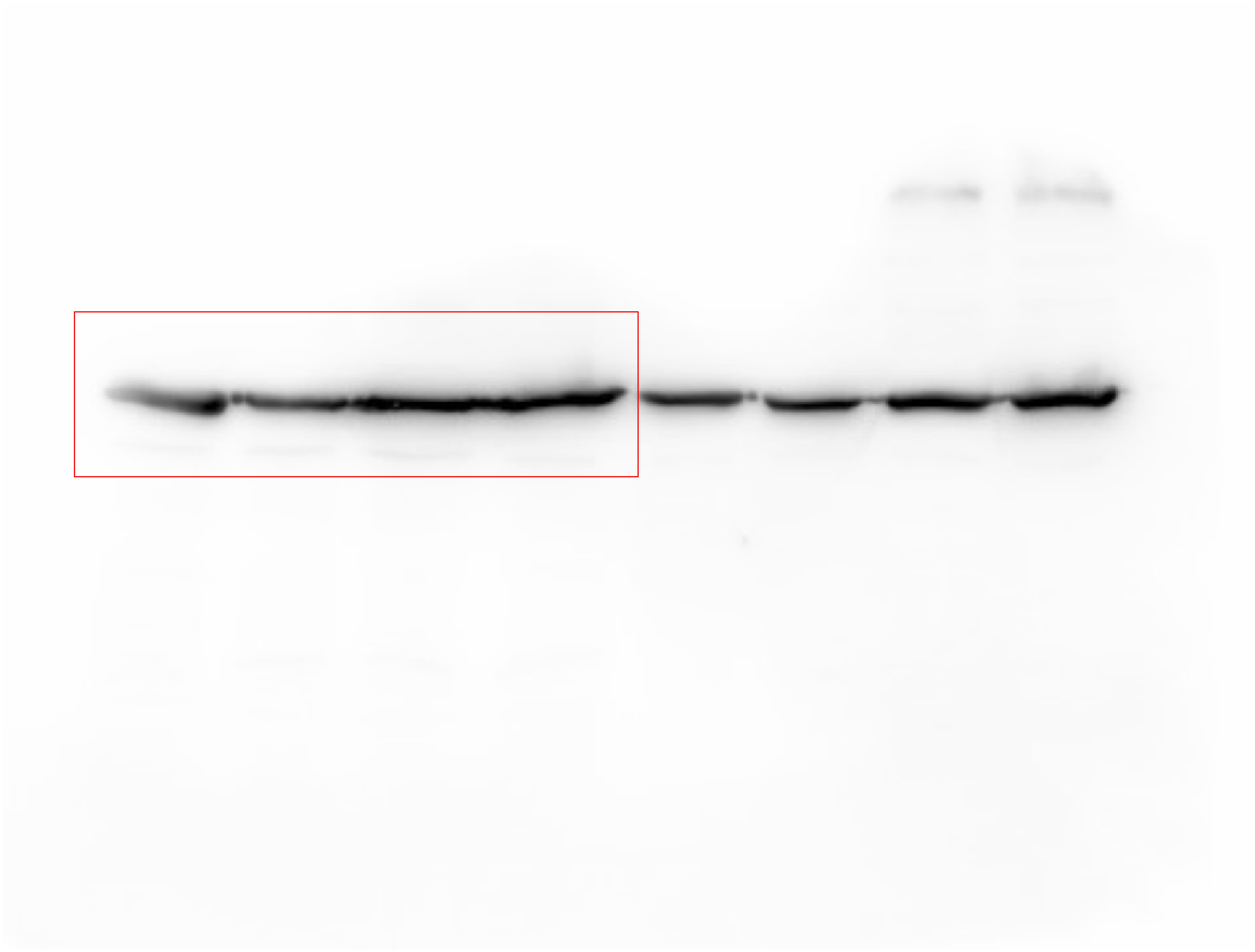

Supplement: Figure 6—figure supplement 1—source data 1. [file elife-90316-fig6-figsupp1-data1.zip › S6A/SFigure 4 blot actin labeled.tif]

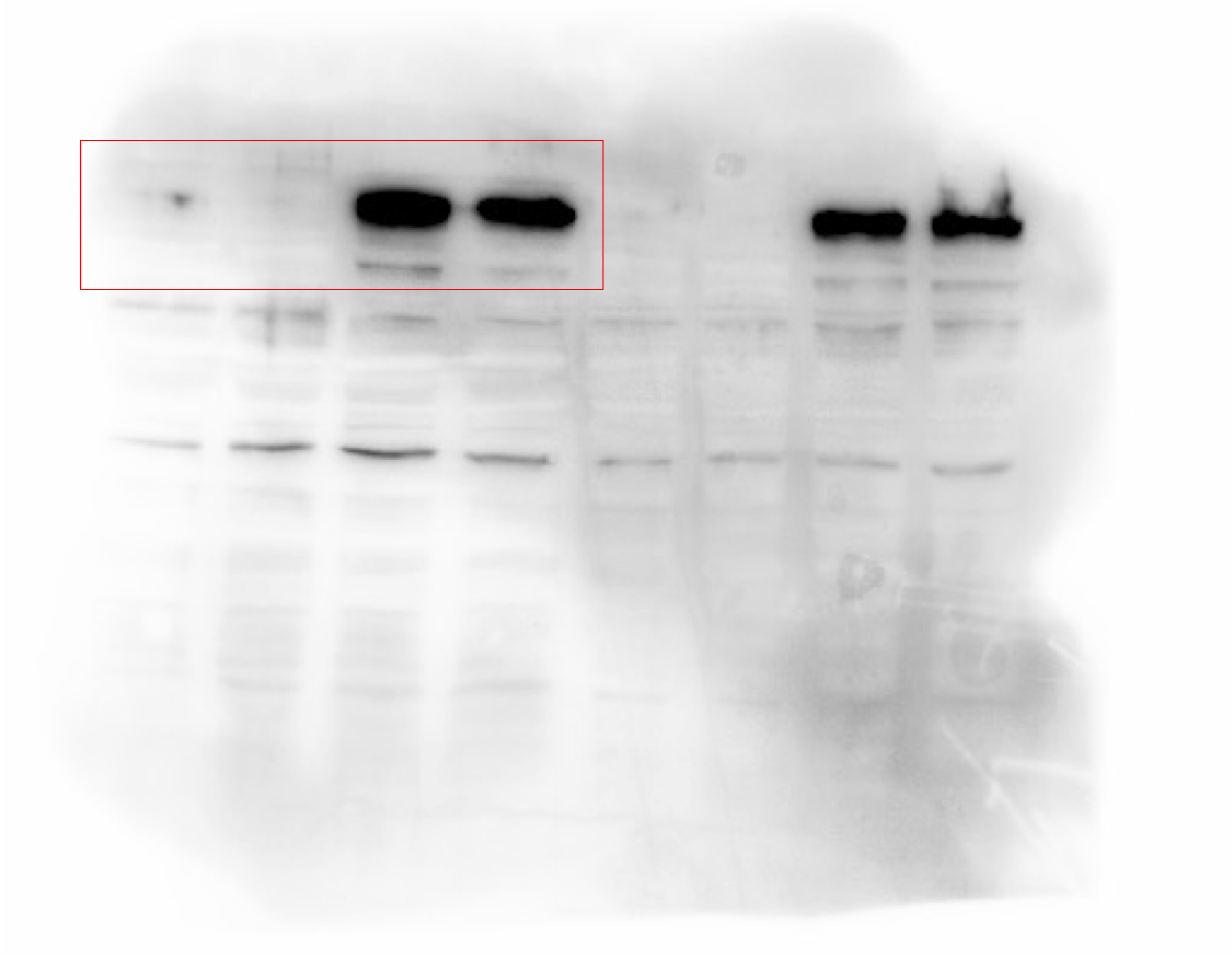

Supplement: Figure 6—figure supplement 1—source data 1. [file elife-90316-fig6-figsupp1-data1.zip › S6A/SFigure 4 blot ACE2 labeled.tif]

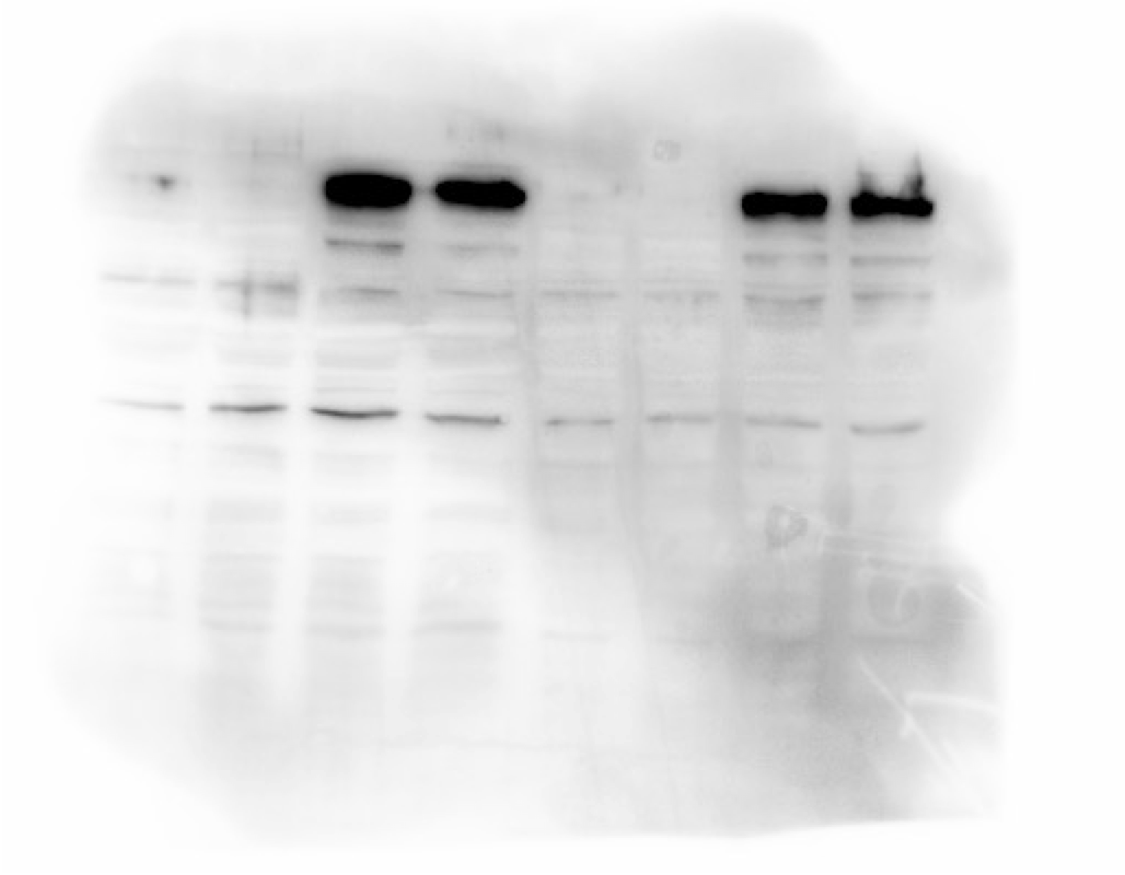

Supplement: Figure 6—figure supplement 1—source data 1. [file elife-90316-fig6-figsupp1-data1.zip › S6A/SFigure 4 blot ACE2.tiff]

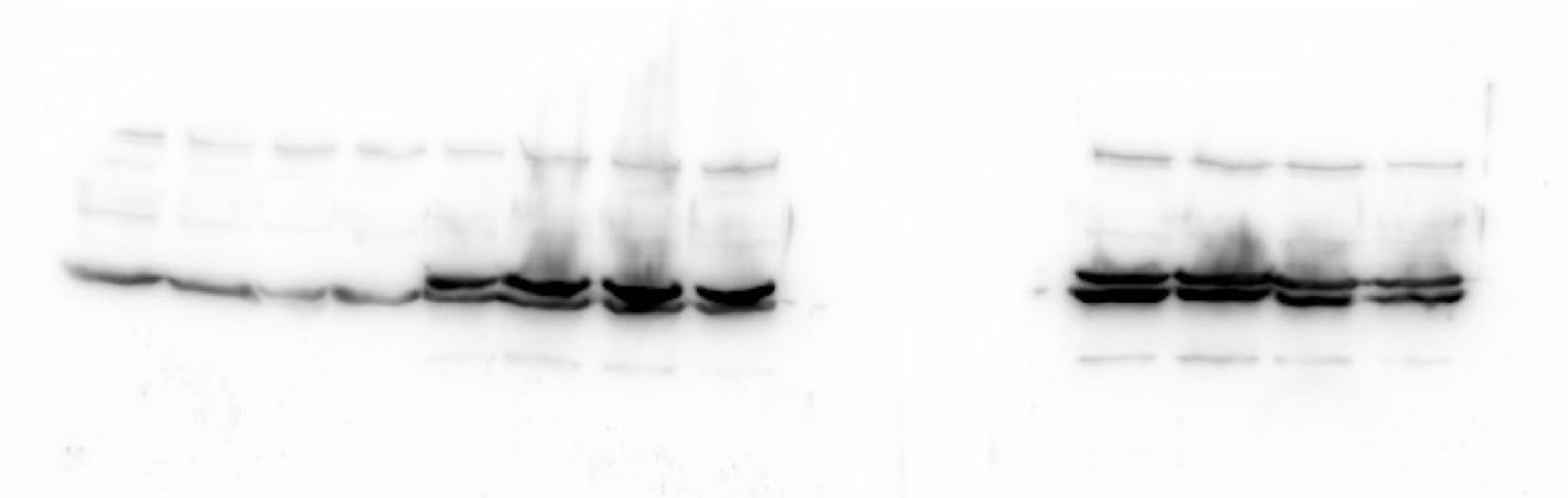

Supplement: Figure 6—figure supplement 1—source data 1. [file elife-90316-fig6-figsupp1-data1.zip › S6B/4B nucleocapsid and actin.tiff]

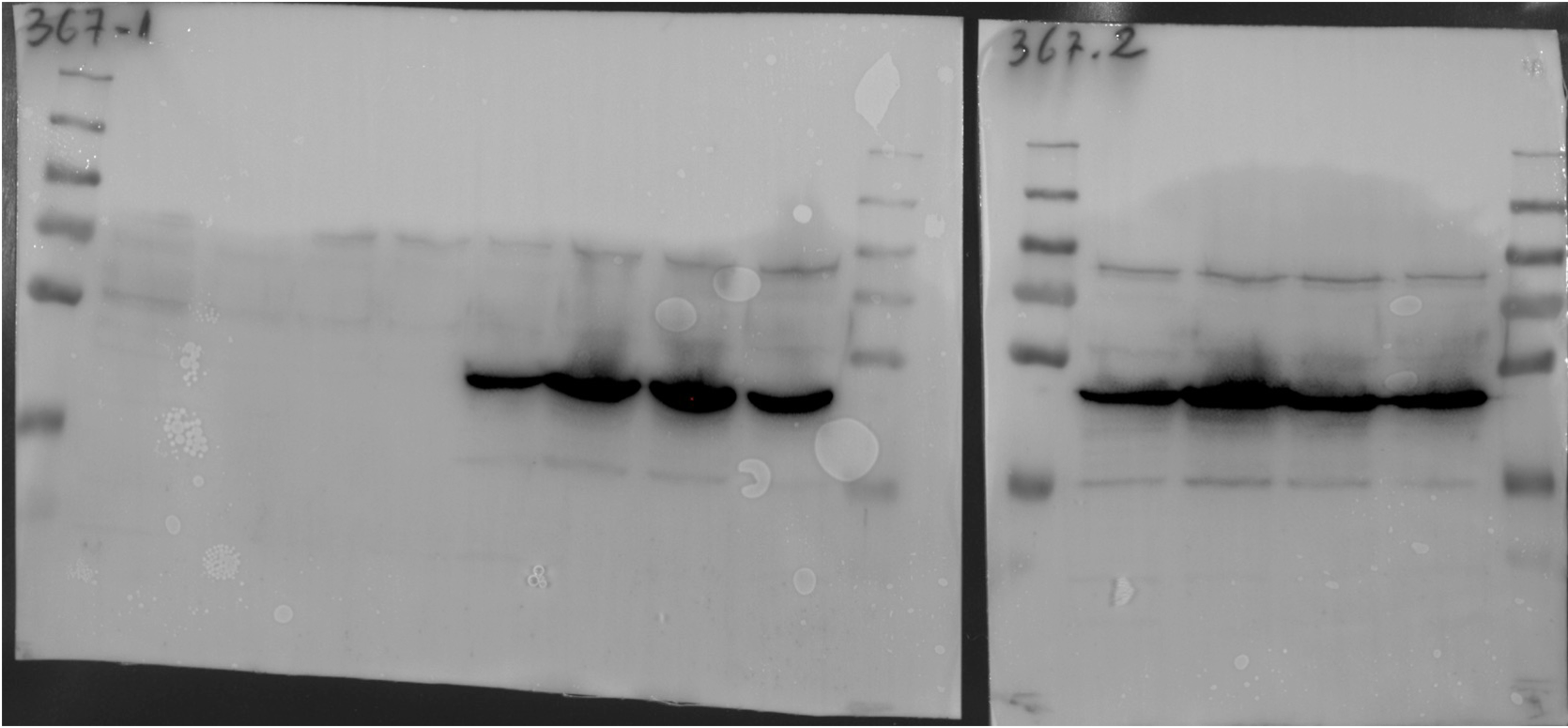

Supplement: Figure 6—figure supplement 1—source data 1. [file elife-90316-fig6-figsupp1-data1.zip › S6B/4B blot N protein.tiff]

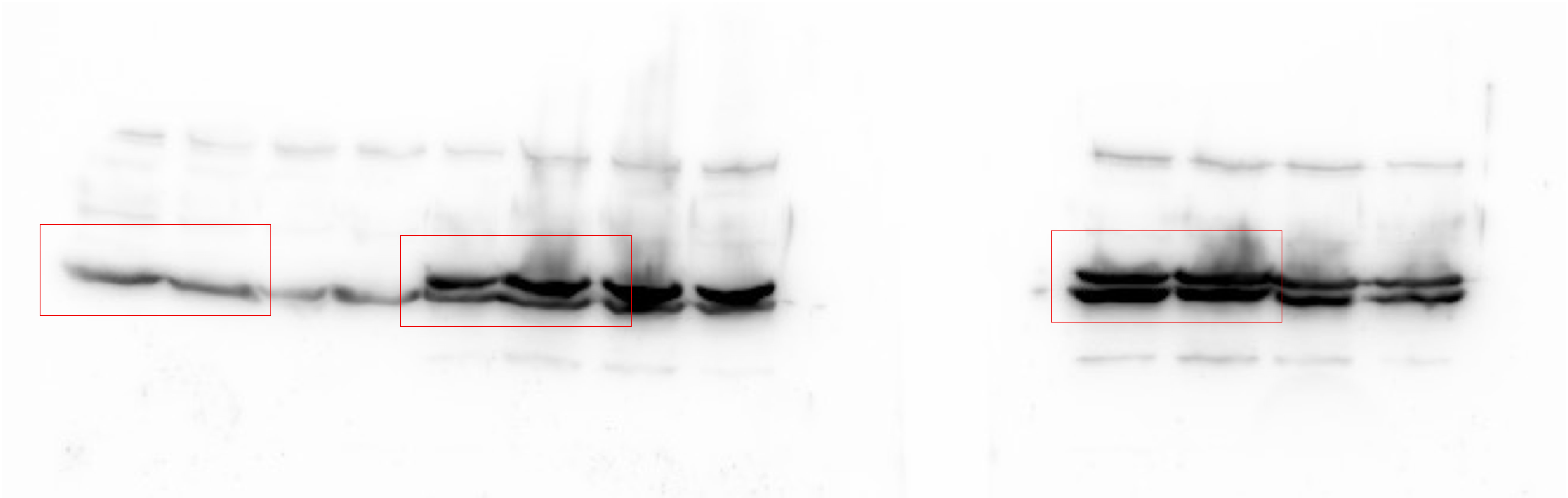

Supplement: Figure 6—figure supplement 1—source data 1. [file elife-90316-fig6-figsupp1-data1.zip › S6B/4B nucleocapsid and actin labeled.tif]

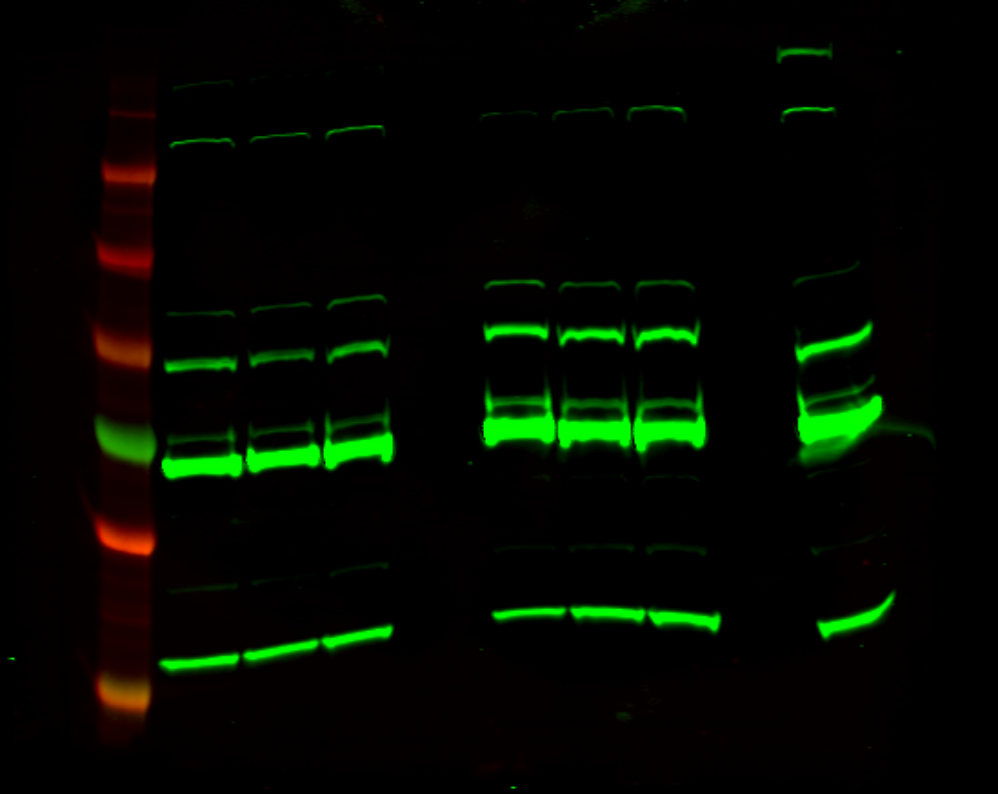

Supplement: Figure 7—source data 1. [file elife-90316-fig7-data1.zip › 20220912-TRMT1G3-Ace2 WT-flag Q530N-flag SARS infection 0 0.2 0.4.tif]

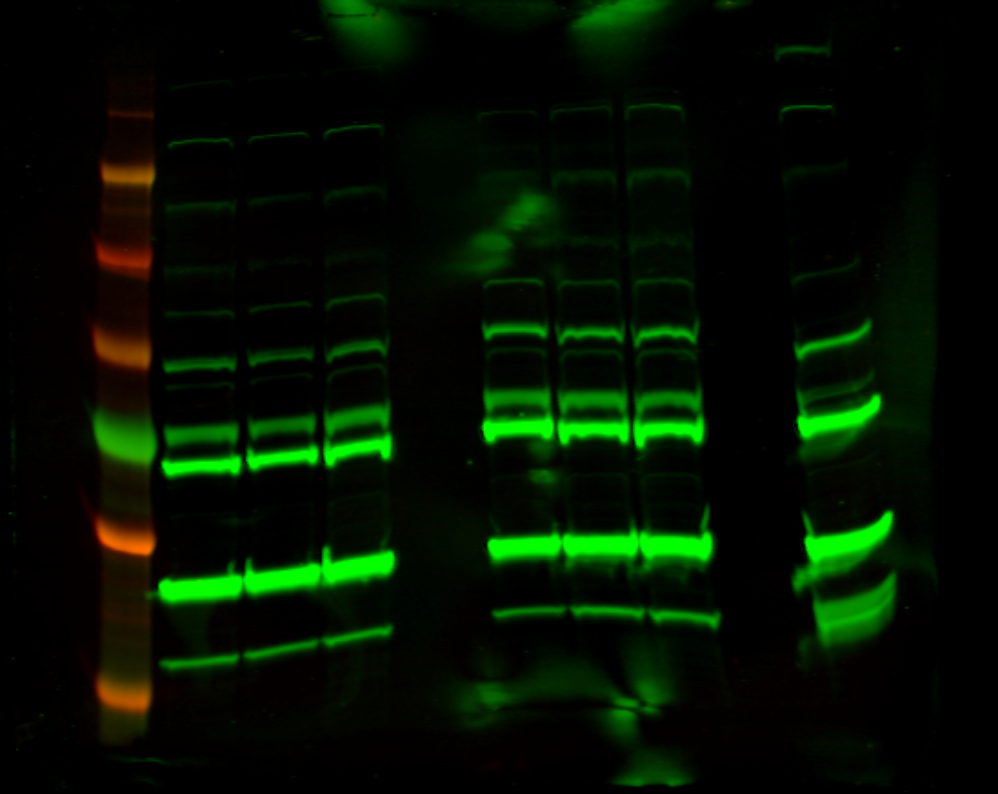

Supplement: Figure 7—source data 1. [file elife-90316-fig7-data1.zip › 20220913-Actin-Ace2 WT-flag Q530N-flag SARS infection 0 0.2 0.4.tif]

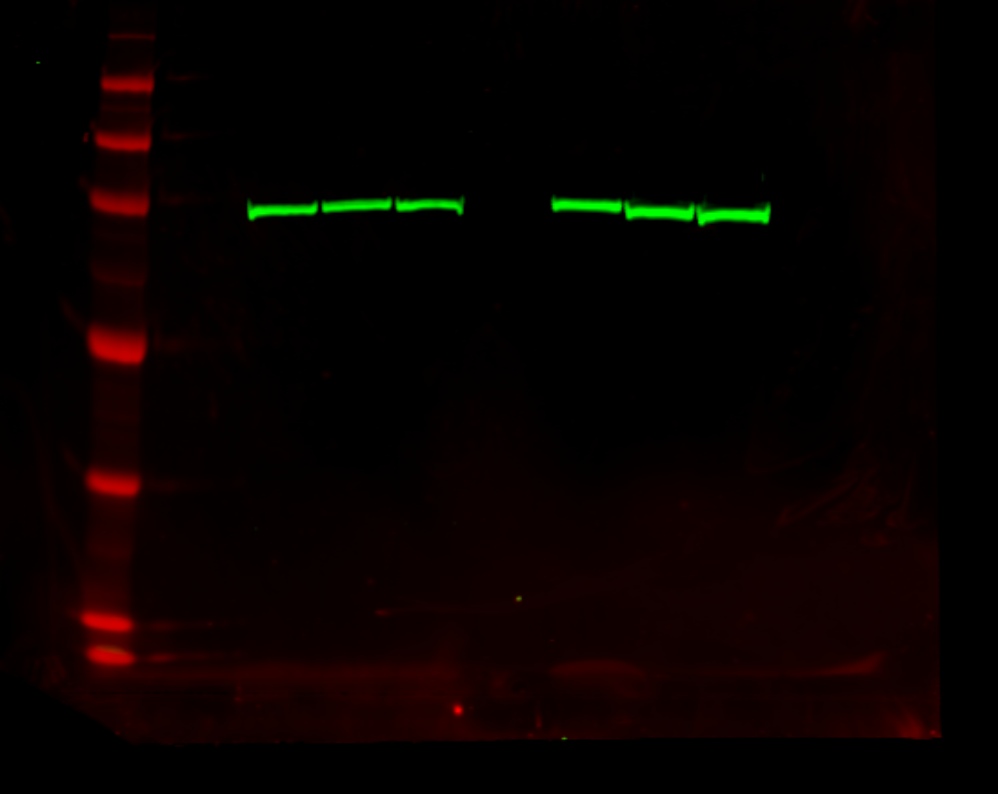

Supplement: Figure 7—source data 1. [file elife-90316-fig7-data1.zip › 20230624-flag-SARS infect 0 0.2 0.4 Flag-WT Flag-Q530N0826.tif]

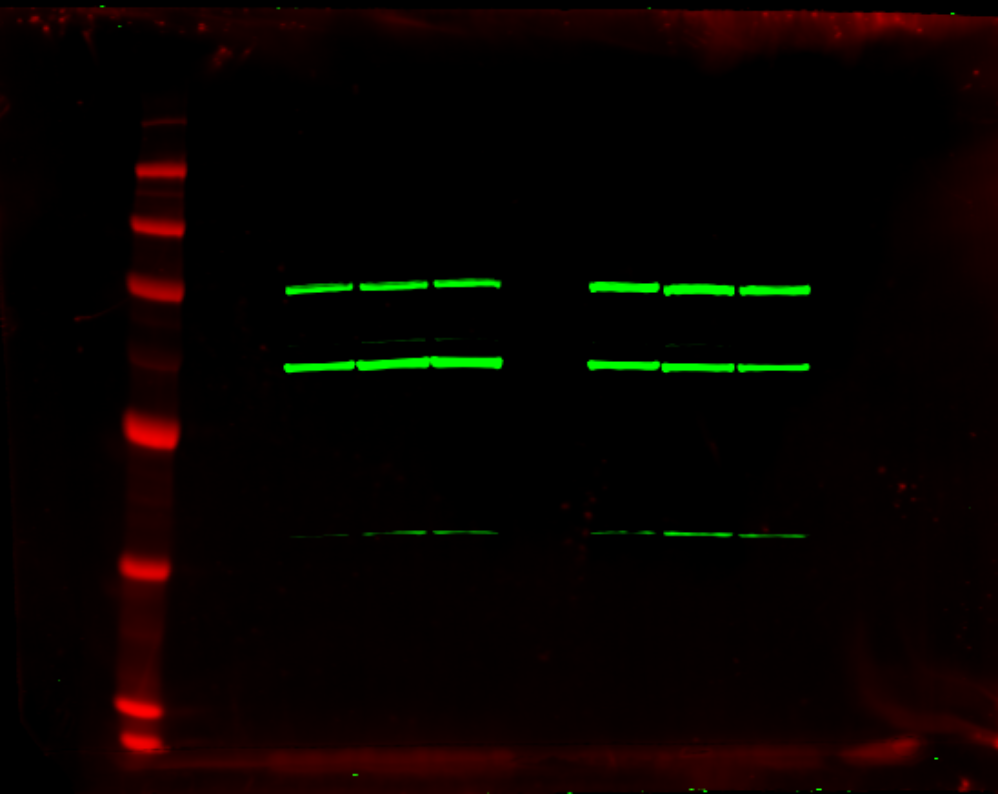

Supplement: Figure 7—source data 1. [file elife-90316-fig7-data1.zip › 20230624-TRMT1G3-SARS infect 0 0.2 0.4 Flag-WT Flag-Q530N0908.tif]

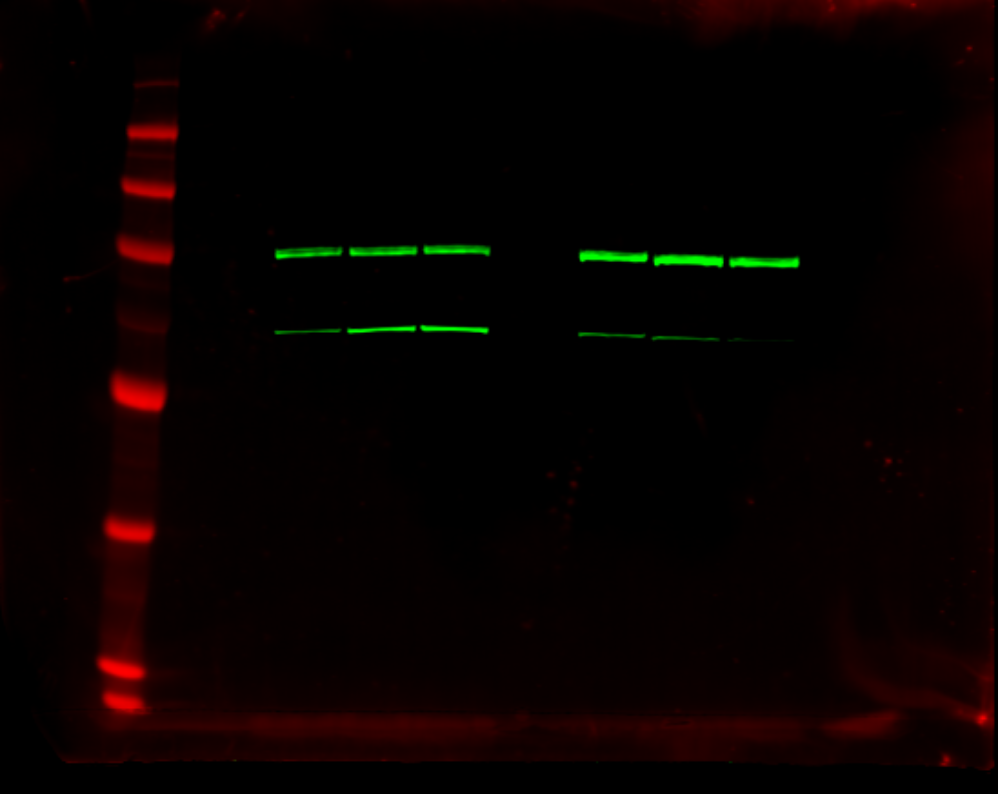

Supplement: Figure 7—source data 1. [file elife-90316-fig7-data1.zip › 20230626-flag-SARS infect 0 0.2 0.4 Flag-WT Flag-Q530N0908.tif]

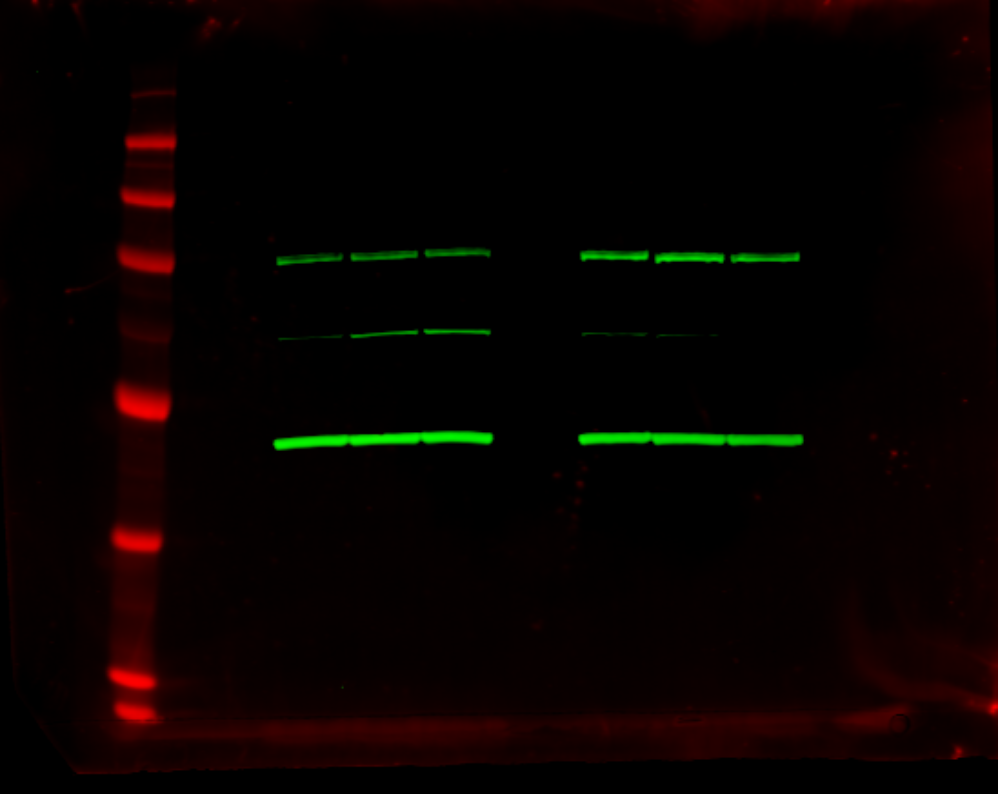

Supplement: Figure 7—source data 1. [file elife-90316-fig7-data1.zip › 20230627-Actin-SARS infect 0 0.2 0.4 Flag-WT Flag-Q530N0908.tif]

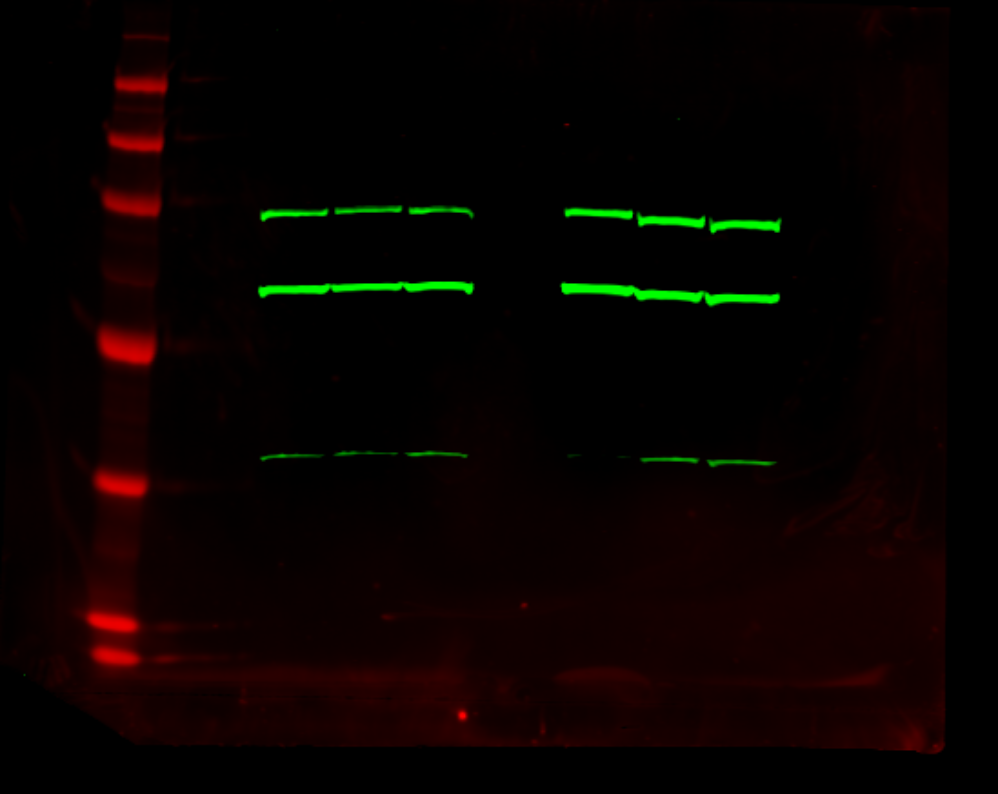

Supplement: Figure 7—source data 1. [file elife-90316-fig7-data1.zip › Figure 7A 20230626-TRMT1G3-SARS infect 0 0.2 0.4 Flag-WT Flag-Q530N0826.tif]

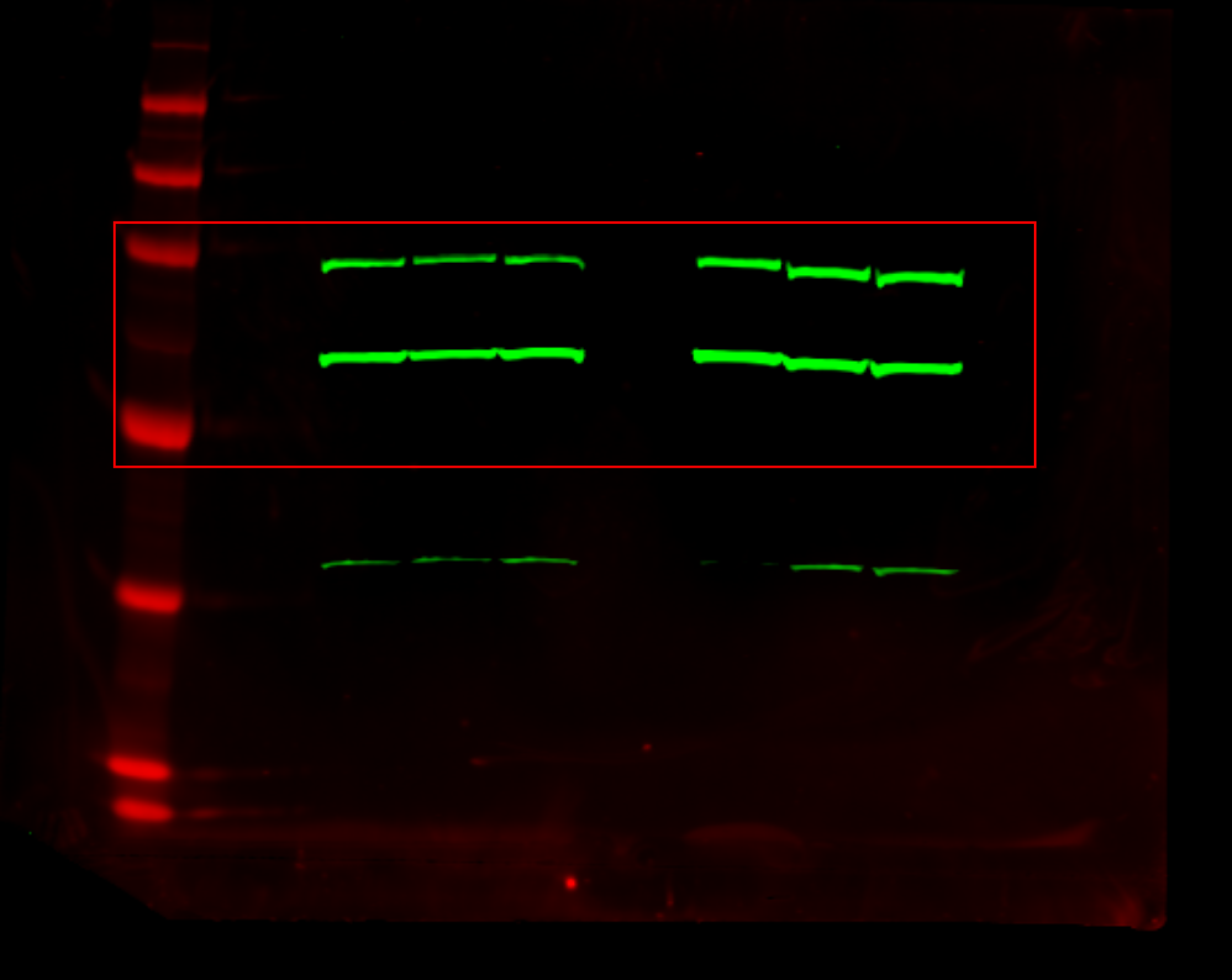

Supplement: Figure 7—source data 1. [file elife-90316-fig7-data1.zip › Figure 7A 20230626-TRMT1G3-SARS infect 0 0.2 0.4 Flag-WT Flag-Q530N0826 labeled.tif]

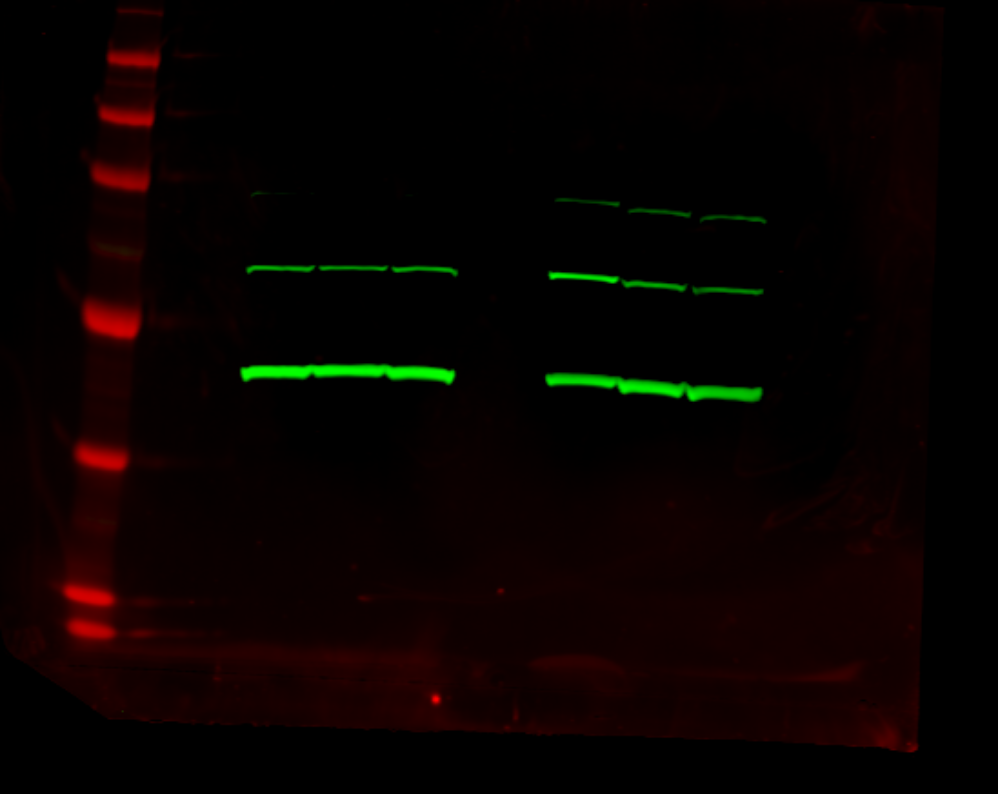

Supplement: Figure 7—source data 1. [file elife-90316-fig7-data1.zip › Figure 7A 20230627-Actin-SARS infect 0 0.2 0.4 Flag-WT Flag-Q530N0826.tif]

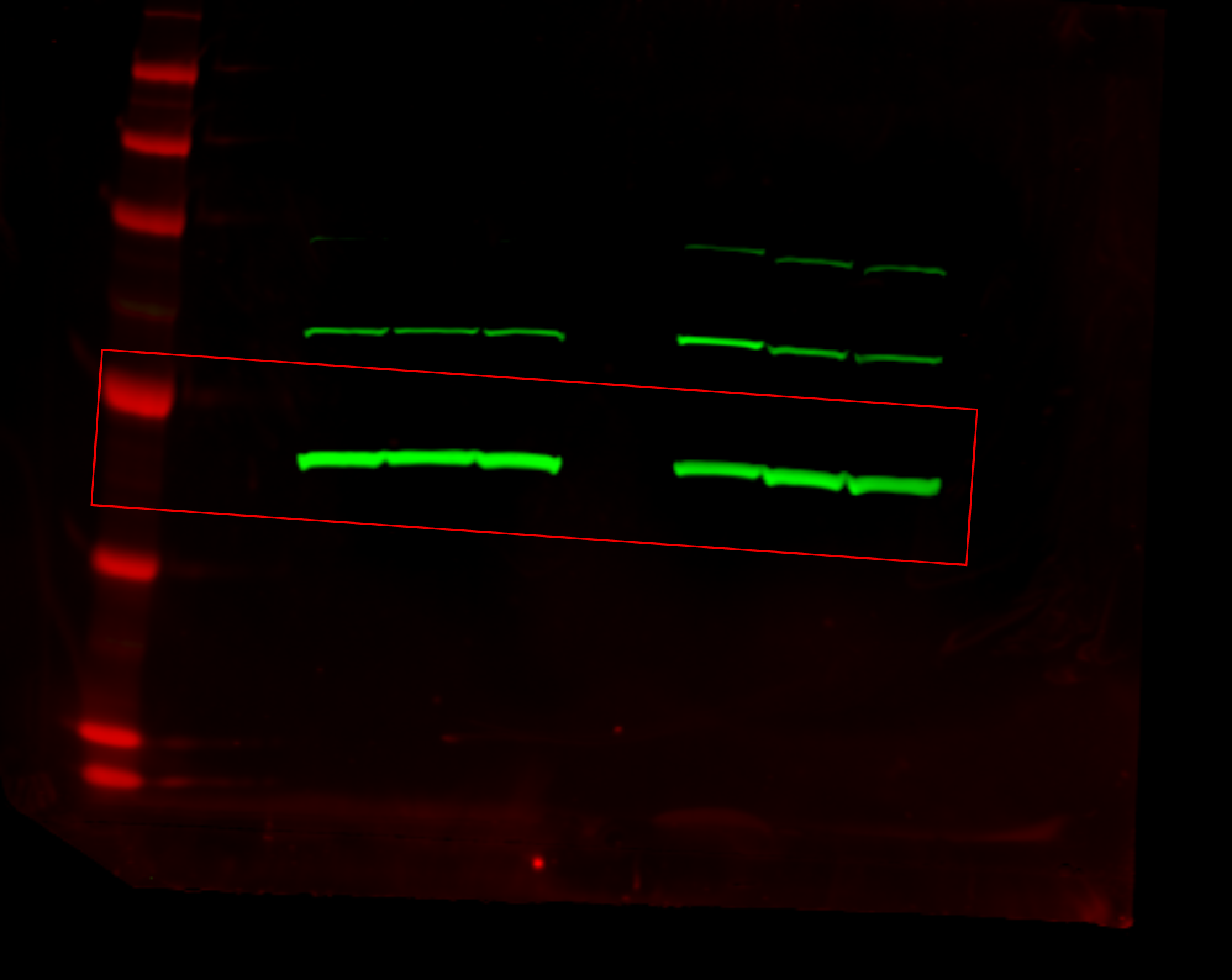

Supplement: Figure 7—source data 1. [file elife-90316-fig7-data1.zip › Figure 7A 20230627-Actin-SARS infect 0 0.2 0.4 Flag-WT Flag-Q530N0826 labeled.tif]

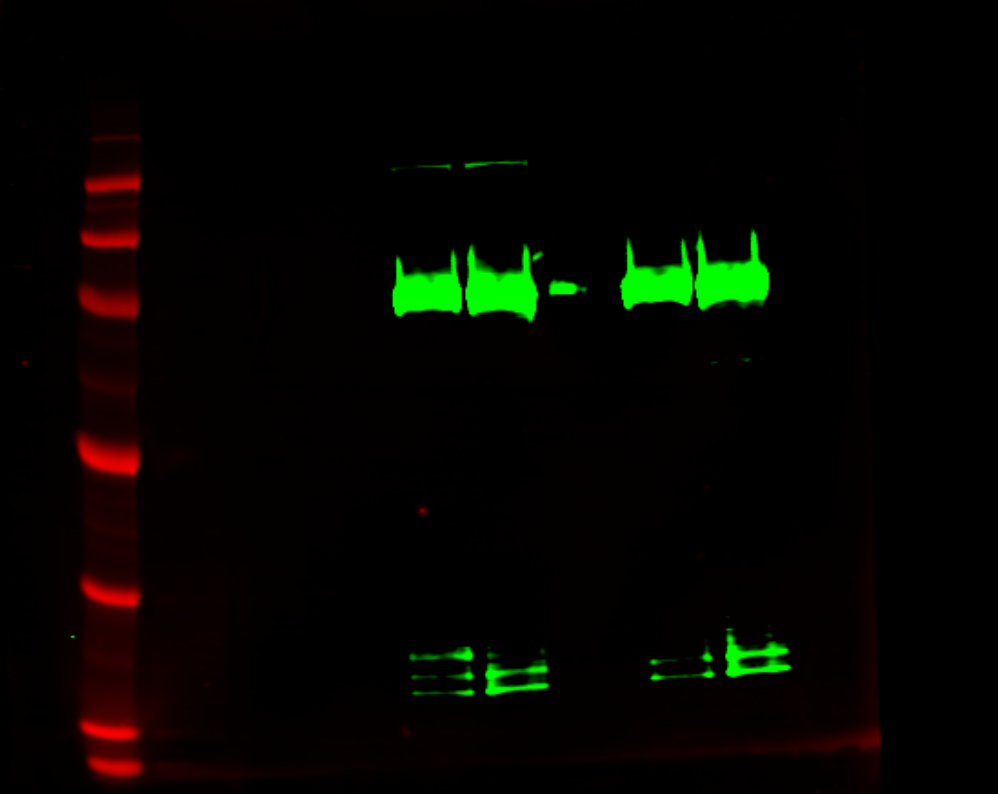

Supplement: Figure 7—figure supplement 1—source data 1. [file elife-90316-fig7-figsupp1-data1.zip › 20220730-Flag-T1KO T1flag Q530Nflag NSP5 transfection.tif]

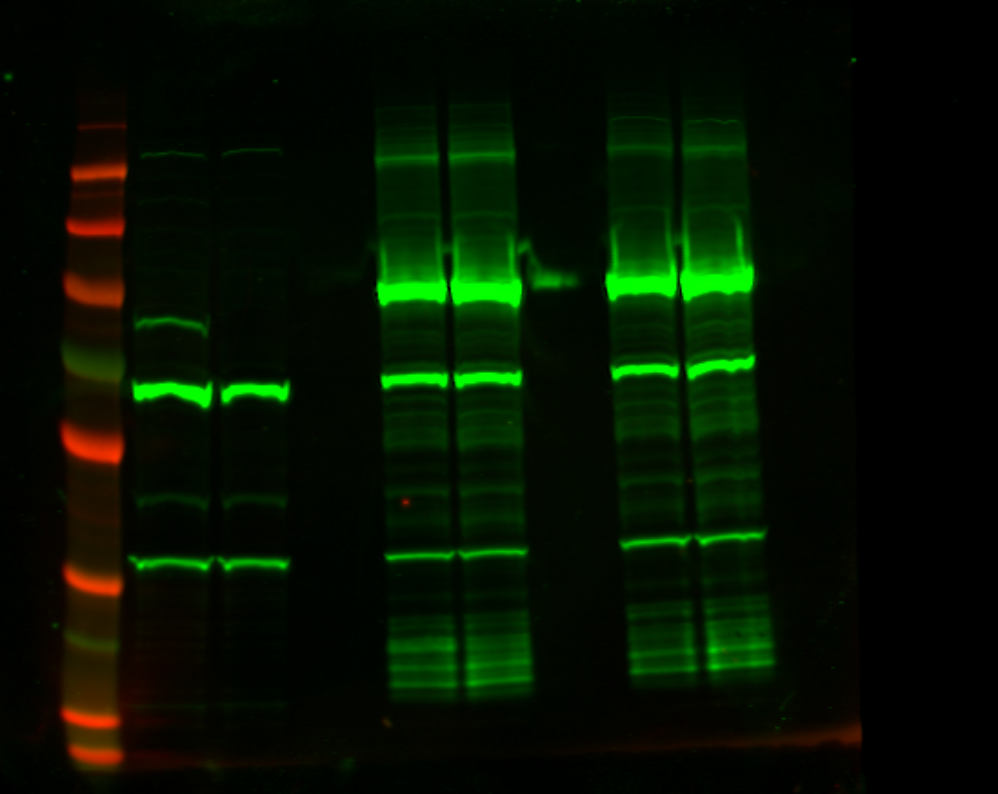

Supplement: Figure 7—figure supplement 1—source data 1. [file elife-90316-fig7-figsupp1-data1.zip › 20220731-TRMT1G3-T1KO T1flag Q530Nflag NSP5 transfection.tif]

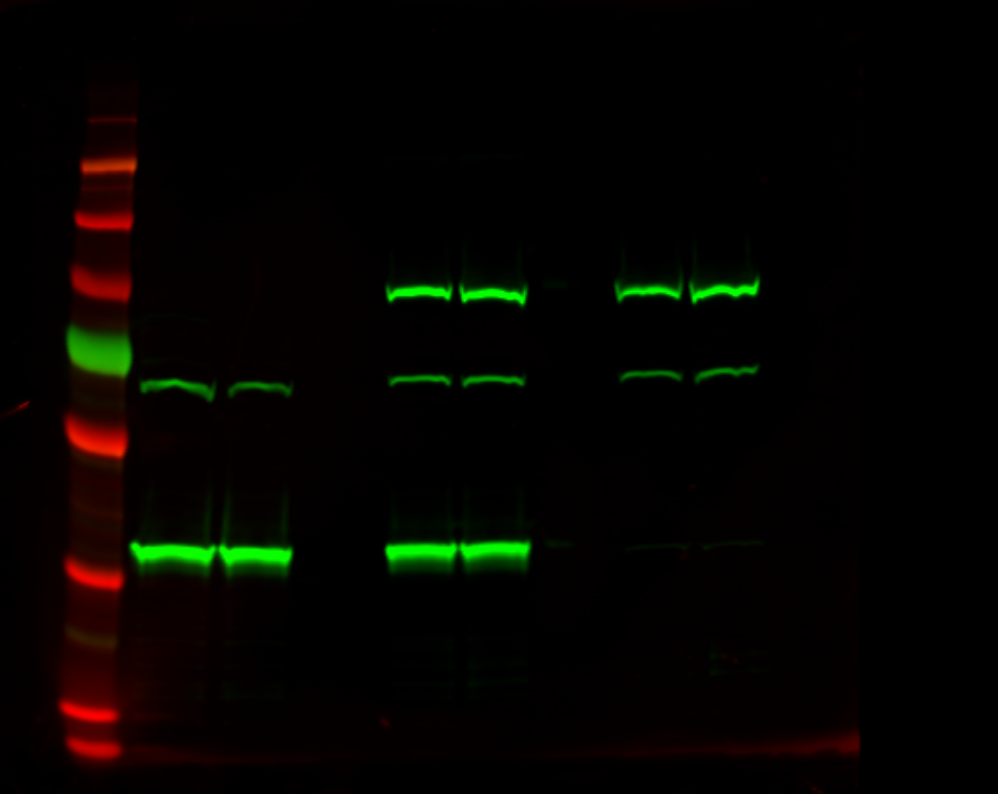

Supplement: Figure 7—figure supplement 1—source data 1. [file elife-90316-fig7-figsupp1-data1.zip › 20220801-Strep-T1KO T1flag Q530Nflag NSP5 transfection.tif]

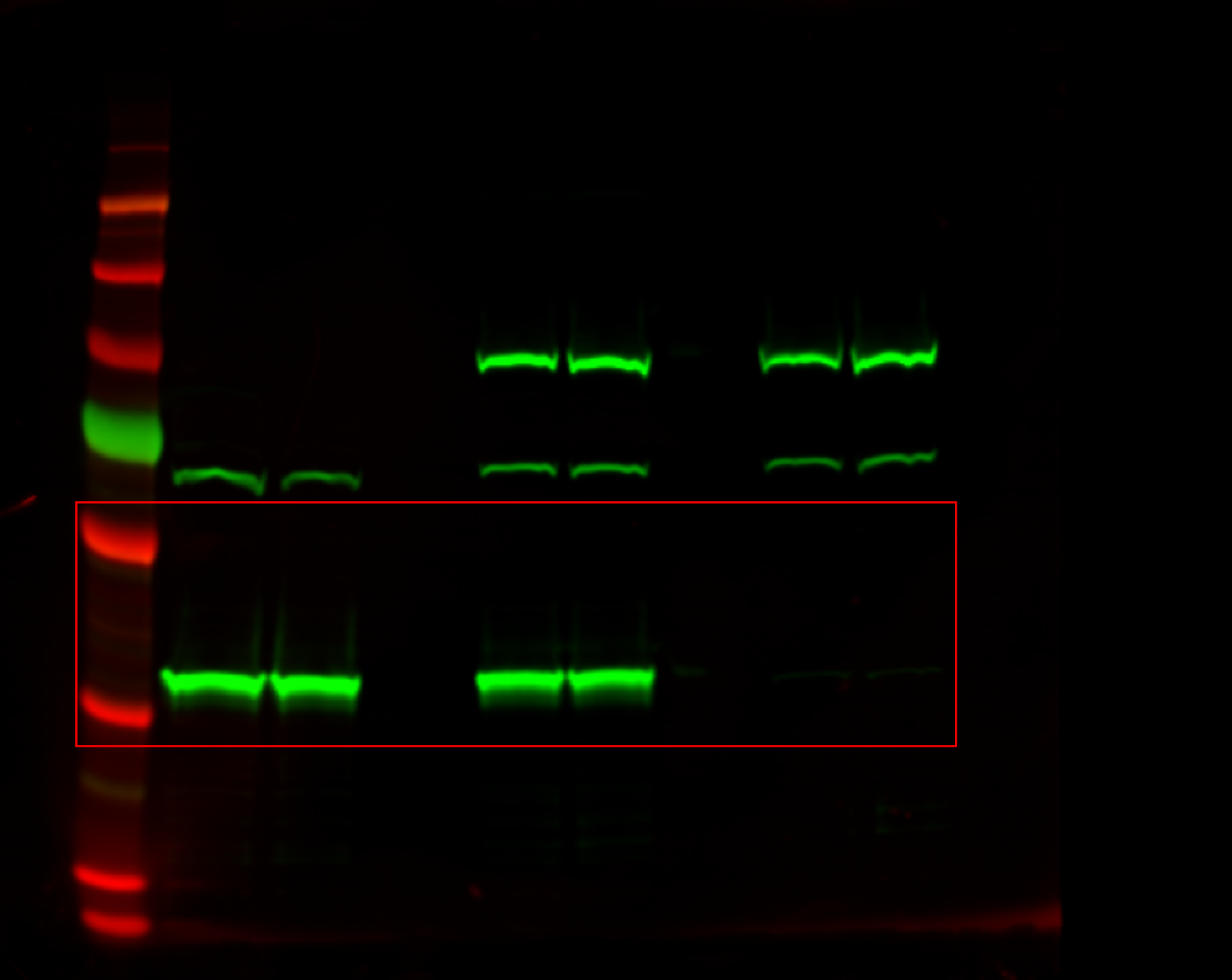

Supplement: Figure 7—figure supplement 1—source data 1. [file elife-90316-fig7-figsupp1-data1.zip › 20220801-Strep-T1KO T1flag Q530Nflag NSP5 transfection labeled.tif]

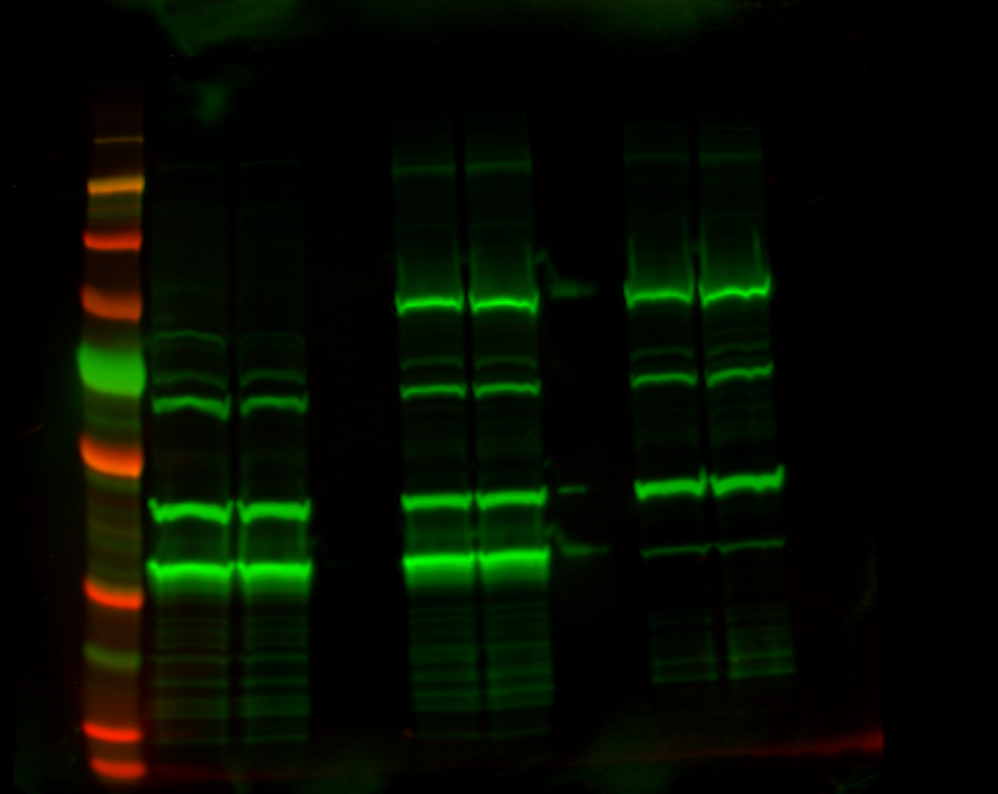

Supplement: Figure 7—figure supplement 1—source data 1. [file elife-90316-fig7-figsupp1-data1.zip › 20220802-Actin-T1KO T1flag Q530Nflag NSP5 transfection.tif]

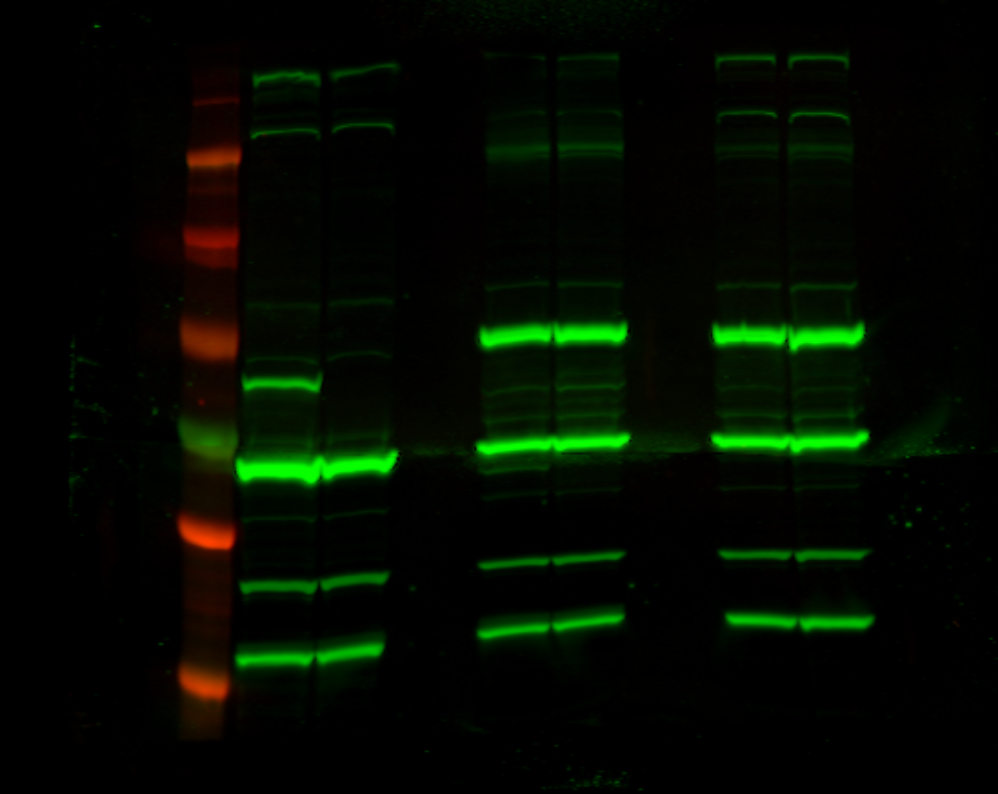

Supplement: Figure 7—figure supplement 1—source data 1. [file elife-90316-fig7-figsupp1-data1.zip › 20220812-TRMT1-G3-NSP5SctT1KOT1flagQ530Nflag-total.tif]

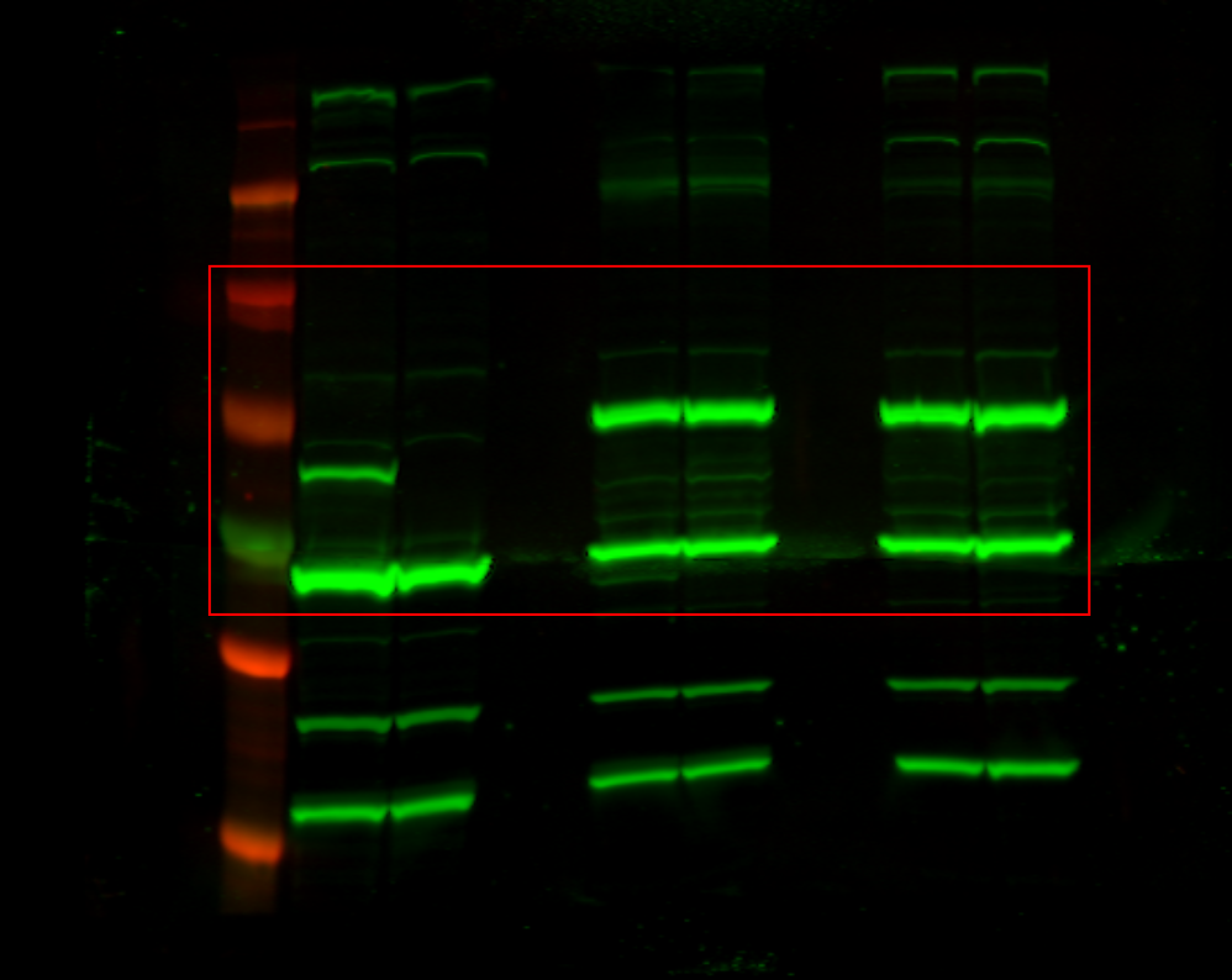

Supplement: Figure 7—figure supplement 1—source data 1. [file elife-90316-fig7-figsupp1-data1.zip › 20220812-TRMT1-G3-NSP5SctT1KOT1flagQ530Nflag-total labeled.tif]

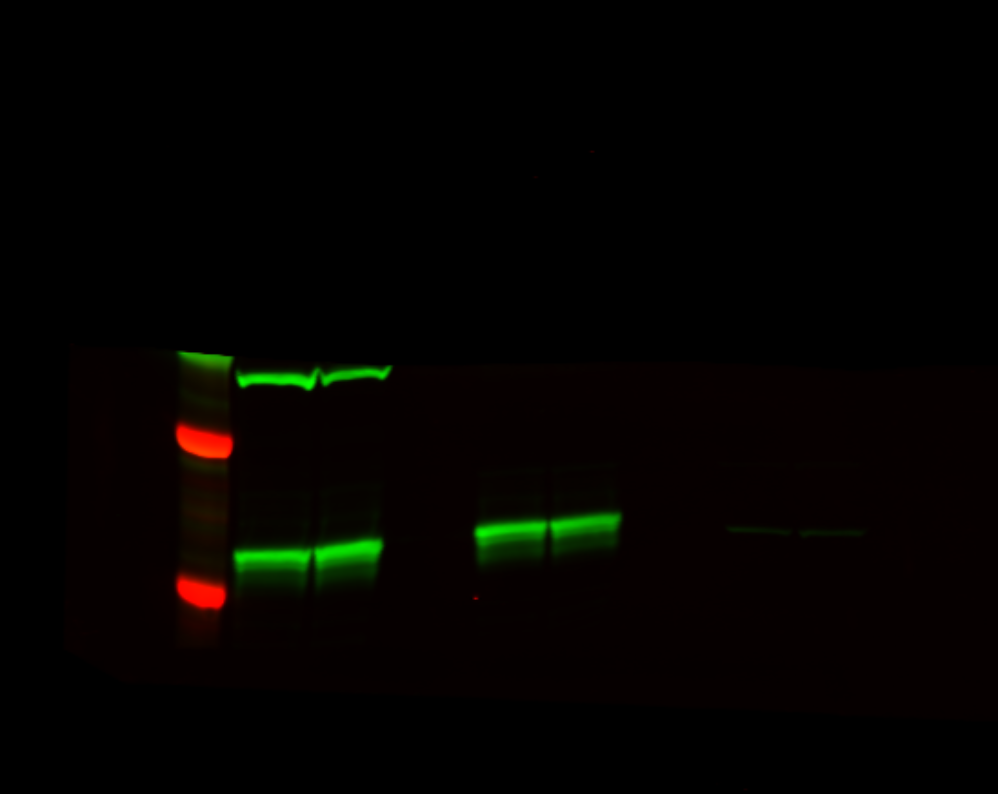

Supplement: Figure 7—figure supplement 1—source data 1. [file elife-90316-fig7-figsupp1-data1.zip › 20220814-Strep-NSP5SctT1KOT1flagQ530Nflag-bottom.tif]

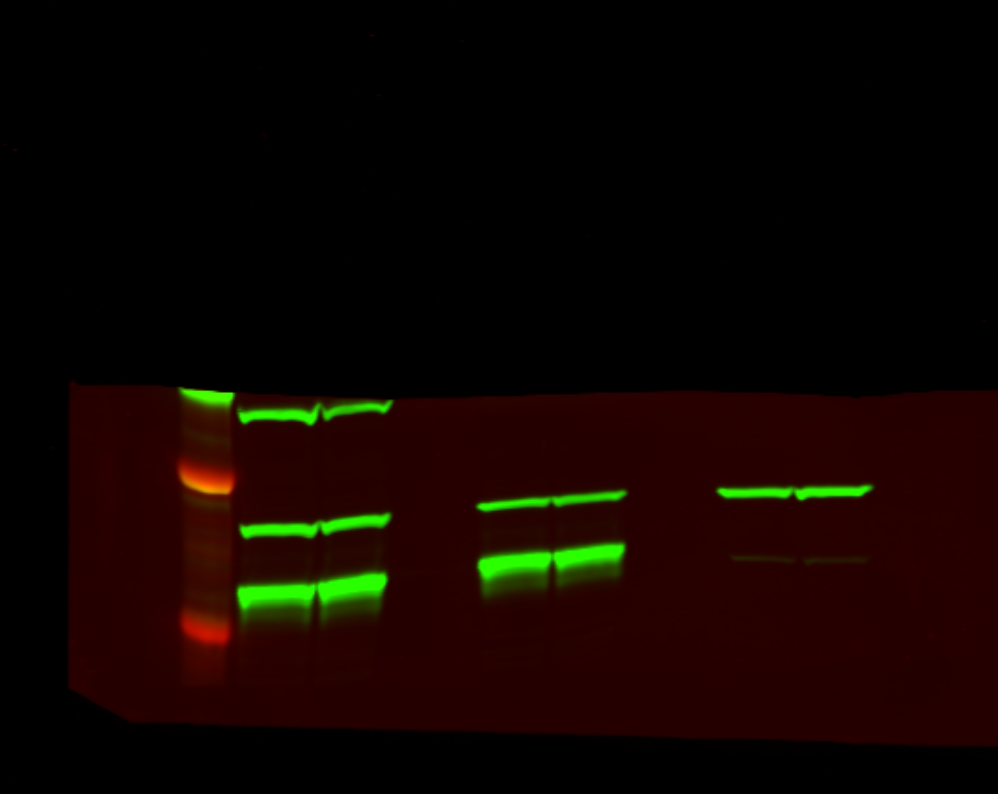

Supplement: Figure 7—figure supplement 1—source data 1. [file elife-90316-fig7-figsupp1-data1.zip › 20220816-Actin-NSP5SctT1KOT1flagQ530Nflag-bottom.tif]

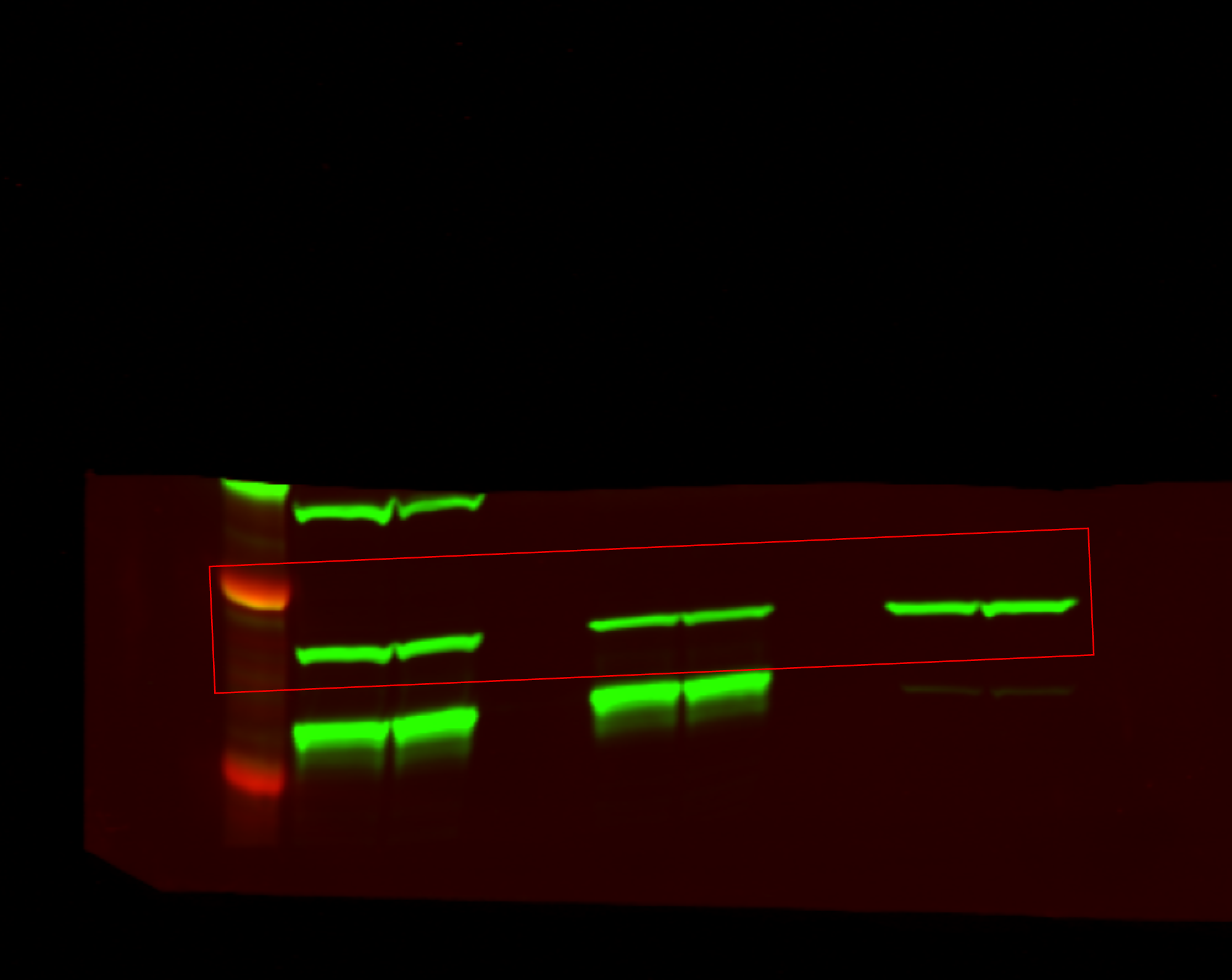

Supplement: Figure 7—figure supplement 1—source data 1. [file elife-90316-fig7-figsupp1-data1.zip › 20220816-Actin-NSP5SctT1KOT1flagQ530Nflag-bottom labeled.tif]

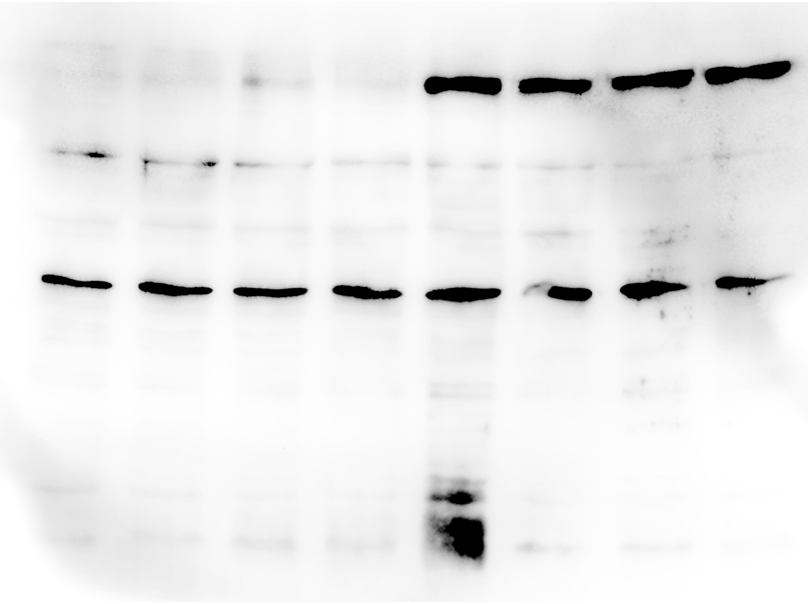

Supplement: Figure 7—figure supplement 2—source data 1. [file elife-90316-fig7-figsupp2-data1.zip › SFig6 blot ACE2.tiff]

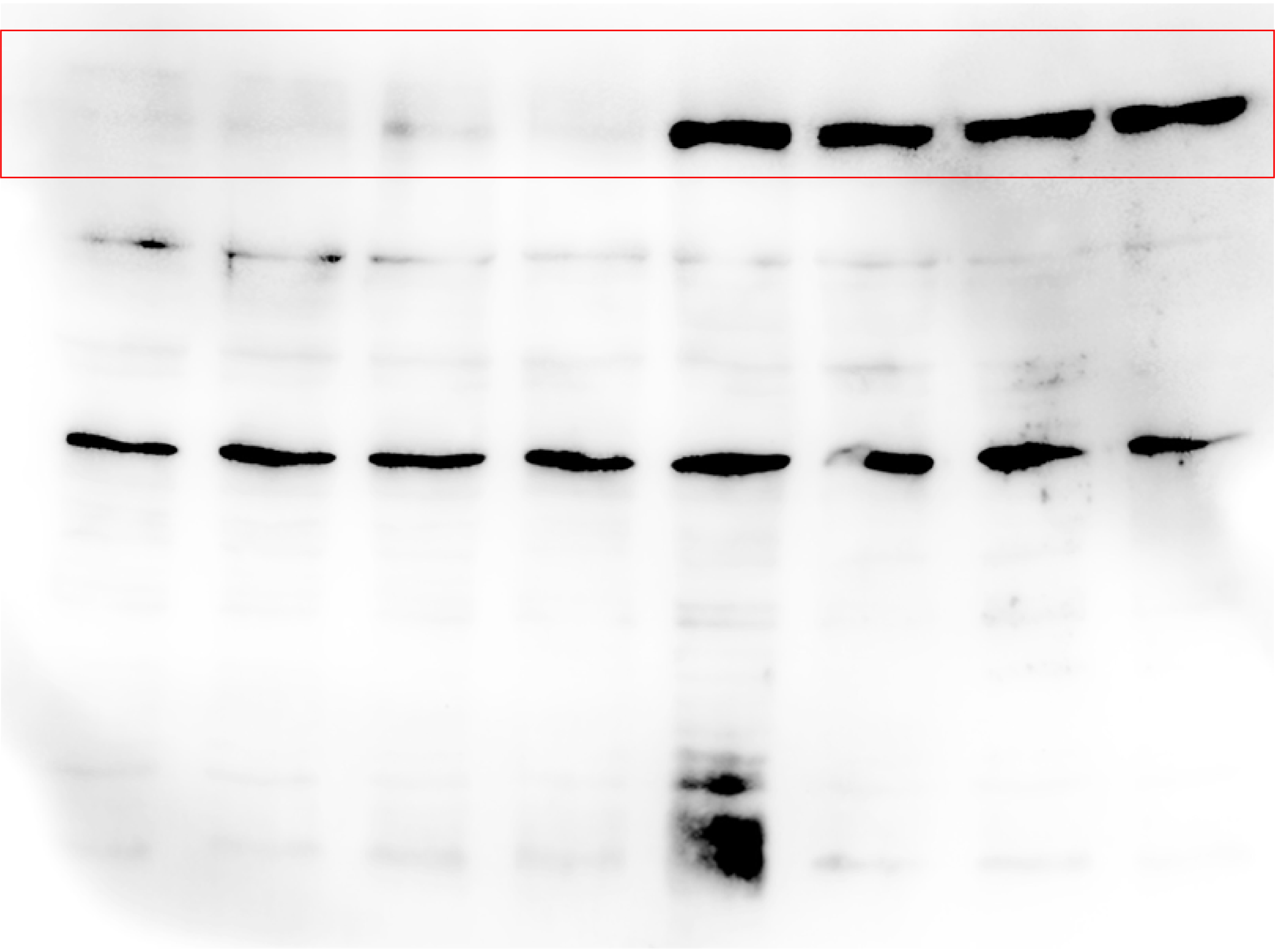

Supplement: Figure 7—figure supplement 2—source data 1. [file elife-90316-fig7-figsupp2-data1.zip › SFig6 blot ACE2 labeled.tif]

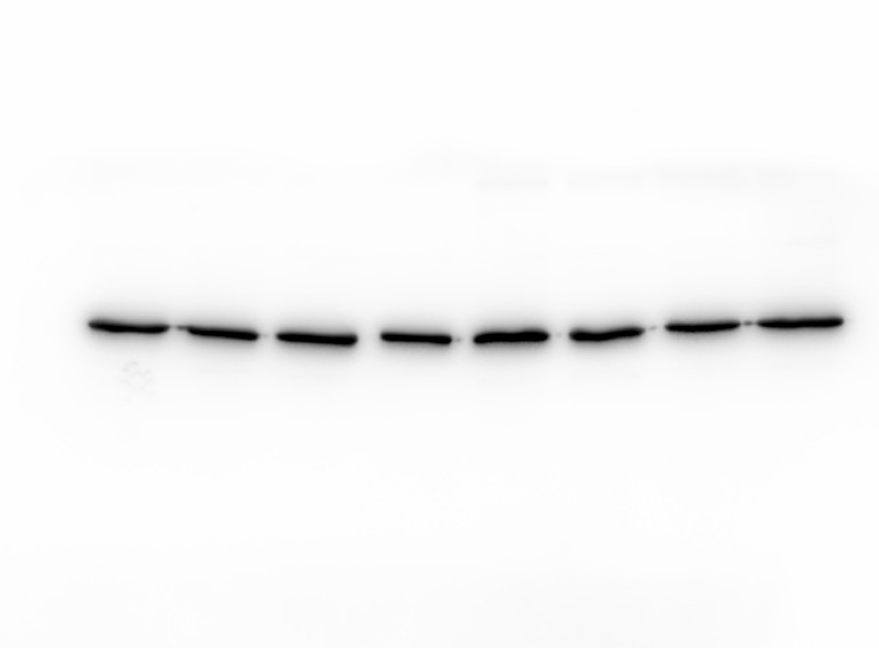

Supplement: Figure 7—figure supplement 2—source data 1. [file elife-90316-fig7-figsupp2-data1.zip › SFig6 blot actin.tiff]

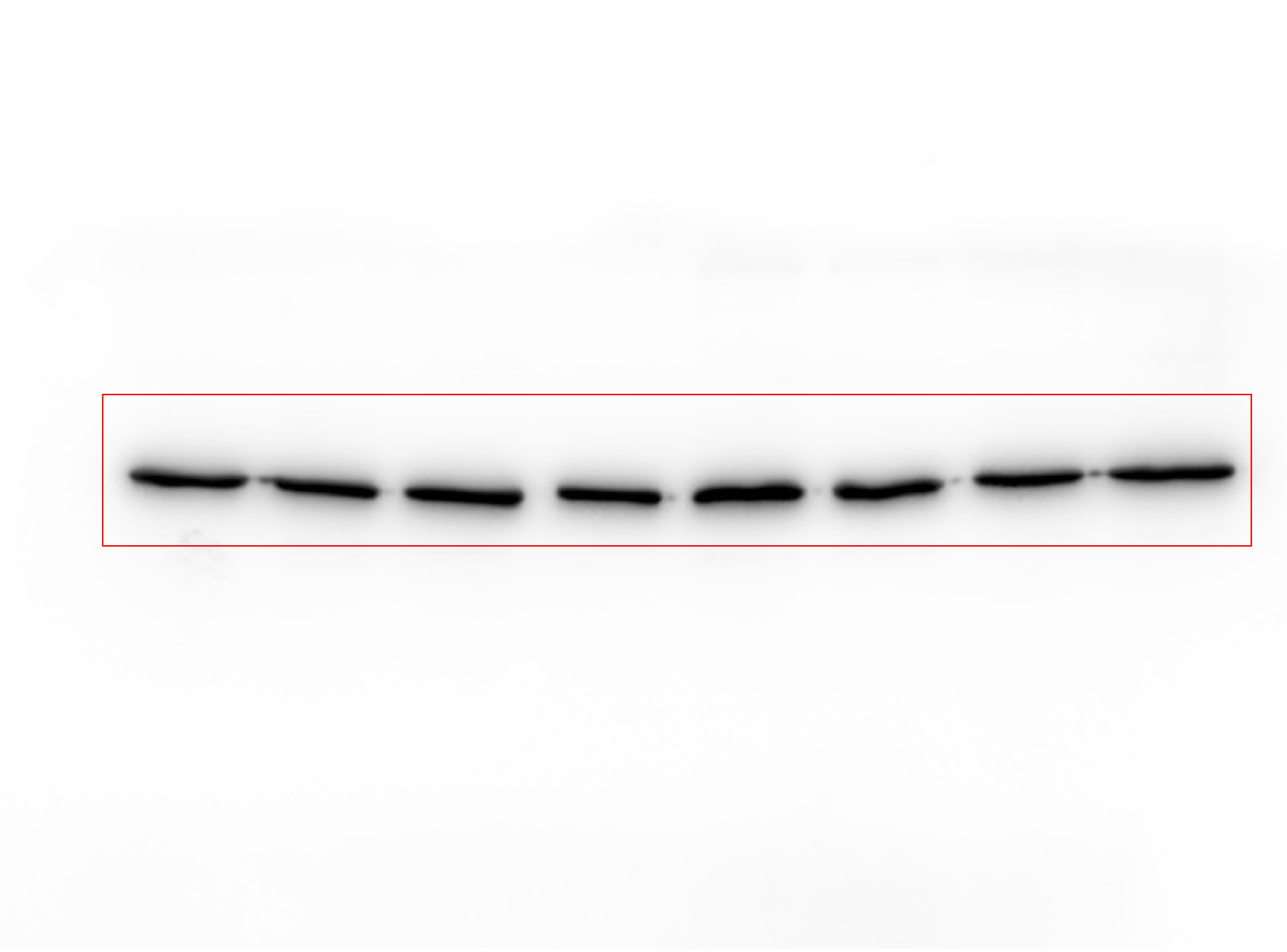

Supplement: Figure 7—figure supplement 2—source data 1. [file elife-90316-fig7-figsupp2-data1.zip › SFig6 blot actin labeled.tif]
